# Pyrite mega-analysis reveals modes of anoxia through geological time

**DOI:** 10.1126/sciadv.abj5687

**Published:** 2022-03-16

**Authors:** Joseph F. Emmings, Simon W. Poulton, Joanna Walsh, Kathryn A. Leeming, Ian Ross, Shanan E. Peters

**Affiliations:** 1 British Geological Survey, Keyworth, Nottingham NG12 5GG, UK.; 2 School of Geography, Geology and the Environment, University of Leicester, Leicester LE1 7RH, UK.; 3 School of Earth and Environment, University of Leeds, Leeds LS2 9JT, UK.; 4 Lyell Centre, British Geological Survey, Riccarton, Edinburgh EH14 4AS, UK.; 5 Ordnance Survey, Explorer House, Adanac Drive, Southampton SO16 0AS, UK.; 6 Department of Computer Sciences, University of Wisconsin–Madison, Madison, WI 53706, USA.; 7 Department of Geoscience, University of Wisconsin–Madison, Madison, WI 53706, USA.

## Abstract

The redox structure of the water column in anoxic basins through geological time remains poorly resolved despite its importance to biological evolution/extinction and biogeochemical cycling. Here, we provide a temporal record of bottom and pore water redox conditions by analyzing the temporal distribution and chemistry of sedimentary pyrite. We combine machine-reading techniques, applied over a large library of published literature, with statistical analysis of element concentrations in databases of sedimentary pyrite and bulk sedimentary rocks to generate a scaled analysis spanning the majority of Earth’s history. This analysis delineates the prevalent anoxic basin states from the Archaean to present day, which are associated with diagnostic combinations of five types of syngenetic pyrite. The underlying driver(s) for the pyrite types are unresolved but plausibly includes the ambient seawater inventory, precipitation kinetics, and the (co)location of organic matter degradation coupled to sulfate reduction, iron (oxyhydr)oxide dissolution, and pyrite precipitation.

## Teaser

Text mining and machine learning of sedimentary pyrite yield new perspectives on ancient marine anoxia.

## INTRODUCTION

The manifestation and extent of marine anoxia—including ferruginous (Fe-containing) and euxinic (sulfidic) states—exerted a first-order control on the evolution of life (*1*–*3*), and the development of many black shale–related mineral deposits through geological time (*4*–*6*). Reconstructing this history is therefore a key goal. For this reason, a wide variety of geochemical and modeling approaches have been taken to track the course of ocean redox evolution through time [see, e.g., (*7*–*10*)]. While this has led to major advances in our understanding of periods of biological innovation and extinction, a key limitation is the ability to integrate a wide variety of approaches to provide a consistent “reading” of the geological record through Earth’s history.

A prominent factor in terms of the response of the marine system to changes in ocean redox concerns the formation of pyrite (FeS_2_), which is commonly preserved in a variety of forms dictated by the precise local conditions encountered during its formation. Sedimentary pyrite formation occurs via reaction between reduced Fe and S, a process that is typically catalyzed in the marine environment during degradation of organic matter (OM) by sulfate-reducing microbes (*11*). Pyrite framboids precipitate relatively fast, under diffusion-limited conditions and from fluids at the supersaturation limit with respect to FeS or FeS_2_ (*12*, *13*). These conditions may exist in the water column and/or in pore waters, where sulfide typically persists for several centimeters to meters below seabed (*14*–*17*), depending on a variety of factors including sulfate and OM availability. By contrast, nodular and concretionary pyrite precipitation is a relatively slow process that occurs under advective or stagnant (poly)sulfidic conditions (*12*), conditions that tend to exist during non–steady-state fluid dewatering (*18*) of sulfidic sediments near the seabed.

The advantage of considering pyrite morphology as a means to reconstruct basinal redox history is that the presence and nature of pyrite in ancient marine sediments have been extensively documented over many years. Here, we provide a new perspective on marine anoxia in the geological record by deploying machine-reading techniques over a large library of scientific publications, which we integrated with geochemical analyses of coeval shales (*19*–*25*). We searched for phrases related to the type of pyrite observed in sediments, including pyrite framboids, concretions, and nodules. Our objective is to use machine-reading outputs coupled to black shale pyrite chemistry to evaluate the modes of marine anoxia through geological time.

## RESULTS

### Machine reading

**Fig. 1.**
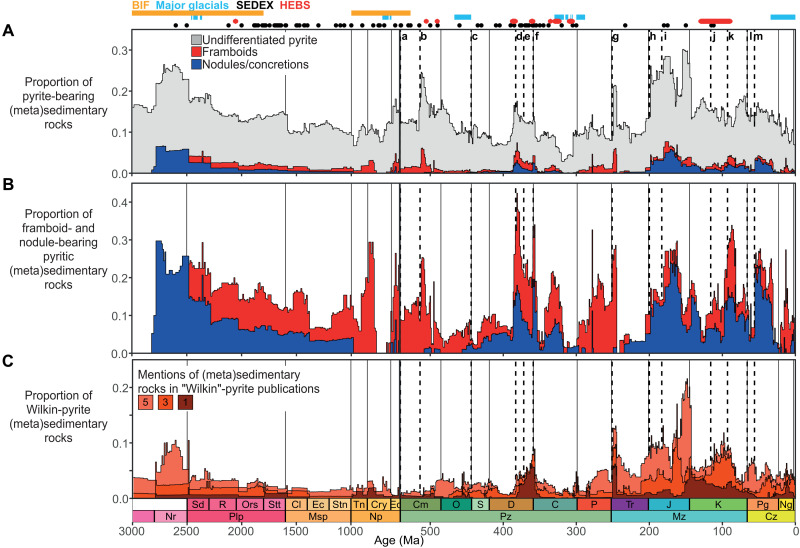
Stacked abundances of pyrite-bearing sedimentary and metasedimentary rocks through geological time. (**A** and **B**) Pyrite framboid– and nodule-bearing rocks expressed as proportions of all (meta)sedimentary rocks (A) and all pyrite-bearing (meta)sedimentary rocks (B). Mentions of pyrite extracted from the xDD library were matched, using the CoreNLP pipeline (*27*, *31*), to stratigraphic names recorded in Macrostrat, which includes the Geoscience Australia and British Geological Survey stratigraphic databases. Propagation of Macrostrat units yields a record with increased temporal resolution and is weighted to the geographic extent of each sedimentary rock package in the Macrostrat focal area. Each “unit” is a geographically distinct entity (*32*), meaning propagation of units increases bias toward the Macrostrat focal area (primarily North America). Omission of Macrostrat units yields a record that is less biased toward the Macrostrat focal area at the expense of temporal resolution and represents a spatially unweighted record (fig. S3). (**C**) Mentions of (meta)sedimentary rocks in publications that also cite seminal pyrite framboid size proxy publications authored or coauthored by J. Wilkin [see, e.g., (*38*–*40*)]. Phanerozoic (0 to 541 Ma) and Precambrian (541 to 3000) bin widths are 1 and 10 Ma, respectively. International chronostratigraphic intervals (lines and thick black and white bars) and United States Geological Survey sedimentary-exhalative (SEDEX) Pb-Zn global deposits, highly enriched metalliferous black shales (HEBS), Precambrian banded iron formations (BIFs), and the major glaciations are shown for reference (see Materials and Methods for underlying references). Some or all SEDEX deposits are not necessarily syngenetic sensu stricto (*33*). Labels a to m refer to 11 haline euxinic acidic thermal transgression (HEATT) episodes (*2*), using updated ages reported in the Macrostrat database, plus two additional extinction events associated with the end-Devonian crisis (*34*, *35*): Ediacaran-Cambrian (a), Botomian (b), Late Ordovian (c), Givetian-Frasnian (d), Frasnian-Famennian (e), Hangenberg (Devonian-Mississippian) (f), end-Permian (g), end-Triassic (h), Toarcian-Pliensbachian (i), Aptian (j), Cenomannian-Turonian (k), Cretaceous-Paleogene (l), and Paleocene-Eocene thermal maximum (m).

Text mining was implemented using the GeoDeepDive (now known as xDD) digital library and machine-reading system (*26*) coupled to an algorithm that decomposes sentences into speech and linguistic components using Stanford natural language processing (NLP; fig. S1) (*27*). The algorithm matches phrases (such as “pyrite nodule”) extracted from the xDD library (10,661,918 documents at the time of analysis) to stratigraphic names recorded in the Macrostrat stratigraphic database (*28*–*30*), with an effective accuracy of at least 87% (see the Supplementary Materials for accuracy estimates) (*31*). A total of 10,320 phrases were matched to 1794 unique stratigraphic names, including 140 framboid bearing and 110 pyrite nodule or concretion bearing (here simplified as “nodule bearing”), defining a temporal record of pyrite-bearing metasedimentary and sedimentary rocks (Fig. 1, A and B). Normalization of the framboid and nodule record to all pyrite-bearing metasedimentary and sedimentary rocks (Fig. 1B) adjusts for long-term [>10 million years (Ma)] drift in the record relating to plate tectonic regulation of basin type and extent and therefore, by extension, the types of sediments preserved (*36*). The majority (ca. 83%) of pyrite framboid– and nodule-bearing rocks are siliciclastic and dominantly fine-grained (fig. S6). Approximately 83% of the pyrite framboid– and nodule/concretion-bearing rocks are described as shale, mudstone, argillite, or other equivalent fine-grained rock types, including interbedded variants. Other pyrite framboid–and nodule/concretion-bearing rocks include pure sandstones and conglomerates (ca. 6%), limestones, dolomites and cherts (ca. 3%), and interbedded variants (ca. 8%). Overall, this is consistent with observations that fine-grained, siliciclastic sedimentary rocks are the primary host for both pyrite framboids (*12*) and pyrite nodules/concretions (*37*).

We also searched for publications in the xDD library containing stratigraphic names indexed in the Macrostrat stratigraphic database and which cite one or more of the formative pyrite framboid size distribution publications authored or coauthored by J. Wilkin [Fig. 1C; e.g., (*38*–*40*)]. By definition, this supplementary approach does not contain false negatives but likely includes false positives. In cases where publications mention one stratigraphic package, the Wilkin-pyrite record is best interpreted as a crude alternative measure for the proportion of pyrite-bearing rocks in the focal area. At increasing numbers of stratigraphic packages mentioned in each publication (up to 5), we interpret the Wilkin-pyrite results as a measure for the level of attention each part of the stratigraphic column has received in terms of framboidal pyrite and/or paleoredox research.

The extracted pyrite records do not directly correspond to the temporal distribution of pyritic rocks exhibiting “mineralization” or containing “veins” (fig. S7D) or evaporitic sedimentary rocks in the Macrostrat focal area (*29*) or generally (fig. S7E). This precludes intraunit or local late diagenetic processes, such as thermochemical sulfate reduction (*41*) or hydrothermal mineralization (*42*), as an explanation for the temporal distribution of pyrite framboids, nodules, and concretions. Therefore, the text-mining pyrite record is interpreted as a proxy for the prevalence of changing bottom water and/or shallow early diagenetic pore water conditions. Although we recognize open-system, late diagenetic effects remain unconstrained. This interpretation is consistent with the requirement for anoxic, sulfidic conditions to induce pyrite framboid formation during syngenesis and/or early diagenesis (*39*). Pyrite nodules are also commonly considered to nucleate and/or form entirely during early diagenesis in many black shale successions (*37*).

In the Precambrian, pyrite framboids and nodules are rare in rocks before ca. 2.8 billion years (Ga) (Fig. 1), consistent with sulfur-limited anoxic conditions (*9*, *43*). After ca. 2.8 Ga, the relative abundance of pyrite nodules rapidly increases, coincident with enhanced production of oxygen (*44*) before the Great Oxidation Event (GOE) at ca. 2.4 to 2.2 Ga [see, e.g., (*45*)]. This likely indicates increased sulfate input to the oceans, following pyrite oxidation on the continents, and subsequent reprecipitation in anoxic bottom waters and/or pore waters (*46*). Following the GOE, the proportion of undifferentiated pyrite-bearing and nodule-bearing rocks (Fig. 1) declines toward the end of the Mesoproterozoic (Fig. 1). This may reflect the initial onset of euxinia after ca. 2.20 to 1.84 Ga (*43*, *47*), which appears to have progressively contracted through the Mesoproterozoic, possibly due to extensive global burial of pyrite sulfur in persistently anoxic oceans (*48*, *49*). At the onset of the Neoproterozoic, the interval between ca. 0.98 and 0.77 Ga is defined by the disappearance of pyrite nodules from the text-mining record, coincident with a return to global ferruginous ocean conditions (*50*). An initially low but increasing proportion of framboid-bearing rocks during the earliest Neoproterozoic (Tonian) is potentially linked to low to moderate rates of productivity (*51*). A high proportion of framboid-bearing rocks at the end of the Tonian may support the hypothesis of elevated rates of microbial sulfate reduction, coupled to pyrite burial, ahead of Snowball Earth glaciations (*52*, *53*). A near absence of pyrite framboid–and nodule/concretion-bearing rocks in the interval between ca. 0.77 and 0.66 Ga partly coincides with the Sturtian glaciation (Fig. 1) (*54*). Overall, the relative paucity of sedimentary rocks and mentions of pyrite (fig. S4) in the Precambrian, however, suggests that the text-mining outputs may be uncertain.

The Phanerozoic pyrite record (Fig. 1, A and B) varies between two end members. Framboid-bearing rocks are abundant from the Cambrian to the middle Ordovician, from the Permian to the early Triassic, and from the Neogene to the present day, but these intervals generally lack pyrite nodule–bearing rocks. By contrast, pyrite nodule–bearing rocks are abundant from the mid-Ordovician to the late Carboniferous and from the Triassic to the late Paleogene. These intervals typically exhibit an intermediate proportion of framboid-bearing rocks. Step changes and peaks in the abundances of pyrite framboid– and/or nodule-bearing rocks follow or coincide with the widely recognized Phanerozoic haline euxinic acidic thermal transgression (HEATT) episodes (*2*). The Wilkin-pyrite record (Fig. 1C) suggests that the late Devonian–early Carboniferous and Mesozoic intervals are strongly pyrite bearing and/or have received the most attention in terms of sedimentary pyrite research. The Cambrian is linked to few Wilkin-pyrite publications (Fig. 1C) but exhibits a relatively high abundance of framboid-bearing rocks (Fig. 1, A and B). This may suggest that the Cambrian is extremely framboid bearing, given the relative amount of research that has been conducted in this interval.

### Pyrite chemistry

**Fig. 2.**
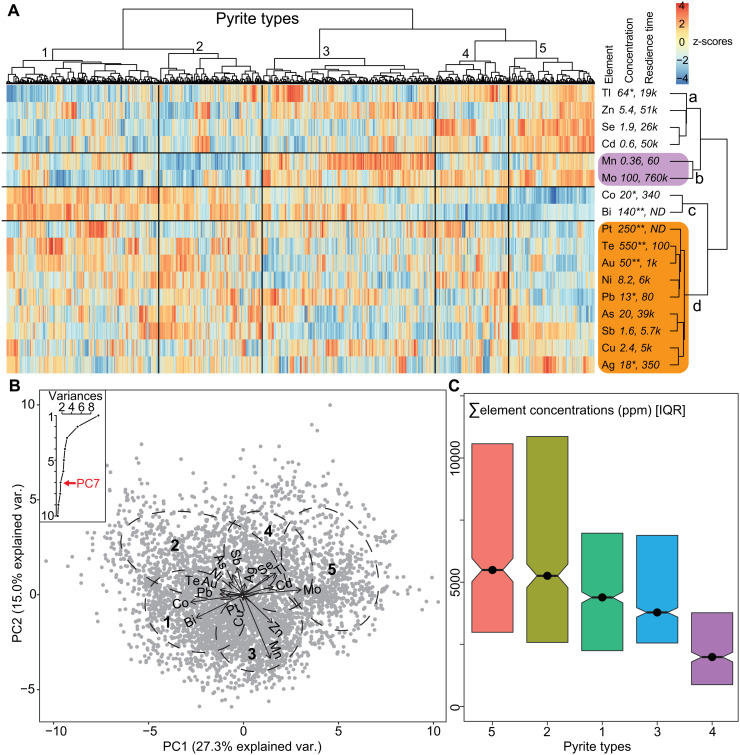
Multivariate statistical analysis for *clr*-transformed pyrite trace element concentrations (*19*–*23*). (**A**) HCA by individual pyrite analyses (*x* axis, *n* = 4360) and elements (*y* axis). Italicized values indicate average modern ocean element concentrations (* indicates picomol; ** indicates femtomol; all others, nmol) and residence times (in years) (see Materials and Methods). See text for explanation of clusters a to d (elements) and pyrite types 1 to 5 (samples). ND, not detected. (**B**) Principal components analysis scree plot (inset) and biplot of PC1 and PC2, including 68% probability ellipses for pyrite clusters 1 to 5. The scree plot shows the explained variance attributed to each principal component (PC) and exhibits a large elbow at PC3 and a smaller elbow at PC7. PC7 was selected on the basis of an appropriate level of complexity for machine learning (see Materials and Methods). (**C**) Notched interquartile ranges (IQRs) and median total trace element concentrations for pyrite clusters 1 to 5. Notches delineate the 95% (2σ) confidence interval around the median.

Next, we investigate the potential significance of a growing syngenetic to early diagenetic pyrite trace element dataset spanning the Archaean to present day (*19*–*23*). Hierarchical cluster analysis (HCA) for *clr*-transformed (*55*) trace element concentrations in syngenetic pyrite reveals five types of pyrite associated with four element associations (Fig. 2). We explore three explanations for the pyrite clusters: (1) uptake from ambient seawater, representing an inventory of trace elements that likely varied through time (*22*); (2) pyrite reaction kinetics (*12*), where fast pyrite growth promotes uptake of incompatible elements (*56*); and (3) the loci of sulfate reduction, Mn and Fe oxyhr(oxide) reduction, and pyrite precipitation (Mn-Fe-OM dynamics).

Trace element contents in pyrite are not clearly clustered in terms of concentrations or residence times in modern seawater (Fig. 2), and most pyrite types are temporally ubiquitous (see Discussion). This weakens the credibility of, but does not preclude, an ambient seawater control on the pyrite types (*22*). The pyrite clusters are potentially explained by reaction kinetics, where enrichment of incompatible elements in pyrite types 3 and 5 (Zn, Se, Cd, Mn, Tl, and Mo) suggests fast precipitation (*56*). Conversely, enrichment of generally compatible elements in pyrite types 2 and 4 (Bi, Te, Sb, Ag, Cu, Pb, Ni, and As) suggests sluggish reaction kinetics. Co is an exception, and type 1 (and, to some degree, type 4) pyrite represents an intermediate in this scenario.

The role of organic substrates and/or Mn-Fe (oxyhydr)oxides as vectors for metal transfer into pyrite is consistent with observations from the modern Cariaco Basin, where unconservative elements are concentrated in syngenetic pyrite by a factor of 1000 to 10,000 compared to average modern seawater, in contrast to 0.1 to 100 for nutrients and conservative elements (*19*–*23*). This indicates nonlinear uptake of elements, with syngenetic pyrite precipitation coupled to at least one additional component other than ambient seawater (i.e., Mn-Fe-OM dynamics). This is consistent with the redox structure of the Cariaco Basin, a productive and weakly euxinic basin associated with a Mn-Fe (oxyhydr)oxide particulate shuttle (*57*). Clustering of Cd and Zn could indicate biological uptake and particulate OM (POM) trapping of cluster “a” elements in sulfidic microenvironments (*58*). Mn and Mo clustering could support dissolution of Mn (oxyhydr)oxides as an important vector for Mo (*59*). Co and Bi chelate with labile OM [here termed dissolved OM (DOM)] (*60*, *61*), and Co is a key micronutrient for cyanobacteria (*62*). Remaining elements (cluster “d”) are hybrid or unconservative, which in the marine environment primarily adsorbs onto Fe (oxyhydr)oxides [see, e.g., (*63*, *64*)] and, in some cases, also chelate with DOM [see, e.g., (*65*)].

Hypothesis (3) suggests that type 5 pyrite is the POM end member because it is associated with enrichment in Tl, Zn, Se, Cd, and Mo and, to a lesser extent, elements associated with Fe (oxyhydr)oxides (cluster d). Elevated Mo enrichment compared to Mn in type 5 pyrite (Fig. 2, A and B) suggests multiple Mo fixation mechanisms, perhaps including Mn (oxyhydr)oxide vectors and dissolved MoO_4_^2−^ (*65*). By contrast, pyrite types 1 and 2 could represent the DOM end members on the basis of Bi and Co enrichment. Patterns of enrichment in cluster d elements suggest that Fe (oxyhydr)oxides were more important substrates during precipitation of type 2 pyrite compared to type 1 pyrite. By extension, pyrite type 1 potentially precipitated within DOM microenvironments exposed primarily to dissolved Fe and not Fe (oxyhydr)oxides.

Pyrite type 3 is Mn enriched, depleted in cluster d elements, and inconsistently enriched in Tl, Zn, Se, Cd, Co, and Bi. Since Mn (oxyhydr)oxides dissolve under sulfidic conditions (*59*), Mn enrichment in type 3 pyrite is best explained by pyrite precipitation in euxinic waters enriched in dissolved Mn, Fe, and other metals and lacking Mn-Fe (oxyhydr)oxides. Thus, type 3 pyrite may indicate strongly euxinic conditions that supported or followed Mn-Fe (oxyhydr)oxide reduction. By contrast, Mn depletion and generally mixed element enrichment including Tl, Zn, Se, and Cd in type 4 pyrite suggest precipitation under POM-enriched, weakly euxinic, or incipient early diagenetic sulfidic ambient conditions, which did not induce full dissolution of ambient phases reactive to H_2_S [e.g., Mn (oxyhydr)oxides and magnetite]. Subdued availability of metals derived from ambient H_2_S-reactive phases potentially explains the low total trace element quotas in type 4 pyrite (Fig. 2C).

**Fig. 3.**
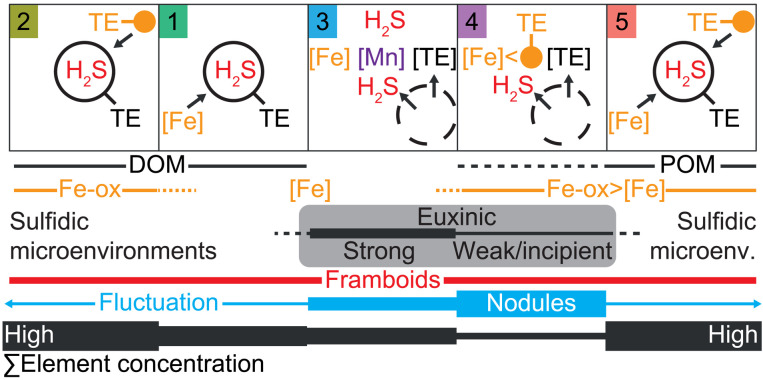
Hypothesized pyrite-type Fe-OM dynamics based on trace element concentrations in pyrite (*19*–*23*). Pyrite framboids precipitate relatively fast under diffusion-limited conditions and from fluids at the supersaturation limit with respect to FeS or FeS_2_. These conditions may exist within microenvironments, ambient bottom waters, or pore waters. By contrast, nodular and concretionary pyrite precipitation is a relatively slow process that occurs under advective or stagnant (poly)sulfidic conditions, associated with incipient or fluctuating-free sulfide in pore waters. See main text for explanation of pyrite types 1 to 5. POM, particulate organic matter; DOM, dissolved organic matter; TE, trace element.

From the perspective of Mn-Fe-OM dynamics, sequences of 2-1-3 and 5-4-3 pyrite types could represent idealized DOM- and POM-driven transitions from nonsulfidic to euxinic water column conditions, respectively (Fig. 3). Under euxinic conditions, diffusion of redox-sensitive elements across seabed into early diagenetic phases (*65*) also potentially contributed to the general trace element deficiency in pyrite types 3 and 4 (Fig. 2C). Last, all three mechanisms [hypotheses (1) to (3)] potentially operated synergistically or in tandem. For example, the concentration of H_2_S in POM microenvironments (type 5) and ambient seawater (type 3) may induce fast pyrite precipitation (*12*) and, by extension, uptake of the same suite of POM-associated or incompatible elements into pyrite (*56*). Periods of low ocean or atmospheric oxygen could modify the balance of element mobility and availability in the environment (*22*), a phenomenon that is expected to support larger standing stock of DOM [see, e.g., (*66*)] and where both mechanisms may favor uptake of Co (pyrite types 1 and 2).

### Charting the pyrite types through time

**Fig. 4.**
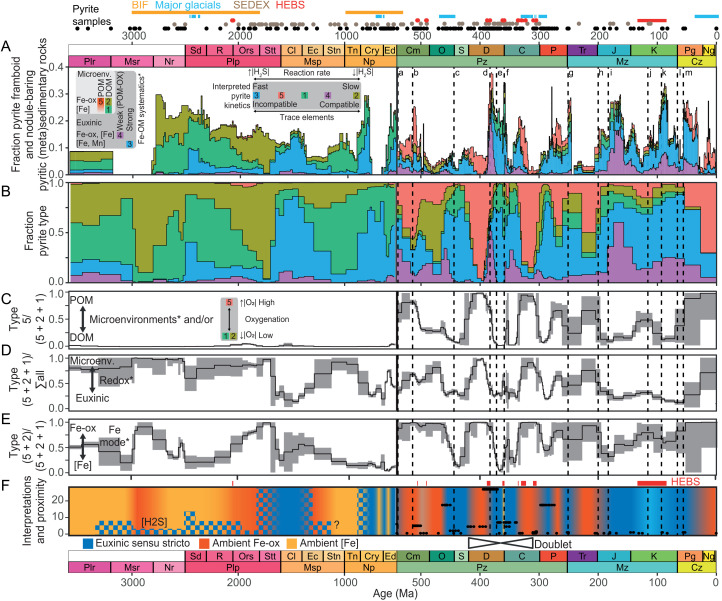
The results of text-mining outputs integrated with machine learning of pyrite TE concentrations (*19*–*23*). (*) The five pyrite types are interpreted primarily in terms of Mn-Fe-OM dynamics, but there are alternative hypotheses for TE clustering in pyrite, such as reaction kinetics coupled to element compatibility (*12*, *56*) and element mobility coupled to ocean-atmosphere oxygenation (*22*). Fractions of (**A** and **B**) pyrite types 1 to 5 through geological time, including (A) modulation by the proportion of pyrite framboid– and nodule-bearing (meta)sedimentary rocks (Fig. 1B). (**C**) Pyrite type 5 versus pyrite types 5 + 2 + 1. (**D**) Fraction of pyrite types 5 + 2 + 1. (**E**) Pyrite types 5 + 2 versus 5 + 2 + 1. A similar result is obtained excluding the type 5 pyrite fraction (fig. S20). (**F**) Interpreted, generalized anoxic basin ambient phases and conditions local to sites of pyrite precipitation based primarily on Mn-Fe-OM dynamics. The Phanerozoic includes the sample spatial proximities (see Materials and Methods), where high values indicate increasing bias toward one basin or area (refer to fig. S23 for the key to the basins). Thus, in most or all cases, the interpretations do not represent oceanwide conditions. Pyrite-type vertical error bars (C to E) represent the propagated uncertainty (95% confidence intervals). Bin widths are inversely proportional to the pyrite sample temporal resolution. The maximum temporal resolution was fixed at 1 Ma (Phanerozoic) and 10 Ma (Precambrian). Each pyrite sample is a discrete pyrite analysis (*19*–*23*). See Fig. 1 for explanations of the HEATT episodes a to m and sedimentary mineral deposits (SEDEX, BIF, and HEBS).

**Fig. 5.**
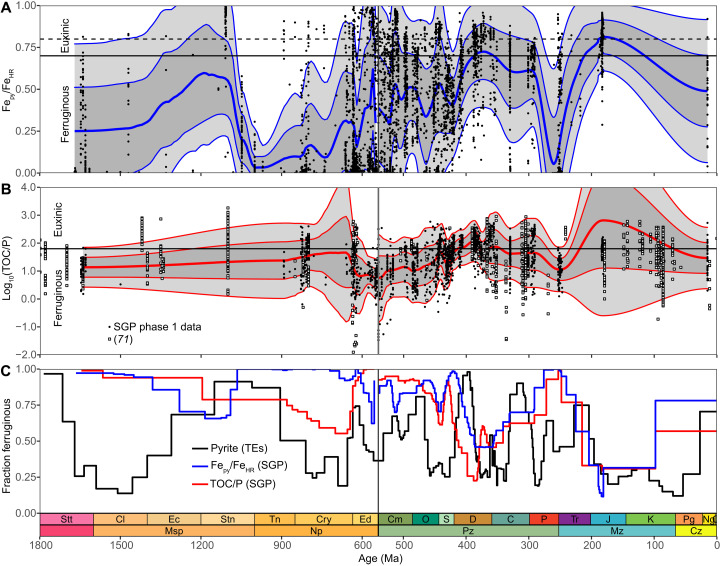
Comparison of the classic bulk geochemical proxies for ferruginous anoxic versus euxinic paleoredox conditions versus the pyrite TE record through geological time. (**A** and **B**) Local polynomial regression prediction intervals (median, 1σ, 2σ) for Fe speciation (A) and TOC/P (B) ratios recorded in the Sedimentary Geochemistry and Paleoenvironments (SGP) phase 1 database (*24*, *25*). The dataset was culled for samples which exhibit total Fe (Fe_T_) > 0.5 wt % (*67*) and the ratio of highly reactive Fe (Fe_HR_) to total Fe (Fe_T_) above the oxic-anoxic threshold of 0.38 (*8*, *68*, *69*). The ratio of pyrite Fe (Fe_py_) to highly reactive Fe (Fe_HR_), Fe_py_/Fe_HR_, > 0.7 represents a key threshold for identifying euxinia (*8*, *68*, *69*). TOC/P < 63 [equivalent to molar TOC/P of 50; (*70*)] also delineates ferruginous versus euxinic conditions. (B) TOC/P data for anoxic sediments from (*71*) (not included in the regression). (**C**) Fractions of rocks deposited under euxinic versus ferruginous conditions based on ratios of Fe_HR_/Fe_T_ and TOC/P (A and B) and the fraction of pyrite types 5 + 2 + 1 (as in Fig. 4D).

Next, we deployed *k*-nearest neighbor (*k*NN) machine learning of the pyrite clusters coupled to local polynomial regression (LOESS) of principal components (PCs) 1 to 7 (see Materials and Methods) to map the five pyrite types through geological time (Fig. 4). We linked the xDD and machine learning of pyrite types in time. In general, the pyrite observations derive from a single basin or location at any given time (figs. S13 and S14), meaning multi-increment trends may represent long-term intrabasinal or interbasinal variation. Sample spatial proximities (see Fig. 4F, fig. S23, and Materials and Methods) show that some intervals represent a dense stack of observations from the same or adjacent basins, whereas, in other cases, the record is more dispersed at regional to global scales. An assumed Fe-Mn-OM dynamics control on the composition of pyrite also favors generally localized or regional, but not global, redox interpretations. In this scenario, the fraction of type 5 pyrite versus types 1 and 2 pyrite reflects the proportion of POM versus DOM microenvironments (Fig. 4C). This ratio could also proxy for element mobility in the environment because it aliases Se/Co (Fig. 2A), previously interpreted in terms of global ocean-atmosphere oxygenation (*22*). In terms of the Mn-Fe-OM hypothesis, the cumulative proportion of pyrite types 1, 2, and 5 represents the ratio of precipitation in microenvironments versus euxinia sensu stricto (Fig. 4D) and, by extension, the proportion of ferruginous versus euxinic conditions. The ratio of pyrite types 2 and 5 versus type 1 pyrite may proxy for the fraction of ambient Fe (oxyhydr)oxides versus dissolved Fe (Fig. 4E). Last, from the Mesoproterozoic to the present day, we compared the pyrite-derived fraction of ferruginous versus euxinic conditions to the classic bulk Fe speciation and TOC/P redox proxies recorded in the Sedimentary Geochemistry and Paleoenvironments (SGP) phase 1 database (Fig. 5) (*24*, *25*).

The Precambrian record oscillates between pyrite types 1 and 3 (Fig. 4, A and B), which suggests precipitation in DOM microenvironments and/or where ambient waters were generally suboxic or anoxic and not euxinic or strongly oxygenated (Fig. 4C). This is consistent with weak oxygenation (*3*, *7*) and a large pool of labile, poorly ballasted OM (*66*). With respect to Mn-Fe-OM dynamics, dominance of types 1 and 2 pyrite before 3.1 Ga suggests that ambient conditions were nonsulfidic and hosted a mixture of dissolved Fe and Fe (oxyhydr)oxides. Between ca. 3.1 and 3.0 Ga, an expanded type 1 pyrite pool suggests that ambient conditions became strongly enriched in dissolved Fe, perhaps related to elevated biotic reduction of Fe (oxyhydr)oxides coupled to productivity. Between ca. 3 and 2.8 Ga, expanded type 2 pyrite suggests a reversal toward Fe (oxyhydr)oxide–enriched conditions (Fig. 4E), consistent with increasing water column oxygenation ahead of the GOE (*44*). From ca. 2.8 Ga to the end of the GOE at ca. 2.1 to 2.0 Ga (*45*), a broad peak in the abundance of type 1 pyrite suggests that dissolved Fe was the primary Fe reactant for pyrite precipitation (i.e., anoxic ambient conditions). Similar to ca. 3.1 to 3.0 Ga, this may indicate elevated rates of Fe (oxyhydr)oxide dissolution coupled to productivity and/or oxidation of H_2_S. While the pyrite record between ca. 3.0 and 2.0 Ga favors Fe-buffered anoxic conditions, the generally large pyrite fractional uncertainty does not preclude the existence of transient or localized strongly sulfidic conditions [see, e.g., (*45*–*46*)], consistent with the record of pyrite nodule–bearing rocks in this interval. Replacement of type 2 pyrite with types 1 and 3 pyrite during the GOE could also indicate faster pyrite reaction kinetics related to increased availability of sulfate.

Near the end of the GOE at ca. 2.1 Ga, a step change from type 1 to type 2 pyrite suggests that ambient marine conditions became more oxygenated and/or that pyrite kinetics became more sluggish. These conditions persisted between ca. 2.1 and 1.7 Ga, an interval that includes the Talvivaara deposit (*6*), an important highly enriched metalliferous black shale (HEBS). This deposit coincides with a small type 5 pyrite pool, suggesting that the mineralization was linked to oxygenation and/or productivity. Occurrences of sedimentary-exhalative (SEDEX) Pb-Zn deposits from ca. 1.9 Ga onward, not coincident with types 3 and 4 pyrite (Fig. 4), are consistent with a model that implicates diagenetic hydrothermal mineralization (*33*) rather than exhalative mineralization. Furthermore, onset of SEDEX Pb-Zn mineralization during a shift toward apparently Fe (oxyhydr)oxide–enriched conditions implicates mineralization linked to diagenetic mobilization of Pb and/or Zn absorbed onto Fe (oxyhydr)oxides.

At ca. 1.7 Ga, type 1 pyrite suggests that the Fe (oxyhydr)oxide pool was swiftly replaced by dissolved Fe, likely related to development of increasingly sulfidic conditions (Fig. 4E). Type 3 pyrite shows that euxinia sensu stricto also developed near or at the Paleoproterozoic-Mesoproterozoic boundary (ca. 1.7 to 1.6 Ga). Following a peak at ca. 1.5 Ga, ambient sulfidic conditions progressively contracted to the end of the Mesoproterozoic, consistent with modeling (*43*, *49*). During the Mesoproterozoic, Fe_py_/Fe_HR_ rarely exceeds 0.7, a key threshold for identifying euxinia (Fig. 5A) (*8*, *68*, *69*). Where Fe_py_/Fe_HR_ > 0.7, this suggests that euxinia developed during the late Mesoproterozoic (ca. 1.3 to 1.1 Ga) rather than the early Mesoproterozoic. The redox proxy TOC/P (*70*) suggests generally mixed redox conditions in this interval (Fig. 5B). The discrepancy between timing of euxinia inferred from the pyrite types and Fe speciation (Fig. 5C) is best explained by spatially transient conditions or potentially fallout of type 3 pyrite beneath suspended euxinia. At least, localized euxinia is broadly supported by evidence for localized mid-depth sulfide (*8*), termination of banded iron formations (BIFs), and continuing SEDEX mineralization (Fig. 4). The discrepancy between the onset of euxinia based on the pyrite types (ca. 1.7 to 1.6 Ga) versus ca. 2.20 to 1.84 Ga (*43*, *47*) may also simply relate to spatially variable redox conditions during the late Paleoproterozoic (*47*). In addition, the Rove Formation at 1.84 Ga (fig. S13) included in the pyrite dataset (*19*–*23*) and deposited under localized euxinic conditions (*47*) corresponds to a period of increased uncertainty in terms of the fraction of euxinic pyrite predicted by machine learning.

Between 1.5 and 1.4 Ga, type 2 pyrite suggests that Fe (oxyhydr)oxides possibly returned briefly as an important reactant during pyrite precipitation (Fig. 4E), suggesting a potential retraction in the spatial extent of euxinia and either more widespread ferruginous or oxic ambient conditions (*72*, *73*). From 1.4 Ga to the Precambrian-Phanerozoic boundary (ca. 541 Ma), type 1 pyrite suggests that ambient conditions drifted consistently toward enrichment in dissolved Fe and depletion in Fe (oxyhydr)oxides, suggesting more pervasive anoxia. Between 1.4 and 0.9 Ga, pyrite precipitated increasingly within microenvironments, consistent with independent evidence for ferruginous anoxia (*50*) and a sharp decline in Fe_py_/Fe_HR_ near ca. 1.1 Ga (Fig. 5A). From ca. 900 to 800 Ma, redox conditions were weakly Fe-buffered and drifted toward more sulfidic ambient conditions, a trend that is associated with a pronounced increase in the fraction of framboid-bearing rocks (Fig. 1). Overall, the 2-1-3 pyrite sequence (Figs. 3 and 4), which initiated at ca. 1.5 to 1.4 Ga, culminated in a peak in the fraction of type 3 pyrite near the end of the Tonian between ca. 800 and 740 Ma. This coincides with independent evidence for euxinia (*74*–*76*), including elevated TOC/P (Fig. 5B). The absence of pyrite nodule–bearing rocks (Fig. 1) and generally low to moderate Fe_py_/Fe_HR_ (Fig. 5A) could indicate development of transient, suspended (rather than bottom-up) euxinia, similar to the early Mesoproterozoic. Type 1 pyrite suggests that euxinia possibly slightly contracted during the middle Cryogenian (ca. 740 to 690 Ma) and then expanded between ca. 690 and 655 Ma. This pattern is consistent with evidence for at least localized euxinia following the Sturtian glaciation [see, e.g., (*77*)]. The pyrite record during the late Cryogenian to early Ediacaran (ca. 655 to 610 Ma) shows a return to dominantly Fe-buffered conditions, followed by increasingly sulfidic conditions from ca. 610 Ma to the early Cambrian (ca. 532 Ma). From the start of the Ediacaran, the pyrite and Fe_py_/Fe_HR_-derived redox fractions are coherent and gradually drift toward increasing euxinia (Fig. 5C). This suggests progressive initiation of bottom-up euxinia, a phenomenon potentially driven by an increased flux of OM to seafloor, which induced migration of the locus of sulfate reduction from the water column into sediments. By contrast, TOC/P shows a reversal toward apparently more ferruginous conditions (Fig. 5, B and C). This dichotomy is best explained by fixation of authigenic P under strongly euxinic and methanogenic conditions (*78*).

### A new perspective on the modes of anoxia in Phanerozoic time and space

**Fig. 6.**
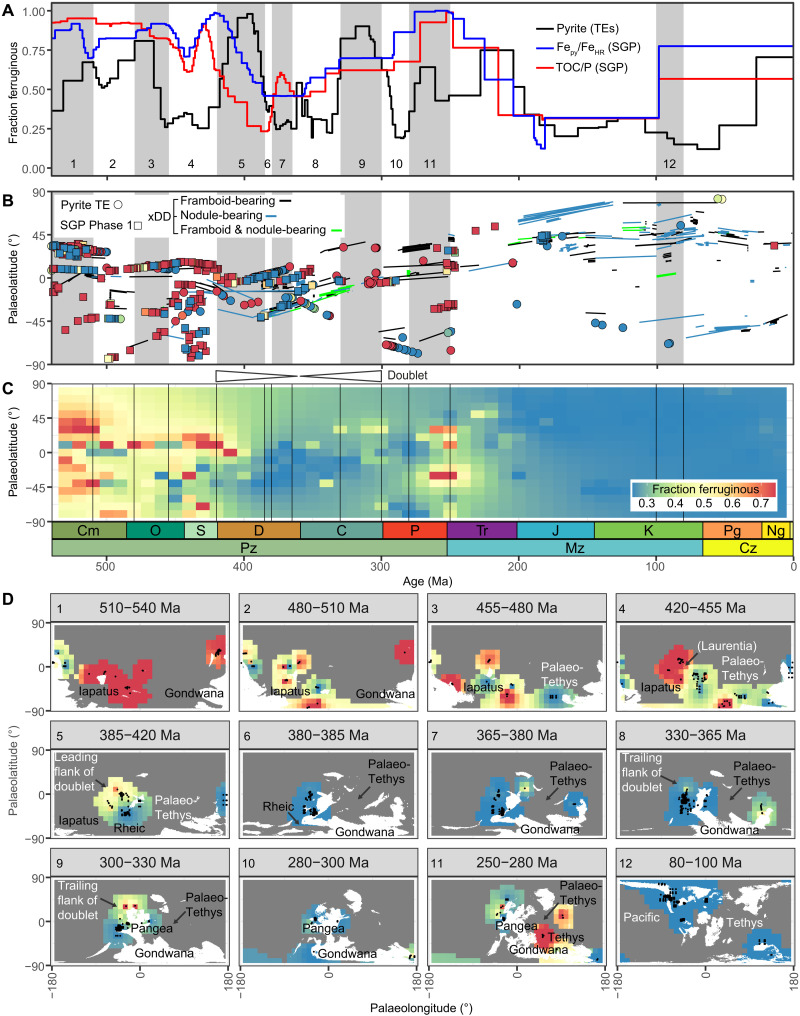
Modes of anoxia through Phanerozoic time and space based on a compilation of xDD pyrite text mining (*n* = 6885), pyrite TE interpretations (*n* = 196), and SGP phase 1 Fe speciation and TOC/P data (*n* = 443). (**A**) Fractions of euxinic versus ferruginous paleoredox conditions (as in Fig. 5C). (**B**) Paleolatitude reconstructions versus age derived from the GPlates PALEOMAP PaleoAtlas and web service (*79*) using the R package chronosphere (see the Supplementary Materials). (**C**) Weighted fractions of euxinic versus ferruginous conditions by paleolatitude and age. Values were interpolated on a 10° × 10 Ma grid via inverse distance weighting. (**D**) Fractions of euxinic versus ferruginous conditions for Paleozoic time slices and one Mesozoic time slice. Modes of anoxia were mapped to a 10° × 10° grid via inverse distance weighting (see Materials and Methods). Paleogeographic reconstructions are weighted to marine occurrences and derive from the PaleoDEM (*80*) series. The Paleozoic was selected for mapping because this era spans the transition from prevalence of ferruginous to euxinic conditions and is extensively documented in the SGP database. A time slice containing the Cretaceous Cenomanian–Turonian OAE is also shown for comparison. Interpolations extend 2500 km from each observation. Each time slice represents the minimum elevation recorded in each 5-Ma DEM to capture all possible submarine occurrences. A value of >100 m above sea level defines permanent subaerial conditions.

We explored Phanerozoic spatiotemporal redox variation by combination of the xDD text-mining outputs, *k*NN pyrite–type predictions, and the bulk redox proxies (Fig. 6) (*24*, *25*). Spatial analysis shows that the xDD, pyrite trace element, and SGP datasets are biased toward the flanks of Laurentia and Pangaea (Fig. 6, B to D). The spatiotemporal interpolation of ferruginous versus euxinic conditions (Fig. 6, C and D) is based on two key assumptions: (i) the record of pyrite nodule–bearing rocks reflects development of at least intermittently euxinic conditions, and (ii) the pyrite types are primarily driven by Mn-Fe-OM dynamics.

The late Ediacaran (ca. 580 Ma onward) and Ediacaran-Cambrian boundary (ca. 541 Ma) exhibit stepwise increases in the fraction of type 5 pyrite (Fig. 4, A to C), potentially signaling the development of POM microenvironments related to the evolution of complex life (*3*). The appearances of ballasted POM substrates are consistent with a generally increased capacity for export of OM to the seafloor, a phenomenon that may explain generally elevated TOC in Phanerozoic shales (*81*). The explosion of pyrite types 4 and 5 at the Ediacaran-Cambrian boundary, coincident with increased atmospheric oxygenation, could also implicate a fundamental change in the behavior and availability of metals in ambient seawater [sensu (*22*, *48*)]. The Cambrian consistently shows a high ratio of pyrite types 5 and 2 versus type 1 pyrite (Fig. 4E), possibly related to abundant Fe (oxyhydr)oxide substrates and/or increased oxygenation (*48*).

The pyrite record during the early Cambrian, spanning ca. 541 to the Botomian crisis at ca. 514 Ma (*82*), suggests generally productive (POM) and Fe-buffered conditions, punctuated by short-lived weakly euxinic intervals. This is consistent with a high abundance of framboid-bearing rocks and a lack of pyrite nodule–bearing rocks (Fig. 1), as well as very low Fe_py_/Fe_HR_ and low TOC/P ratios (Fig. 5, A and B). In a departure from the Ediacaran, the earliest Cambrian Fe speciation and TOC/P-derived redox fractions exhibit an inverse relationship with the pyrite-derived redox fractions (Fig. 5). This suggests (i) a short-lived return to sulfate reduction in the water column, leading to development of suspended euxinia, at least locally along the northeast margin of Gondwana (Fig. 6D), followed by (ii) anoxic systems, which became increasingly POM-ballasted from the early Cambrian to the Botomian crisis, a process that pushed the locus of sulfate reduction into the sediments (Fig. 5C).

Following the Botomian crisis, type 2 pyrite suggests expanded DOM microenvironments, reduced oxygenation, and/or generally sluggish pyrite reaction kinetics through the middle to late Cambrian (Fig. 4). This trend stabilized in the early Ordovician (ca. 480 Ma), coincident with a fundamental shift in the structure of the marine biosphere during the Great Ordovician Biodiversification Event (*83*). Pyrite precipitation within microenvironments from the middle Cambrian to early Ordovician is supported by low Fe_py_/Fe_HR_ and TOC/P (Fig. 5) and independent evidence for ferruginous deep waters (*84*) and basins (*85*). Ferruginous conditions were apparently punctuated by spatially and temporally transient weakly euxinic conditions (Figs. 5 and 6). Between ca. 480 and 456 Ma, rapid expansion of type 4 pyrite (Fig. 4), followed by type 3 pyrite, suggests a return to euxinia sensu stricto. The transition to euxinic conditions derives solely from present-day Australia (Tasmania; Fig. 4F and fig. S23) and thus may represent a local signal on the margins of Gondwana (Fig. 6D). Similar to the early Cambrian, reflection of the pyrite and bulk redox proxies suggests that the locus of sulfate reduction, and by extension euxinia, was primarily suspended in the water column (Fig. 5C). This interval is associated with a continued decline in type 5 pyrite following a plateau in the early Ordovician, a gradual drift from type 2 to type 1 pyrite, and appearance of pyrite nodule–bearing rocks (Fig. 1). The lagging drift toward type 1 pyrite suggests protracted Fe (oxyhydr)oxide dissolution coupled to euxinia, which persisted from ca. 456 Ma to the end Ordovician mass extinction (ca. 444 Ma) to the end Silurian (ca. 419 Ma). Similar to the Botomian crisis, the end Ordovician Fe_py_/Fe_HR_ and TOC/P redox proxies mirror the pyrite-based record of euxinia (Fig. 5C). This suggests that the end Ordovician crisis was associated with replacement of DOM by POM and, by extension, temporary migration of the locus of sulfate reduction from the water column into sediments. Increasingly euxinic conditions near the end Ordovician mass extinction apparently developed on the margins of Laurentia and Gondwana (Fig. 6).

A low proportion of pyrite framboids or nodules delineated in the Ordovician text-mining output despite evidence for euxinia is best explained by localized or suspended free H_2_S surrounded by ferruginous conditions (*85*). Glaciation during the end Ordovician mass extinction potentially further suppressed the text-mining signal, and the signal in the geological record itself, by pushing euxinia beyond most continental shelves and intracontinental basins in the focal area (*86*). A low proportion of Wilkin-pyrite mentions also suggest that this part of the geological record has received relatively little attention compared to the younger major crises.

The Devonian and Carboniferous (ca. 419 to 299 Ma) pyrite fractions and text-mining outputs are reflected on an axis at ca. 367 Ma, between the Frasnian-Famennian and Hangenberg late Devonian crises (Fig. 4). The leading sequence encompasses (i) ubiquity of type 5 pyrite from 419 Ma to immediately below the Givetian-Frasnian extinction event at ca. 383 Ma (*34*, *35*); (ii) a sharp increase in the fractions of type 4 pyrite and pyrite nodule– and framboid-bearing rocks, at or close to the Givetian-Frasnian event (ca. 383 Ma); (iii) expansion of type 3 and type 1 pyrite coupled to a slight decline in the fraction of nodule- and framboid-bearing rocks, culminating in the Frasnian-Famennian extinction event at ca. 372 Ma (*35*); and (iv) continued Fe (oxyhydr)oxide decline to ca. 367 Ma, lagging euxinia similar to the late Ordovician. The 5-4-3 pyrite sequence is repeated in reverse from ca. 367 Ma to the end of the Carboniferous (ca. 299 Ma) (Fig. 4). This is best explained in terms of the position of a generalized ferruginous-euxinic chemocline, perhaps near the top of one or more thick oxygen minimum zones (*87*). Chemocline shoaling and retreat initiated immediately before the Givetian-Frasnian crisis and at ca. 367 Ma, respectively. Thus, intervals dominated by candidate POM microenvironments (ca. 419 to 383 Ma and 330 to 299 Ma) and the incipient euxinic Givetian-Frasnian and Hangenberg (ca. 359 Ma) crises are part of a doublet causally linked to chemocline systematics (Fig. 4E). This pattern is consistent with only the Frasnian-Famennian crisis being linked to spatially extensive and fully fledged euxinia (e.g., type 3 pyrite; Figs. 4 to 6) (*2*, *87*). Disappearance of type 5 pyrite and emergence of type 1 pyrite in the midst of the crisis does not imply that the system was not POM-driven but favors decoupling of the loci of syngenetic pyrite precipitation and POM degradation under highly productive conditions.

The doublet generally reflects conditions along the north-west flank of Laurentia (Fig. 6D) because the pyrite trace element data generally are derived from a small number of high-resolution studies from the Great Basin (United States) and Richardson Trough (Canada) (Fig. 4F), where basins were likely relatively productive and weakly restricted. A similar bias toward a small number of high-resolution studies is also observed in the SGP database (Fig. 6B). The sampling resolution is less advanced in other regions, but the spatial analysis suggests that (i) the doublet was not likely a global or synchronous phenomenon and that (ii) both the Panthalassic and Paleo-Tethys oceans hosted euxinia in the late Devonian (Fig. 6D). This is evidenced by a shift toward euxinic conditions recorded in pyrite trace element analyses on the flanks of Laurentia (Richardson Trough) and Gondwana (Canning Basin, Australia) (Fig. 4F and fig. S23).

Similar to the pyrite types, Fe_py_/Fe_HR_ and TOC/P-based redox fractions are also inflected near ca. 367 Ma but show some key differences (Figs. 5 and 6). First, Sperling *et al*. (*85*) independently recognized the rising limb of the doublet through the Road River Group included in the SGP phase 1 dataset. Fe_py_/Fe_HR_ and TOC/P are generally coherent, but TOC/P precedes Fe_py_/Fe_HR_. Similar to the Neoproterozoic, this pattern is consistent with the TOC/P proxy being more sensitive to weakly sulfidic early diagenetic conditions that mobilized P but did not induce substantial pyrite precipitation. Both Fe_py_/Fe_HR_ and TOC/P show the development of increasingly euxinic conditions before the pyrite-type record (Fig. 5). This may reflect accumulation of early diagenetic sulfide overlying generally ferruginous bottom water conditions on the flank of the doublet. In the center of the doublet, TOC/P reversal toward apparently less euxinic conditions is best explained by authigenic P trapping under strongly euxinic and methanogenic conditions, similar to the Ediacaran.

The link to global crises suggests that the pyrite doublet, which operated at least along the margin of Laurentia, is best explained by a combination of environmental drivers coupled to basin configuration. In this respect, chemocline systematics explain the temporal distribution of Paleozoic HEBS (*6*), which are consistently dominated by Fe (oxyhydr)oxide pyrite substrates and primarily occur during periods of chemocline shoaling or retreat. Most Devonian-Carboniferous HEBSs occur on the flanks of the doublet dominated by type 1 pyrite. This is consistent with HEBS genesis in ocean-facing, suboxic or anoxic, nutrient and oxide-rich, productive, weakly restricted but not strongly euxinic settings (*5*, *6*) and implicates mineralization under non–steady-state early diagenetic conditions. By contrast, coincidence of types 3 and 4 pyrite, pyrite nodule–bearing rocks, and most SEDEX and Mississippi Valley-type (MVT) deposits during the midst of the crisis (Figs. 1 and 4) is consistent with mineralization associated with elevated syngenetic and early diagenetic sulfide and/or methane production, respectively.

Pyrite types 1 to 3 expanded during the early-middle Permian (ca. 299 to 283 Ma; Figs. 4 to 6), a signal that derives primarily from one high-resolution study on the flank of Gondwana (Tasmania Basin) (Figs. 4F and 6, B to D). Thus, divergence between the pyrite and Fe_py_/Fe_HR_ and TOC/P fractions is potentially due to localized redox conditions or reduced temporal resolution in the SGP database. Following this interval, the proportion of pyrite types 1, 2, and 5 broadly increases from the middle Permian (ca. 283 Ma) to the end Triassic (ca. 201 Ma). This suggests that anoxic conditions were generally Fe-buffered, supported by occurrences of hematite nodules from at least the middle Permian onward (*88*), the absence of pyrite nodules (Fig. 1), and moderate Fe_py_/Fe_HR_ and TOC/P (Fig. 5). The end-Permian mass extinction is linked to expanded ferruginous anoxia and euxinia [e.g., (*89*)], corresponding to a peak in the abundance of framboid-bearing rocks (Fig. 1), and a possible shift toward DOM microenvironments and potentially more variable Fe systematics (Fig. 4). The Paleo-Tethys Ocean apparently hosted more protracted ferruginous conditions compared to relatively sulfidic basins facing the Panthalassic Ocean (Fig. 6, B to D).

The Mesozoic includes the end-Triassic mass extinction and the widely recognized ocean anoxic events (OAEs) (Figs. 1 and 4), crisis defined by the expansion of dense, warm, euxinic, and acidic waters (*2*). Euxinia from the end Triassic onward is consistent with the ubiquity of pyrite types 3 and 4 (Figs. 4 and 5C). Generally, the SGP datasets offer reduced temporal resolution through the Mesozoic, but generally high Fe_py_/Fe_HR_ and TOC/P also suggest prevalence of euxinia (Figs. 5 and 6, B to D). Pervasive occurrences of pyrite nodules (Fig. 1) likely reflect the combined effects of permanent migration of sulfate reduction into sediments (*90*) linked to ballasting by the major phytoplankton groups (*91*) and an increased seawater sulfate concentration (*92*). Migration of the locus of sulfate reduction permanently into sediments is consistent with coherency between the redox proxies (Fig. 6A). Peaks in type 4 pyrite and the fraction of nodule-bearing rocks suggest that the Toarcian-Pliensbachian OAE was associated with extensive weakly or incipient euxinic conditions comparable to the Givetian-Frasnian crisis. Mesozoic black shales generally lack evidence for POM and Fe (oxyhydr)oxide substrates as an explanation for the paucity of HEBS, with the exception of the Hatteras Formation during Cretaceous OAE2 (*6*). Widely anoxic conditions associated with the end Devonian, end Permian, and end Triassic mass extinctions contrasts with the Cretaceous-Paleogene bolide impact and mass extinction, which is not associated with a peak in the text-mining outputs, consistent with a lack of evidence for extensive anoxia (*93*). Since the evolution of the modern phytoplankton groups, Fe-buffered and euxinic systems were likely synchronous but spatially moderated by plate tectonics. Thus, retreat of major epicontinental Mesozoic seaways best explains the drift toward pyrite nodule deficiency and expansion of type 5 pyrite in the Cenozoic (Figs. 1 and 4).

## DISCUSSION

Text mining coupled to multivariate statistical analysis of pyrite compositions reveals a complexity in marine anoxia and the origin of sedimentary pyrite through geological time that has been difficult to appreciate. Five types of syngenetic pyrite existed in the ancient marine environment. Unique combinations of pyrite framboid– and nodule-bearing rocks, pyrite compositions, and bulk redox proxies offer new perspectives on the modes of anoxia through Earth’s history. Multivariate analysis shows that element contents in pyrite are clustered most clearly in terms of substrate (or lack thereof) and element compatibility and less clearly in terms of concentrations or residence times in modern seawater (Fig. 2). Thus, element quotas in pyrite may not directly proxy for any single property of the biosphere, but the composition of syngenetic pyrite is nonetheless dependent on multiple, potentially coupled, environmental factors including nutrient and oxygen availability, deepwater circulation and basin configuration, and element mobility. This explains why paleoatmosphere oxygen concentrations derived from pyrite chemistry (particularly Se/Co) are often, but not always, consistent with independent proxies for oxygen in the atmosphere [see, e.g., (*22*)]. Substrate ubiquity and dependency explain why syngenetic pyrite trace element quotas may vary widely within single shale units and why the same pyrite type is found in rocks of very different ages (e.g., the same pyrite types occur in Archaean shales and modern sediments). Assuming operation of Mn-Fe-OM dynamics, type 3 pyrite shows the most potential as a true sample of ambient seawater, but this signal is likely to be prone to reservoir effects. Intraunit pyrite trends, generally at 0.1- to 10-Ma time scales, may indicate shoaling or sinking of chemoclines bounding suboxic-ferruginous-euxinic waters and are not necessarily indicative of high-frequency perturbations in atmospheric oxygen. By extension, the substrate control on pyrite trace element chemistry shows that far-field responses to atmospheric oxygenation cannot be assumed a priori.

This pyrite mega-analysis suggests—with some key assumptions—that seven time intervals hosted protracted, consecutive, or closely spaced periods of euxinia. The Mesoproterozoic (ca. 1.7 to 1.4 Ga or 1.7 to 1.1 Ga), the late Tonian-Cryogenian (ca. 800 to 655 Ma), late Ediacaran–middle Cambrian (ca. 575 to 514 Ma), middle Cambrian–late Silurian (ca. 514 to 419 Ma), and middle Permian (ca. 299 to 283 Ma) accommodated at least localized euxinia. Crises during the late Devonian–early Carboniferous (383 to 344 Ma), from the Triassic onward (including OAEs) and possibly near the end Ordovician (ca. 444 Ma), were widely and strongly euxinic. Fe (oxyhydr)oxide–decoupled euxinia implies a substantial eutrophication P pump during these crises. By contrast, the Botomian (ca. 514 Ma) and end-Permian crises (ca. 251 Ma) are linked to expansion of generally ferruginous anoxic conditions, where pyrite precipitated primarily within microenvironments. Decoupling between the classical and pyrite trace element–based paleoredox proxies during the Mesoproterozoic, late Tonian-Cryogenian, earliest Cambrian, middle Ordovician–late Silurian, and possibly the early Permian–late Triassic potentially implicates euxinia suspended in the water column. Coupled Fe speciation and pyrite trace element analysis for the same sample suites could resolve this discrepancy. Critically, text mining coupled to multivariate analysis suggests that pyrite morphology and chemistry are proxies for a wider range of ambient redox conditions than previously recognized, from oxide-rich anoxic to strongly sulfidic settings. This link between pyrite chemistry and local conditions offers new opportunities for resolving the controls on anoxia (productivity and restriction), element and nutrient cycling, and the genesis of ancient marine mineral deposits.

## MATERIALS AND METHODS

### Text mining (xDD)

Text mining was implemented using the xDD digital library and machine-reading system (*26*) coupled to an algorithm that decomposes sentences into speech and linguistic components (fig. S1) using the Stanford NLP (*27*). The algorithm was written primarily in Python (https://github.com/jemmings-git/pyrite_app) and matched phrases extracted from the xDD library (10,661,918 documents at the time of analysis) to stratigraphic names recorded in the Macrostrat stratigraphic database (“tuples”) (*28*–*30*). A total of 9043 documents in the xDD library contain 31,873 mentions of “pyrite.” The dataset was subdivided using the qualifying adjectives “framboidal” or “nodular/concretionary” and common semantic variants such as “nodule.” Following deployment of the pyrite text-mining application, manipulation and analysis of the text-mining outputs underpinning Fig. 1 were coded in R (https://github.com/jemmings-git/pyrite_analysis). The raw xDD extracted phases comprise ca. ±100 words surrounding each tuple (see the Supplementary Materials for examples). Since the original publications are copyrighted and cannot be distributed publicly en masse, we condensed the raw phrases into the tuples and contextual phrases of interest. The code is fully deployable, and the results are fully reproducible using this public-facing output. The Supplementary Materials includes a list of the required R packages and a guide to the public-facing xDD pyrite results. Repeat matches between target phrases and stratigraphic names were omitted. Manual assessment (see the Supplementary Materials) indicated an effective accuracy between 87% (mentions of Precambrian framboids) and 95% (taking a random 5% sample of all mentions of framboids, nodules, and concretions). The measured accuracy (87 to 95%) is consistent with a previous estimate of at least 90% for a similar approach (*31*). Erroneous results most commonly relate to false stratigraphic matches and very rarely include negative assertions (i.e., “does not contain framboids”; see the Supplementary Materials). The stratigraphic database used is a composite of metasedimentary and sedimentary “Macrostrat units” (*28*) present in the Macrostrat focal area (North America, the Caribbean, New Zealand, the deep sea, and parts of Central and South America, including data from the U.S. Geological Survey and Natural Resources Canada) and metasedimentary and sedimentary lithostratigraphic packages recorded in Geoscience Australia and British Geological Survey (BGS) stratigraphic lexicons. This composite approach amplifies stratigraphic matches for rocks present in the Macrostrat focal area (fig. S2), benefitting from improved temporal and lithological resolution (figs. S4 and S5). A drawback of this approach is that the composite record is biased; only ca. 13% of packages and units are derived from outside the Macrostrat focal area (fig. S6). Therefore, the analysis was also conducted without propagation of Macrostrat units (figs. S2 and S3), where ca. 36% of packages were derived from outside the Macrostrat focal area. This approach yielded a similar result (Fig. 1). Correlations to chronostratigraphic intervals and consideration of contact relationships are used to model top and base ages for each Macrostrat unit (figs. S8 to S10) (*94*).

All stratigraphic data (including from Geoscience Australia and BGS) were accessed from Macrostrat via the system’s application programming interface (API) (*32*). The extracted xDD record was supplemented with mentions of “pyrite,” including framboidal or nodular/concretionary variants, recorded within Macrostrat, primarily within the Geoscience Australia and BGS lithological descriptions. The framboid-bearing rock record derives from slightly more relaxed rules compared to the nodule-bearing rock record because we searched for mentions of framboids within the phrases surrounding undifferentiated pyrite-stratigraphic tuples (in addition to the direct framboid-stratigraphic tuples). The extracted pyrite record represents a sample because the number of matched pyrite framboid and nodule-bearing packages (140 and 110) is almost certainly smaller than the number of pyrite framboid– or nodule-bearing rocks actually present in the focal area. The record is therefore interpreted in terms of relative proportions. The relatively low number of framboid- and nodule-bearing packages is best explained by our relatively strict assessment of tuples using NLP, designed to minimize false positives.

We sample a large library that is unlikely to be strongly biased in the focal area with respect to the temporal distribution of pyrite framboids or nodules. The xDD pyrite records are potentially biased depending on the type and extent of sediments present in the Macrostrat focal area, the presence of important extinction events, economic deposits, and other intervals of historic interest. This type of bias should equally affect the framboid and nodule signals. Thus, our approach is to interpret the text-mining outputs primarily in terms of the relative proportions of framboids and nodules, rather than in absolute terms.

In specific cases where the xDD pyrite record is interpreted in absolute terms, we use an independent approach designed to measure bias. We searched the xDD snippets API (see the Supplementary Materials) for mentions of “Wilkin,” pyrite, and preindexed stratigraphic names derived from the Macrostrat stratigraphic database (inclusive) (Fig. 1C). The Wilkin-pyrite approach targets the transformative papers detailing the framboid size proxy, authored or coauthored by J. Wilkin (*38*–*40*). The xDD snippets search returned 1210 documents, a result that is similar to the number of citations in Google Scholar [references (*38*–*40*) have been collectively cited 1973 times, 922 + 332 + 719]. The whole publication approach cannot contain false negatives but plausibly contains false positives. For a conservative approach, we limited the stratigraphic packages to those described as (meta)sedimentary rocks. The number of unique stratigraphic packages mentioned in each publication was counted. In publications that mention only one stratigraphic package (190 documents), the Wilkin-pyrite record is best interpreted as a crude alternative measure for the proportion of pyrite-bearing rocks. At increasing numbers of stratigraphic packages mentioned in each publication (364 documents mention one to three packages, 451 documents mention one to five packages), we interpret the Wilkin-pyrite results as a measure for the level of attention each part of the stratigraphic column has received in terms of framboidal pyrite and/or paleoredox research. Where publications mention more than five unique stratigraphic packages, interpretations become increasingly uncertain and likely include book chapters, etc. Removal of any limit to the number of unique stratigraphic packages in Wilkin-pyrite publications generates an output that resembles the distribution of undifferentiated pyrite mentions (figs. S4 and S5).

We focus on the changing relative proportions of framboid-bearing versus nodule-bearing rocks, observations that are equivalent in terms of sophistication (i.e., unambiguous and historically consistent observations at scales of hand specimen and optical microscopy); thus, artifacts due to sampling bias are likely negligible. Lithology fractions (fig. S6, C and D) are estimates on the basis of manual assessment and machine (grepl) searches of lithology strings contained in the Macrostrat unit lithology and concept fields. The proportion of mineralization (including veins) (fig. S7D) derives from mentions of veins or mineralization within the extracted xDD pyrite phrases and Macrostrat lithology, environment, and concept description fields. The proportion of evaporitic (meta)sedimentary rocks (fig. S7E) derives from mentions of “evaporite” and “evaporitic” in the same fields as described for mineralization (above). Thus, the fractions of mineralized and evaporitic (meta)sedimentary rocks are first-order contextual estimates based on Boolean logic (presence or absence), are not weighted for intrapackage extent (i.e. coherent with the record of framboid- and nodule-bearing rocks), and may include false positives. Figures 1 and 5 include the ages of SEDEX Pb-Zn mineral deposits (*4*), selected HEBS (*6*), BIFs (*95*, *96*), and the major glaciations (*97*–*101*).

### Pyrite elemental and bulk geochemical datasets

Import, manipulation, and analysis of pyrite trace elements were conducted in R (https://github.com/jemmings-git/pyrite_analysis). We used two open-access datasets. First, we used Fe speciation and TOC/P data extracted from phase 1 of the SGP Project (http://sgp-search.io/) (*24*) (see the Supplementary Materials for the API call). A list of references underlying the SGP dataset is provided in the accompanying repository. Second, we used a syngenetic to early diagenetic pyrite trace element dataset (*19*–*23*) (https://doi.org/10.1130/GEOL.S.12456332). To optimize temporal resolution, we did not cull either dataset to the Macrostrat focal area. We also supplemented the SGP TOC/P data with an alternative TOC/P dataset (*71*), culled for anoxic shales (where *V* content is >300 parts per million), and culled for samples not already present in the SGP dataset.

We replaced values below detection limit and rounded zeros (i.e., left-censored data) with imputed values (*102*–*104*). Nonparametric multiplicative simple imputation was conducted to maximize the data coverage and ensure that the dataset was as representative as possible. We then conducted the following HCA: (i) sample-wise Ward’s sum of squares of the Euclidian distance matrix for log ratio (*clr*)–transformed (*55*, *104*) trace element concentrations and (ii) element-wise clustering by Ward’s sum of squares of the Euclidian distance matrix for the element variation array (*104*). *clr* transformation was adopted because raw concentrations are susceptible to closed data effects (*105*). Clusters (pyrite types) were determined by cutting the HCA trees at the base of the long branches without agglomeration. The HCA biplot (Fig. 2A) includes the *clr* matrix with a standardized color scale (to aid visualization) and includes element residence times and concentrations from (*106*) and the Monterey Bay Aquarium Research Institute periodic table of elements in the ocean [www.mbari.org/science/upper-ocean-systems/chemical-sensor-group/periodic-table-of-elements-in-the-ocean/; accessed 2021] and references therein. Tl ocean concentration and residence time data are from (*107*). Principal components analysis (PCA) was conducted using the covariance matrix of *clr*-transformed (Fig. 2B) element concentrations.

We assume that the pyrite analyses reported by Large *et al*. (*19*–*23*) are (i) syngenetic or plausibly very early diagenetic (i.e., formation within centimeters of seabed, under fully open-system conditions), and (ii) a single “pyrite” analysis may contain multiple metal-bearing phases, such as nanometer-sized organic and mineral inclusions (*108*), but that all these phases are also of a syngenetic origin that is shared with the primary or precursor (e.g., FeS) sulfide phase, also assumed previously. The pyrite analyses are considered syngenetic or early diagenetic based on the textural and chemical screening conducted previously (*19*–*23*). Briefly, the pyrite dataset only contains laser ablation inductively coupled plasma mass spectrometry analyses that (i) derive from rocks below mid-greenschist metamorphic grade; (ii) target framboids, microcrysts, and other syngenetic or early diagenetic pyrite textures; and (iii) lack evidence for late diagenetic or metamorphic mineralization such as zoned pyrite, euhedral overgrowths, pyrrhotite, or extreme metal concentrations. Each analysis is also corrected for matrix effects.

### Predictions of pyrite types through geological time

Pyrite types (Fig. 4) were interpolated using the following method. First, we extracted the first seven PCs based on assessment of the scree plot (Fig. 2B, inset). PCA was implemented as both an exploratory tool and a dimensionality reduction technique ahead of machine learning. PCs 1 to 7 account for 77% of the total variation of the dataset. The PCA scree plot shows a large elbow at PC3 and a smaller elbow at PC7. PC7 was selected as an appropriate level of complexity for machine learning. Beyond PC7, the proportion of variance described by each successive PC is increasingly small. Next, the pyrite observations were partitioned for training (80%) and validation (20%). Several supervised machine learning algorithms were tested to predict pyrite types (i.e., hierarchical clusters by sample) using PCs 1 to 7 (fig. S11). The *k*NN algorithm was selected on the basis of superior accuracy [*k* = 5, 91% (88 to 92%) at 0.95 significance level; see the Supplementary Materials and fig. S11].

Next, we designed and implemented a method to interpolate PCs 1 to 7 through geological time. Local polynomial regressions (LOESS, at 1 polynomial degree) were computed for each PC. LOESS smooths were computed for the mean function and the means ± *k* multiplied by the SD (σ) function (up to a maximum of ±2σ). This provides a first-order estimate of the moving prediction interval for each PC through time (not equivalent to confidence intervals). The LOESS smooths were bootstrap resampled. The number of resamples per 0.2 σ increment was determined from a Gaussian distribution (*n* = 1000) around the mean function. Experimentation showed that it was not necessary to increase the number of resamples above 1000. This method produces a LOESS smooth population distributed around the mean LOESS and bounded by ±2σ. LOESS curves were computed independently for the Precambrian and Phanerozoic on the basis of the step change in temporal resolution at or near the Precambrian-Phanerozoic boundary. Prediction intervals and extrapolated fractions of ferruginous conditions for the SGP Fe_py_/Fe_HR_ and SGP TOC/P datasets (Fig. 5) were also calculated using bootstrapped LOESS regressions (as above, nboot = 1000). The LOESS hyperparameter *span*, which controls the extent of smoothing, was generally determined automatically and cross-validated (see the Supplementary Materials).

PC scores for each LOESS regression were extracted at 10- and 1-Ma intervals for the Precambrian and Phanerozoic, respectively. The interpolated PC scores were shuffled (by each 10- or 1-Ma increment), combined, and fed to the trained *k*NN algorithm. We repeated the shuffle, combine, and deploy step (*n* = 100) to estimate the mean and 2σ confidence intervals for the fraction of each pyrite type. Last, the results were down-sampled using the temporal resolution, resulting in a conservative measure for the fractions of the five pyrite types through time (figs. S15 to S19). Ratio uncertainties were propagated in quadrature. The bootstrap computation (*n* = 1000) requires ca. 1 hour to complete using a general purpose PC (i7-8650U CPU at 1.9 GHz, 2.11-GHz processor, 16-gigabyte RAM, 64-bit OS). Preliminary outputs can be generated swiftly by increasing the “speed” parameter. The *k*NN algorithm (*n* = 100) is deployed in ca. 1 hour and 15 min.

### Phanerozoic spatial and temporal interpolation of ferruginous versus euxinic conditions

We generated a composite of xDD-, pyrite trace element–, and bulk geochemical–based proxies for ferruginous versus euxinic anoxic conditions using the following method. First, we extracted the top, base and average ages, and present-day geographic coordinates (in decimal degrees) for each Macrostrat package or unit containing pyrite nodules (*n* = 6885). Geographic coordinates were added manually for stratigraphic packages outside the Macrostrat focal area by referring to the type section coordinates recorded in the BGS and Geoscience Australia stratigraphic lexicons. The pyrite nodule–bearing rocks were assigned a fixed ferruginous fraction of 0.25 based on the interpretation of at least intermittently free sulfide advection or stagnation in early diagenetic pore waters. Next, we manually assigned present-day geographic coordinates to each unique location or basin in the pyrite trace element dataset (*19*–*23*). We searched for the place names or basin names, recorded in the pyrite dataset, using Google Earth to obtain a first-order approximation of geographic coordinates. This approach is considered sufficient for the resolution of mapping implemented in this study but is not suitable for mapping at large scale. The ferruginous fraction derived from pyrite compositions was determined using the ratio of pyrite types (5 + 2 + 1)/(1 + 2 + 3 + 4 + 5) for each sample, aggregated for each unique age-coordinate combination. Last, SGP analyses (*24*) were designated as “ferruginous” or “euxinic” based on the Fe_py_/Fe_HR_ threshold of 0.7 (*8*, *68*, *69*) following filtering of samples that exhibit total Fe (Fe_T_) > 0.5 weight % (wt %) (*67*) and Fe_HR_/Fe_T_ > 0.38. The SGP ferruginous fraction was determined by aggregation of each unique age-coordinate pair similar to the pyrite trace element dataset.

Next, geographic coordinates were rounded to one decimal place, and ages were rounded to the nearest 5 Ma to compute reconstructed paleogeographic coordinates via the GPlates web service. Paleogeographic reconstructions derive from the Paleodigital elevation model (PaleoDEM) (*80*) series. Each time slice in Fig. 6 was flattened to the minimum elevation recorded in each DEM, available from the GPlates web service in 5-Ma increments. Flattening was conducted to capture all possible submarine occurrences. A value of >100 m above sea level was selected as a threshold for permanent subaerial conditions. Thus, the maps in Fig. 6 are not true paleogeographic reconstructions and are heavily biased toward the submarine extent. All reconstructions were derived from the GPlates PALEOMAP PaleoAtlas and web service (*79*) using the R package chronosphere (see the Supplementary Materials).

The composite xDD, pyrite trace element, and bulk redox datasets were resampled using the inverse of the squared spatial and temporal proximities (see the Supplementary Materials) (*25*). Resampling was implemented with replacement (*n* = 1,000,000) and where scale_age_ = 10 Ma and scale_spatial_ = 0.5, the same parameters as adopted by Mehra *et al*. (*25*). Resampling was considered particularly important to account for the spatially clustered nature of the dataset. Last, proportions of ferruginous versus euxinic conditions were interpolated through time and space via inverse distance (squared) weighting. The interpolations are designed to help visualize the first-order spatial patterns and do not account for factors such as basin geometries, upwelling, restriction, and ocean-atmosphere circulation. The GPlates algorithm is deployed in ca. 20 min.

## Supplementary Material

20220316-1

## Notes

**Correction (13 May 2022):** Due to a production error in a previous version, Figures 2 and 6 have been updated. The X-axis label in Figure 2B is horizontally plotted and a typo in the title of Figure 6D is corrected to “480–510 Ma.” The figures have been corrected in the updated online and PDF versions. These errors do not affect any of the results or conclusions in the article.
