# Supplementary material for: Pyrite mega-analysis reveals modes of anoxia through geological time
Source: Sci Adv. 2022 Mar 16;8(11):eabj5687. doi: 10.1126/sciadv.abj5687 (PMC8926349; doi:10.1126/sciadv.abj5687)
Supplement: Supplementary file 2 — Supplementary Text Tables S1 to S4 Figs. S1 to S24 [file sciadv.abj5687_sm.pdf]

Science Advances
AAAS

Supplementary Materials for

Pyrite mega-analysis reveals modes of anoxia through geological time

Joseph F. Emmings*, Simon W. Poulton, Joanna Walsh, Kathryn A. Leeming, Ian Ross, Shanan E. Peters

*Corresponding author. Email: Joseph.Emmings@cgg.com

Published 16 March 2022, *Sci. Adv.* **8**, eabj5687 (2022)
DOI: 10.1126/sciadv.abj5687

This PDF file includes:

Supplementary Text
Tables S1 to S4
Figs. S1 to S24

Supplementary Text

R packages

All data processing was coded in R (109) within RStudio (https://github.com/jemmings-git/pyrite_analysis). We used the following R packages (ordered alphabetically, with a brief description of purpose, not including any dependencies):

BBmisc (110) – standardization
boot (111, 112) - bootstrap
caret (113) – machine learning
chronosphere (114) – palaeogeographic reconstructions linked to the GPlates Web Server
colorspace (115) – colour assignment to heatmap objects
compositions (116) – clr transformation and variation matrices
data.table (117) – joining dataframes by keys
deeptime (118) – plotting chronostratigraphic scale bars
devtools (119) – required for installation of pammtools and safejoin
dplyr (120) – general data manipulation and aggregation
fANCOVA (121) – loess span determination by generalized cross validation (CV)
geoR (122) – spatial mapping functions (2D jittering)
geosphere (123) – spatial buffering around point data
ggbiplot (124) – PCA biplot (Fig. 3B)
ggfittext (125) – additional functionality for chronostratigraphic scale bars
ggnewscale (126) – additional colour/fill scales for ggplot objects
ggplot2 (127) – used to construct figures
gridExtra (128) – arrangement of ggplot2 objects
gstat (129, 130) – spatial mapping functions (inverse distance weighting)
httr (131) – data import
jsonlite (132) – data import
msir (133) - nonparametric estimation of mean loess with variability (σ)
pammtools (134) ¬ – ribbon layers for ggplot2 objects
pheatmap (135) – HCA visualization
randomForest (136) – for experimentation with random forest machine learning
RColorBrewer (137) – colour assignment to objects
readr (138) – data import
reshape2 (139) – data manipulation
rgdal (140) – spatial mapping functions
rio (141) – data import
safejoin (142) – data manipulation
scales (143) – additional visualisation functionality
tidyr (144) – data manipulation
tidyverse (145) – data manipulation
zCompositions (103) – imputation of left-censored data

109. R, R: A language and environment for statistical computing. R Foundation for Statistical Computing, Vienna, Austria. . **https://www.R-project.org**, (Core Team, 2018).

110. B. Bischl *et al.*, BBmisc: Miscellaneous Helper Functions for B. Bischl. https://CRAN.R-project.org/package=BBmisc, (2017).
111. A. Canty, B. Ripley, boot: Bootstrap R (S-Plus) Functions. R package version 1.3-25, (2020).
112. A. Davison, D. Hinkley, *Bootstrap Methods and Their Applications*. (Cambridge University Press, Cambridge, 1997).
113. M. Kuhn, caret: Classification and Regression Training. R package version 6.0-86. https://CRAN.R-project.org/package=caret. (2020).
114. Á. T. Kocsis, N. B. Raja, chronosphere: Earth system history variables. https://doi.org/10.1111/2041-210X.13161. (2019).
115. A. Zeileis, K. Hornik, P. Murrell, Escaping RGBland: Selecting Colors for Statistical Graphics. *Computational Statistics and Data Analysis* **53**, 3259-3270 (2009).
116. G. van den Boogaart, R. Tolosana-Delgado, M. Bren, compositions: Compositional Data Analysis. R package version 2.0-0. https://CRAN.R-project.org/package=compositions, (2020).
117. M. Dowle, A. Srinivasan, data.table: Extension of `data.frame`. R package version 1.13.2. https://CRAN.R-project.org/package=data.table, (2020).
118. W. Gearty, deeptime: Plotting Tools for Anyone Working in Deep Time. R package version 0.2.0. https://CRAN.R-project.org/package=deeptime, (2021).
119. H. Wickham, J. Hester, W. Chang, devtools: Tools to Make Developing R Packages Easier. R package version 2.3.2. https://CRAN.R-project.org/package=devtools, (2020).
120. H. Wickham, R. Francois, dplyr: A Grammar of Data Manipulation. R package version 0.5.0. https://CRAN.R-project.org/package=dplyr, (2016).
121. X. Wang, fANCOVA: Nonparametric Analysis of Covariance. R package version 0.6-1. https://CRAN.R-project.org/package=fANCOVA, (2020).
122. P. Ribeiro Jr, P. Diggle, M. Schlather, R. Bivand, B. Ripley, geoR: Analysis of Geostatistical Data. R package version 1.8-1. https://CRAN.R-project.org/package=geoR, (2020).
123. R. Hijmans, geosphere: Spherical Trigonometry. R package version 1.5-10. https://CRAN.R-project.org/package=geosphere, (2019).
124. V. Vu, ggbiplot: A ggplot2 based biplot. R package version 0.55 http://github.com/vqv/ggbiplot, (2011).
125. D. Wilkins, ggfittext: Fit Text Inside a Box in 'ggplot2'. R package version 0.9.1. https://CRAN.R-project.org/package=ggfittext, (2021).
126. E. Campitelli, ggnewscale: Multiple Fill and Colour Scales in 'ggplot2'. R package version 0.4.5. https://CRAN.R-project.org/package=ggnewscale, (2021).
127. H. Wickham, ggplot2: Elegant Graphics for Data Analysis. Springer-Verlag New York., (2009).
128. B. Auguie, gridExtra: Miscellaneous Functions for "Grid" Graphics. R package version 2.2.1. https://CRAN.R-project.org/package=gridExtra, (2016).
129. B. Gräler, E. J. Pebesma, G. Heuvelink, Spatio-Temporal Interpolation using gstat. *The R Journal* **8**, 204-218 (2016).
130. E. J. Pebesma, Multivariable geostatistics in S: the gstat package. *Computers & Geosciences* **30**, 683-691 (2004).
131. H. Wickham, httr: Tools for Working with URLs and HTTP. R package version 1.4.2. https://CRAN.R-project.org/package=httr, (2020).

132. J. Ooms, The jsonlite Package: A Practical and Consistent Mapping Between JSON Data and R Objects. https://arxiv.org/abs/1403.2805, (2014).
133. L. Scrucca, Model-based {SIR} for dimension reduction. *Computational Statistics & Data Analysis* **5**, 3010-3026 (2011).
134. A. Bender, F. Scheipl, pammtools: Piece-wise exponential additive mixed modeling tools., (2018).
135. R. Kolde, pheatmap: Pretty Heatmaps. R package version 1.0.12. https://CRAN.R-project.org/package=pheatmap, (2019).
136. A. Liaw, M. Wiener, Classification and Regression by randomForest. *R News* **2**, 18-22 (2002).
137. E. Neuwirth, RColorBrewer: ColorBrewer Palettes. R package version 1.1-2. https://CRAN.R-project.org/package=RColorBrewer, (2014).
138. H. Wickham, J. Hester, readr: Read Rectangular Text Data. R package version 1.4.0. https://CRAN.R-project.org/package=readr, (2020).
139. H. Wickham, Reshaping Data with the reshape Package. *Journal of Statistical Software* **21**, 1-20 (2007).
140. R. Bivand, T. Keitt, B. Rowlingson, rgdal: Bindings for the 'Geospatial' Data Abstraction Library. R package version 1.5-27. https://CRAN.R-project.org/package=rgdal. (2021).
141. C.-h. Chan, G. Chan, T. Leeper, J. Becker, rio: A Swiss-army knife for data file I/O. R package version 0.5.26., (2021).
142. A. Fabri, safejoin: Join safely and Deal with Conflicting Columns. R package version 0.1.0., (2021).
143. H. Wickham, D. Seidel, scales: Scale Functions for Visualization. R package version 1.1.1. https://CRAN.R-project.org/package=scales, (2020).
144. H. Wickham, tidyr: Tidy Messy Data. R package version 1.1.2. https://CRAN.R-project.org/package=tidyr, (2020).
145. H. Wickham *et al.*, Welcome to the tidyverse. *Journal of Open Source Software* **4**, 1686 (2019).

***loess* spans**

Regarding loess curve fitting for the pyrite trace element dataset (19-23), we utilized two approaches regarding span (compare Figs. 4, S12, S21-S22). In our first approach, loess CV spans were determined independently for each 0.2 σ increment around the mean loess (also with a CV span). This approach produces loess spans which may vary significantly between 0.2 σ increments and results in a relatively smooth output that is less sensitive to short wavelength variation (particularly at high or low σ). Alternatively, we propagated the mean loess CV span for all 0.2 σ increments, yielding a coherent resampled output that is more sensitive to local variation. Both approaches produced a reasonable fit to the observations and support the same interpretations.

Regarding the Phanerozoic Fe-speciation SGP dataset, due to a large data gap in the Mesozoic-Cenozoic, we spliced two curves at 180 Ma (> 150 Ma, *span* = CV, *degree* =1, < 200 Ma, *span* = 1, *degree* = 0) in order to optimize the curve fitting. All Precambrian curves were generated at *degree* = 0 and span = CV in order to optimize the curves.

Spatiotemporal resampling

We implemented resampling based on the inverse distance weighting approach outlined by ref. (25). Samples closer together in time or space are assigned large proximity values compared to more widely distanced samples. The proximity values are used to weight samples, so that samples which are closer together are less likely to be selected during resampling. Finally, for the purpose of resampling we introduced a very small amount of noise in order to 'jitter' each sample in space and time (this means it is possible to implement alternative interpolation approaches, such as kriging).

We applied two distance functions:

$s = arcdistance(x,x_i)/scale_{spatial}$
$t = age(x,x_i)/scale_{age}$

where:
arcdistance is the Haversine (or great circle) distance, the angular distance between two points (x, x_i) on the surface of a sphere
$scale_{spatial} = 0.5$ (preselected)
age is the difference between the ages of two points (x, x_i)
$scale_{age} = 10$ (preselected)

The proximity value *w* is a sum of the reciprocals of the distance measures between all sample pairs, meaning samples which are further away (in space and/or time) exhibit relatively large proximity values.

$w(x) = \Sigma(1/s^2+1)+1/(t^2+1)$ for $i = n$ samples

The sample proximities in Fig. 4F are based solely on distance and do not consider age [i.e., $w(x) = \Sigma(1/s^2+1)$]. Whereas weighting (see below) for the spatial interpolation of the combined xDD, pyrite trace element and SGP datasets was calculated using both the spatial and age proximities.

Weighting (or probability of selection) was defined as the inverse of *w* and centred approximately to a 1-in-5 probability of selection. See Figure S24 for the plotted probabilities of selection.

$P(x) = 1/(w(x)*median(2/w))+1$

Spatial interpolation was conducted using the inverse distance weighting (idw) function of the *gstat* R package:

$w(x) = 1/d(x, x_i)^p$

where
d = the sum of the distances between each sample pair
p = 2 (default)

Text mining

Guide to the xDD public-facing dataset

The xDD simplified results (*results.csv)* contains the following column headings; *result_id, docid, sentid, target_word, strat_phrase_root, strat_flag, strat_name_id, in_ref, source* and *phrase*. Since the original publications are copyrighted, and cannot be distributed publicly *en masse*, we condensed the raw phrases into the tuples and contextual phrases of interest.

For example, the condensed phrase:
"pyritic concretions - Quarry Hill Member [silt, mud]"

corresponds to the complete phrase:
"…The Quarry Hill Member is a medium to very dark silty mudstone with pyritic concretions common throughout and almost no fossils…"

In the above example (from Marintsch and Finks, 1978), NLP (Fig. S1) delineates the tuple between *pyrite concretions* and the Quarry Hill Member. To remove all non-(meta)sedimentary rocks and to search for occurrences of pyrite veins/mineralization and evaporitic rocks/minerals, we searched for mentions in (1) the stratigraphic package or unit name itself (e.g., Kimmeridge *Clay* Formation); (2) the complete phrase (in this example: *mud, silt*, i.e., a relaxed approach), (3) the accompanying lithological description(s) for the matched stratigraphic package (e.g., https://ngmdb.usgs.gov/Geolex/Units/QuarryHill_14206.html), where present in the focal area. The (meta)sedimentary rock record is therefore a first-order approximation only. We searched for the following sedimentary and metasedimentary rock types recorded in Macrostrat https://macrostrat.org/api/defs/lithologies?all:

siliciclastic, gravel, sand, silt, mud, claystone, mudstone, shale, siltstone, sandstone, arkose, greywacke, conglomerate, breccia, diamictite, mixed carbonate-siliciclastic, carbonate, marl, lime mudstone, wackestone, packstone, grainstone, boundstone, floatstone, rudstone, bafflestone, bindstone, framestone, limestone, dolomite, siderite, ankerite, evaporite, halite, gypsum, anhydrite, coal, peat, lignite, anthracite, tar, chert, phosphorite, ironstone, iron formation, volcaniclastic, slate, phyllite, quartzite, hornfels, marble, argillite, chalk, clay, till, diatomite, calcareous ooze, bauxite, sedimentary, siliceous ooze, graywacke, dolostone, micrite, greensand, grit, paragneiss, loess, metasedimentary, coquina, flint, oolite, metapelite, arenite, pelite, biomicrite, oomicrite, biosparite, pelmicrite, oosparite, pelsparite, intrasparite, intramicrite, novaculite, travertine, trona, encrinite, alluvium, colluvium, regolith, soil, paleosol, laterite, eluvium, subarkose, quartz arenite, skarn, gyttja, drift, tillite, metaconglomerate, wacke, tufa, metagraywacke, metasiltstone, litharenite, sublitharenite, radiolarite, calcarenite, calcilutite, diamicton, porcellanite, sediment

Result_id, docid and *sentid* are xDD identifiers. It is possible to cross-reference *docid* in *results.csv* with the *docid* recorded in the reference list *pyrite_refs.txt. target_word* represents the target search word or phrase (e.g., framboidal pyrite). *strat_phrase_root* corresponds to the name of the stratigraphic package or unit (for example Quarry Hill). *strat_flag* corresponds to the stratigraphic hierarchy (e.g., Member, Formation, Group, Supergroup etc.) *in_ref* indicates a match inside or outside a reference list.

Manual assessment of Precambrian framboid mentions

Manual assessment of Precambrian pyrite framboid mentions (n = 39, all stratigraphic package/unit mentions, per unique document)
32 verified hits
5 erroneous or false positive hits
2 unverified hits
87 % accuracy

Criddle, A.J., NA, A Preliminary Description of Microcrystalline Pyrite from the Nannoplankton Ooze at Site 251, Southwest Indian Ocean, Initial Reports of the Deep Sea Drilling Project, 26, , , 10.2973/dsdp.proc.26.126.1974, http://deepseadrilling.org/26/dsdp_toc.htm
Negaunee Iron, http://ngmdb.usgs.gov/Geolex/Units/Negaunee_2962.html
One lOµ diameter spheroid , with the same optical characteristics as the groundmass , was located : it was not framboidal and is best interpreted as an iron-oxide spheroid , closely resembling those described by Lougheed and Mancuso (1973) from the Negaunee Iron Formation , Michigan .`` Gangue '' Material This account of the microcrystalline pyrite would not be complete without a summary of the major constituents of the sediment in which the pyrite , which forms a proportionally minor part , was found . False positive

Pratt, Lisa M.; Summons, Roger E.; Hieshima, Glenn B., 1991, Sterane and triterpane biomarkers in the Precambrian Nonesuch Formation, North American Midcontinent Rift, Geochimica et Cosmochimica Acta, 55, 3, 911--916, 10.1016/0016-7037(91)90351-5, http://www.sciencedirect.com/science/article/pii/0016703791903515
Nonesuch, http://ngmdb.usgs.gov/Geolex/Units/Nonesuch_3017.html
Photomicrographs illustrating sedimentary textures of silty shales from the Nonesuch Formation , iron River syncline , Michigan .(a) Sample 688-38 , submillimeter-scale lamination composed of quartz silt layers and dark-colored layers of clay , framboidal pyrite , and organic matter
Verified

Wacey, David; Saunders, Martin; Cliff, John; Kilburn, Matt R.; Kong, Charlie; Barley, Mark E.; Brasier, Martin D., 2014, Geochemistry and nano-structure of a putative â˘¼3240 million-year-old black smoker biota, Sulphur Springs Group, Western Australia, Precambrian Research, , , 1--12, 10.1016/j.precamres.2014.04.016, http://www.sciencedirect.com/science/article/pii/S0301926814001454
Fermeuse, http://weblex.nrcan.gc.ca/html/004000/GSCC00053004904.html
We find near identical patterns of sponge-like pyrite surrounding pure pyrite nano-crystals in analogous framboids from the ~ 560 Ma Fermeuse Formation of Newfoundland (Fig. 5c) , showing that these distinctive textures can be preserved all the way back to the Precambrian rock record . Verified – although debatable definition of ‘framboid’

Liu, Alexander G., 2016, Framboidal Pyrite Shroud Confirms The ‘Death Mask' Model For Moldic Preservation Of Ediacaran Soft-Bodied Organisms, Palaios, 31, 5, 259--274, 10.2110/palo.2015.095, http://dx.doi.org/10.2110/palo.2015.095
Fermeuse, http://weblex.nrcan.gc.ca/html/004000/GSCC00053004904.html
Previous suggestions that pyrite might have played a role in macrofossil preservation in Newfoundland are limited to elevated Fe and S concentrations in sediments surrounding

Aspidella fossils in the Fermeuse Formation (La<U+FB02>amme et al. 2011) , and rare pyrite in bedding-parallel wisps and euhedral blocky crystals associated with macrofossil-bearing horizons at Spaniard 's Bay (Brasier et al. 2013 , <U+FB01>g .3C -- 3F) and Back Cove (Liu et al. 2014a ; Wacey et al. 2015) .The results presented herein demonstrate that microbially formed pyrite veneers are ubiquitous immediately above fossil-bearing surfaces in Newfoundland , revealing that Gehling 's ` death mask ' model is applicable to deep-marine settings .The <U+FB01>ndings also raise the possibility that un<U+FB01>gured structures historically assigned to the microfossil taxon Bavlinella from the St. John 's Group (Timofeyev et al. 1980 ; Anderson et al. 1982) may actually record pyrite framboids . Verified

Kelly, William C.; Nishioka, Gail K., 1985, Precambrian oil inclusions in late veins and the role of hydrocarbons in copper mineralization at White Pine, Michigan, Geol, 13, 5, 334, 10.1130/0091-7613(1985)13<334:poiilv>2.0.co;2, http://dx.doi.org/10.1130/0091-7613(1985)13<334:poiilv>2.0.co;2
Nonesuch, http://ngmdb.usgs.gov/Geolex/Units/Nonesuch_3017.html
On the basis of abrupt disappearance of normal Nonesuch pyrite as shale beds enter the ore zone , and of the occurrence of some Cu-Fe sulfide pseudomorphs of pyrite framboids and euhedra within the nar - GEOLOGY , May 1985 335 row `` fringe , '' Brown (1971) concluded that replacement of diagenetic pyrite was the principal copper control in the main ore zone . Verified

Winter, Bryce L.; Knauth, L.Paul, 1992, Stable isotope geochemistry of early Protezozoic carbonate concretions in the Animikie group of the Lake Superior region: evidence for anaerobic bacterial processes, Precambrian Research, 54, 2-4, 131--151, 10.1016/0301-9268(92)90067-X, http://www.sciencedirect.com/science/article/pii/030192689290067X
Gunflint Iron, http://ngmdb.usgs.gov/Geolex/Units/Gunflint_13079.html
Neither of the concretions from the Gunflint Iron Formation display isotopic systematics that can be used to suggest that organic matter degradation processes were occurring in conjunction with carbonate precipitation .In fact , the fil3C values of both concretions are nearly within the range of shallow marine carbonate .This is somewhat surprising considering the abundance of concretionary (< 1 mm to 3 cm in diameter) and framboidal pyrite in the host shale . Verified

Cloud, Preston, 1976, Beginnings of biospheric evolution and their biogeochemical consequences, Paleobiology, 2, 04, 351--387, 10.1017/S009483730000498X, https://www.cambridge.org/core/product/identifier/S009483730000498X/type/journal_article
Belt, http://ngmdb.usgs.gov/Geolex/Units/Belt_6823.html
an affinity with forms described by Pflug (1966) from the much younger Belt Supergroup of Montana .Other deceptive non-biogenic look-alikes for real microorganisms are provided by various spherulitic or colloform structures in glassy or cherty rocks (e.g. Tyler and Barghoorn 1954 , figs. 1-2) .In addition , spheroidal , framboidal pyrite or antecedent marcasite of non-biologic origin may take on a very lifelike appearance , (P l a t e 1 , figure 2) , or , b y growing in car bonaceous sediments , may aquire a carbona ceous exterior coating or even , and apparently commonly , may also infiltrate real microor ganisms (e.g. Moorman 1974) . Not verified (unable to access)

Park, John K., 1995, Paleomagnetism of the late Neoproterozoic Blueflower and Risky formations of the northern Cordillera, Canada, Canadian Journal of Earth Sciences, 32, 6, 718--729, 10.1139/e95-061, http://www.nrcresearchpress.com/doi/abs/10.1139/e95-061
Blueflower, http://weblex.nrcan.gc.ca/html/001000/GSCC00053001558.html
The Blueflower locality contains much pyrite , which occurs as single grains of cubic or framboidal aspect , and as overgrowths . Not verified (unable to access)

McIlroy, Duncan, 2000, A lower Cambrian protoconodont apparatus from the Placentian of southeastern Newfoundland, Lethaia, 33, 2, 95--102, 10.1080/00241160050150230, http://doi.wiley.com/10.1080/00241160050150230
Chapel Island, http://weblex.nrcan.gc.ca/html/002000/GSCC00053002753.html
± 10 m above Limestone 1 , Member 4 of the Chapel Island Formation , Placentian Series , Lower Cambrian .Material .± One cluster composed of three naturally arranged elements .Two of the elements are almost completely composed of pyrite framboids (Fig. 2E , F) ; the third is preserved in form of an imprint only . Verified

Wilde, A. R., 2011, Mount Isa copper orebodies: improving predictive discovery, Australian Journal of Earth Sciences, 58, 8, 937--951, 10.1080/08120099.2011.571285, http://www.tandfonline.com/doi/full/10.1080/08120099.2011.571285
Mount Isa, http://dbforms.ga.gov.au/pls/www/geodx.strat_units.sch_full?wher=stratno=12822
Chemical depositional processes Wallrock reaction between ore <U+FB02>uid and pyrite , carbonate , and/or carbon and <U+FB02>uid mixing have all been suggested as critical chemical controls on epigenetic copper mineralisation at Mount Isa (Andrew et al. 1989 ; Heinrich et al. 1989 ; Valenta 1994) .Beds rich in early framboidal pyrite could have provided both Fe and reduced S . Verified

Strauss, Harald; Schieber, Jeurgen, 1990, A sulfur isotope study of pyrite genesis: The mid-proterozoic Newland formation, belt supergroup, Montana, Geochimica et Cosmochimica Acta, 54, 1, 197--204, 10.1016/0016-7037(90)90207-2, http://www.sciencedirect.com/science/article/pii/0016703790902072
Newland, http://ngmdb.usgs.gov/Geolex/Units/Newland_9523.html
Pyrite types in the Newland Formation Pyrite in the Newland Formation has been described in detail by SCHIEBER (1985) .Most commonly observed is fine crystalline pyrite (I - 10 microns) , which may be irregularly scattered throughout the rock , form framboids (0.02-0 .25 mm) or aggregate into thin lenticular-wavy laminae .The latter form laminated pyrite beds (may contain up to 50 % pyrite by volume) which alternate with shale beds with little or no pyrite and produce a characteristic striped appearance (Fig. 3) . Verified

Pirajno, Franco; Burlow, Rick; Huston, David, 2010, The Magellan Pb deposit, Western Australia; a new category within the class of supergene non-sulphide mineral systems, Ore Geology Reviews, 37, 2, 101--113, 10.1016/j.oregeorev.2010.01.001, http://www.sciencedirect.com/science/article/pii/S0169136810000120
Yelma, http://dbforms.ga.gov.au/pls/www/geodx.strat_units.sch_full?wher=stratno=20942
The upper part of the Formation , as shown in a 60 m-section at a locality where the disconformity with the overlying Yelma Formation is exposed (detailed in Pirajno et al. , 2009) , consists of massive to <U+FB02>aggy dolostone and argillite beds exhibiting decimetre-scale

rhythmic banding and metre-scale wavy banding .The dolostone is commonly pink to purple -- brown in colour and <U+FB02>ecked with iron and manganese oxides .The lower parts of the formation consist of carbonaceous facies with local bands of marlstone and carbonate nodules up to 50 cm in diameter .Drillcore and drill cuttings show sulphides (predominantly pyrite) that are locally abundant as cubes , framboidal aggregates , and nodules ; and bands of carbonaceous and calcareous argillite with carbonate concretions , some with sulphide cores . Verified [strictly Maralou Fm.]

Lyons, Timothy W; Luepke, James J; Schreiber, Madeline E; Zieg, Gerald A, 2000, Sulfur geochemical constraints on mesoproterozoic restricted marine deposition: lower Belt Supergroup, northwestern United States, Geochimica et Cosmochimica Acta, 64, 3, 427--437, 10.1016/S0016-7037(99)00323-3, http://www.sciencedirect.com/science/article/pii/S0016703799003233
Newland, http://ngmdb.usgs.gov/Geolex/Units/Newland_9523.html
A : Well-developed pyrite framboid within a dominantly silicate matrix , Newland Formation black shale . Verified

Armstrong, Joseph G.T.; Parnell, John; Bullock, Liam A.; Perez, Magali; Boyce, Adrian J.; Feldmann, Jorg, NA, Tellurium, selenium and cobalt enrichment in Neoproterozoic black shales, Gwna Group, UK: deep marine trace element enrichment during the Second Great Oxygenation Event, Terra Nova, , , , 10.1111/ter.12331, http://doi.wiley.com/10.1111/ter.12331
Gwna, http://www.bgs.ac.uk/lexicon/lexicon.cfm?pub=NGW
Framboids are 5 -- 10 lm in diameter and are disseminated throughout the Gwna Group black shale , while euhedral pyrites are generally larger (0.05 -- 2 mm) and present as discrete lenses within the unit (Figure 5b) .Clausthalite inclusions occur exclusively within the framboids , while Ni -- Co -- As phases are limited to the euhedral morphologies .Euhedral pyrites are observed to overgrow the framboids (Figure 5c) .LA-ICP-MS confirms that Se within the black shale occurs as evenly distributed , discrete PbSe phases , associated with micronscale pyrite , while Ni -- Co -- As mineralisation trends with larger pyrite (Figure 6) . Verified

Winter, Bryce L.; Knauth, L.Paul, 1992, Stable isotope geochemistry of cherts and carbonates from the 2.0 Ga gunflint iron formation: implications for the depositional setting, and the effects of diagenesis and metamorphism, Precambrian Research, 59, 3-4, 283--313, 10.1016/0301-9268(92)90061-R, http://www.sciencedirect.com/science/article/pii/030192689290061R
Gunflint, http://ngmdb.usgs.gov/Geolex/Units/Gunflint_13079.html
Pyrite is not an abundant phase in the banded facies of the Gunflint , but it is very prominent in the carbonaceous shale facies as framboids and centimeter-sized nodules which Carrigan and Cameron (1991) report to have t ~ 34S values consistent with a bacterial origin . Verified

, 2008, Goldschmidt Abstracts 2008- I, Geochimica et Cosmochimica Acta, 72, 12, A406--A417, 10.1016/j.gca.2008.05.012, http://www.sciencedirect.com/science/article/pii/S0016703708002676
Gunflint, http://ngmdb.usgs.gov/Geolex/Units/Gunflint_13079.html
Such contrast in Fe-bearing minerals in chemical sediments suggests the stratified oxic-anoxic oceans during deposition of the Gunflint Formation .The high productivity of microbes at around the shallowand deep-water sequences is suggested by the local occurrence of phosphorites .As-

rich framboidal-like pyrite was found with euhedral pyrite around such phosphorites , suggesting that sulfate-reducers were active both in anoxic parts of ocean waters and also in sediments . Verified

Hiatt, Eric E.; Pufahl, Peir K.; Edwards, Cole T., 2015, Sedimentary phosphate and associated fossil bacteria in a Paleoproterozoic tidal flat in the 1.85Ga Michigamme Formation, Michigan, USA, Sedimentary Geology, , , 24--39, 10.1016/j.sedgeo.2015.01.006, http://www.sciencedirect.com/science/article/pii/S003707381500038X
Gunflint Iron, http://ngmdb.usgs.gov/Geolex/Units/Gunflint_13079.html
The Bijiki , like the correlative Gun<U+FB02>int Iron Formation , does not contain 30 E.E. Hiatt et al. / Sedimentary Geology 319 (2015) 24 -- 39 Fig. 7 .Paragenesis of major mineral phases and microstructures including fossil bacteria presented here .Fossil bacteria occur in francolite that contains pyrite framboids and small (generally b 2 μm diameter) pyrite crystals that , although rare , can have euhedral overgrowths up to 30 μm in diameter . Verified [time-equivalent to Gunflint]

Richard M. Pollastro, 1981, Authigenic Kaolinite and Associated Pyrite in Chalk of the Cretaceous Niobrara Formation, Eastern Colorado, SEPM Journal of Sedimentary Research, Vol. 51, , , 10.1306/212f7cd4-2b24-11d7-8648000102c1865d, http://dx.doi.org/10.1306/212f7cd4-2b24-11d7-8648000102c1865d
Miette, http://weblex.nrcan.gc.ca/html/009000/GSCC00053009755.html
JAVOR , B. J. , ANDMOUNTJOY , E. W. , 1976 , Late Proterozoic microbiota of the Miette Group , Southern British Columbia : Geology , v. 4 , p. 111-119 .JEANS , C. V. , 1968 , The origin of the montmorillonite of the European chalk , with special reference to the Lower Chalk of England : Clay Minerals , v. 7 , p. 311-329 .KALLIOKOWS ~ , J. , AND CAXmES , L. , 1969 , Morphology , lization of kaolinite and framboidal pyrite or its mode of formation and diagenetic changes in framboids : precursor . Erroneous result

Retallack, Gregory J.; Krinsley, David H.; Fischer, Robert; Razink, Joshua J.; Langworthy, Kurt A., 2016, Archean coastal-plain paleosols and life on land, Gondwana Research, , , 1--20, 10.1016/j.gr.2016.08.003, http://www.sciencedirect.com/science/article/pii/S1342937X16301770
Pilbara, http://dbforms.ga.gov.au/pls/www/geodx.strat_units.sch_full?wher=stratno=15189
This result has been challenged for pyrite in Pilbara sediments (Kojima et al. , 1998) , but the rounded nature of both authigenic framboids and detrital pyrite makes such distinctions difficult (Fig. 5F-H) . Verified

Olempska, Ewa; Wacey, David, 2016, Ambient inclusion trails in Palaeozoic crustaceans (Phosphatocopina and Ostracoda), Palaeogeography, Palaeoclimatology, Palaeoecology, 441, , 949--958, 10.1016/j.palaeo.2015.10.052, http://www.sciencedirect.com/science/article/pii/S0031018215006239
Gunflint, http://ngmdb.usgs.gov/Geolex/Units/Gunflint_13079.html
2) from the Gun<U+FB02>int Formation , Ontario , but differ in the occurrence of randomly oriented trails .Microtubes with a diameter ranging from 4 to 7.7 μm , a maximum 10 μm length and terminated with framboidal rather than euhedral pyrite , occur only rarely . Erroneous result

Nelson, Gabriel J.; Pufahl, Peir K.; Hiatt, Eric E., 2010, Paleoceanographic constraints on Precambrian phosphorite accumulation, Baraga Group, Michigan, USA, Sedimentary Geology, 226, 1-4, 9--21, 10.1016/j.sedgeo.2010.02.001, http://www.sciencedirect.com/science/article/pii/S0037073810000321
Baraga, http://ngmdb.usgs.gov/Geolex/Units/Baraga_6671.html
Its highest concentration in the Baraga Group occurs within these organic-rich hemipelagites .Framboidal pyrite is much less abundant in prodelta siltstones (F4) and is absent in delta front deposits (F3) of the TST and HST . Verified

Lalonde, Stefan V.; Pecoits, Ernesto; von Gunten, Konstantin; Robbins, Leslie J.; Alessi, Daniel S.; Philippot, Pascal; Konhauser, Kurt O., 2018, Petrology and geochemistry of the Boolgeeda Iron Formation, Hamersley Basin, Western Australia, Precambrian Research, 316, , 155--173, 10.1016/j.precamres.2018.07.015, https://www.sciencedirect.com/science/article/pii/S0301926817306290
Boolgeeda Iron, http://dbforms.ga.gov.au/pls/www/geodx.strat_units.sch_full?wher=stratno=2227
Notably , martite and geothite are absent from thin sections , suggesting microplaty hematite within the Boolgeeda Iron Formation is not a paragenetic product of alteration typically associated with the formation of high grade hematite ore .Further , minnesotaite and stilpnomelane appear absent from thin section , attesting to the pristine nature of the iron bands .The prevalence of chlorite in the green mudstone/siltstones is likely the result of metamorphic alteration of primary clay material that composed these intervals .Philippot et al. (2018) documented the presence of multiple pyrite textures within TCDP1 , including nodular pyrite aggregates , clusters of microcrystalline pyrite , inclusion free pyrite overgrowths around pyrite framboids , <U+FB01>nely disseminated euhedral to subhedral pyrite crystals , euhedral to subhedral pyrite crystals aligned with bedding and conforming to soft sediment deformation features , as well as bands of densely packed microcrystalline pyrite aggregates . Verified [via Philippot et al. SI]

Allison, Carol W.; Moorman, Mary A., 1974, Pyritized Microfossils and Pyrite Framboids: Reply, Geol, 2, 4, 202, 10.1130/0091-7613(1974)2<202:pmapfr>2.0.co;2, http://dx.doi.org/10.1130/0091-7613(1974)2<202:pmapfr>2.0.co;2
Tindir, http://ngmdb.usgs.gov/Geolex/Units/Tindir_12100.html
References Cited Allison , C. W. , and Moorman , M. A. , 1973 , Microbiota from the late Proterozoic Tindir Group , Alaska : Geology , v. 1 , no. 2 , p. 6 5 - 6 8 .Kalliokoski , J. , 1974 , Pyrite framboids : Animal , vegetable , or mineral ? Verified via Allison and Moorman 1973, assuming origin of Sorg alteration surrounding pyrite framboids – see Emmings et al. 2019. RPP.

Allison, C.W., 1988, Paleontology of late Proterozoic and Early Cambrian rocks of east-central Alaska, Professional Paper, , , , 10.3133/pp1449, https://pubs.er.usgs.gov/publication/pp1449
Tindir, http://ngmdb.usgs.gov/Geolex/Units/Tindir_12100.html
Similarity of the pyrite-replaced multiunits described from these samples to pyrite framboids led Kalliokoski (1974) to interpret a nonbiologic origin for this Tindir material . Verified

Mapstone, N.B.; McIlroy, D., 2006, Ediacaran fossil preservation: Taphonomy and diagenesis of a discoid biota from the Amadeus Basin, central Australia, Precambrian Research, 149, 3-4, 126-

-148, 10.1016/j.precamres.2006.05.007,
http://www.sciencedirect.com/science/article/pii/S030192680600129X
Arumbera, http://dbforms.ga.gov.au/pls/www/geodx.strat_units.sch_full?wher=stratno=668
Pyrite sole-veneer A thorough SEM search for framboidal pyrite (or iron oxide pseudomorphs) along the fossiliferous hyporelief surfaces in the present Arumbera Sandstone samples , N.B. Mapstone , D. McIlroy / Precambrian Research 149 (2006) 126 -- 148 137 provides no direct evidence for a pyrite sole-veneer . Tentative erroneous result

Foster, Clinton B.; Robbins, Eleanora I.; Bone, Yvonne, 1990, Organic tissues, graphite, and hydrocarbons in host rocks of the Rum Jungle Uranium Field, northern Australia, Ore Geology Reviews, 5, 5-6, 509--523, 10.1016/0169-1368(90)90050-W,
http://www.sciencedirect.com/science/article/pii/016913689090050W
Whites, http://dbforms.ga.gov.au/pls/www/geodx.strat_units.sch_full?wher=stratno=24572
Pyrite is most abundant in the residue from a black argillite from the Whites Formation (1156) ; macroscopic observations are in agreement (Table 1) .In other samples , pyrite forms a small component of the rocks .Pyrite occurs in all samples as disseminated cubes , pyritohedrons , and as masses having no recognizable crystal form ; much of it is enmeshed in the black tissue .No framboids , the primary form of sedimentary pyrite formed by sulfur bacteria , were present .
False positive

Grandstaff, D.E., 1980, Origin of uraniferous conglomerates at Elliot Lake, Canada and Witwatersrand, South Africa: Implications for oxygen in the Precambrian atmosphere, Precambrian Research, 13, 1, 1--26, 10.1016/0301-9268(80)90056-X,
http://www.sciencedirect.com/science/article/pii/030192688090056X
Elliot Lake, http://weblex.nrcan.gc.ca/html/004000/GSCC00053004584.html
There has been long discussion of the origin of pyrite in both the Witwatersrand and Elliot Lake deposits (Liebenberg , 1957 ; Ramdohr , 1958 ; Davidson , 1960 , 1965 ; Schidlowski and Trurnit , 1966 ; Roscoe , 1969 ; Theis , 1978 ; Utter , 1978 ; and others) .Part of the pyrite appears to be detrital .However , observations of pyrite overgrowths and framboidal pyrite indicate that some of the pyrite is of post-depositional , epigenetic origin . Verified

Kendall, Brian; Creaser, Robert A.; Gordon, Gwyneth W.; Anbar, Ariel D., 2009, Reâ€Os and Mo isotope systematics of black shales from the Middle Proterozoic Velkerri and Wollogorang Formations, McArthur Basin, northern Australia, Geochimica et Cosmochimica Acta, 73, 9, 2534--2558, 10.1016/j.gca.2009.02.013,
http://www.sciencedirect.com/science/article/pii/S0016703709001021
Wollogorang, http://dbforms.ga.gov.au/pls/www/geodx.strat_units.sch_full?wher=stratno=20358
A $ 20 m thick unit of dolomitic , pyritic , and <U+FB01>nely laminated organic-rich shale (total organic carbon -LSB- TOC -RSB- up to 6 % ; Donnelly and Jackson , 1988) occurs within the upper part of the lower Wollogorang Formation .Disseminated pyrite within the black shale is typically <U+FB01>ne-grained and euhedral (i.e. , syn-depositional pyrite formation within a euxinic water column ; Wilkin et al. , 1997) with occasional framboidal forms (Shen et al. , 2002) .Coarse pyrite aggregates are also observed within early diagenetic , bituminous dolomite nodules . Verified [via Shen et al. 2002]

Davidson, G. J., 1998, Alkali alteration styles and mechanisms, and their implications for a 'brine factory' source of base metals in the rift-related McArthur group, Australia, Australian Journal of Earth Sciences, 45, 1, 33--49, 10.1080/08120099808728365, http://www.tandfonline.com/doi/abs/10.1080/08120099808728365
Barney Creek, http://dbforms.ga.gov.au/pls/www/geodx.strat_units.sch_full?wher=stratno=1130
K-feldspars from the mineralised horizons are also notably far coarser than those regionally developed in the Barney Creek Formation (Logan 1979) .The zonation from K-feldspar to albite is interpreted to result from hydrostatically driven saline groundwater flow focused from the basin margin (Emu Fault Zone) , a model based on evidence of low fluid temperatures determined from oxygen-isotope pairs and the identification of the source water as meteoric (Davidson in press) .A diagenetic timing is favoured for this because : (i) glass shards at HYC were feldspathised prior to the development of burialrelated dissolution seams (Figure 4b , c) ; (ii) the feldspar fabric overgrew early diagenetic framboidal pyrite (known locally as pyl) that developed from the activity of sulfatereducing bacteria (Logan 1979 ; Eldridge et al. 1993) ; the shapes of uncompacted vitriclasts are preserved within this pyrite but are destroyed elsewhere ; and (iii) microcline + quartz infills secondary porosity in vitriclastic beds . Verified

Hieshima, G.B.; Pratt, L.M., 1991, Sulfur/carbon ratios and extractable organic matter of the middle proterozoic nonesuch formation, north american midcontinent rift, Precambrian Research, 54, 1, 65--79, 10.1016/0301-9268(91)90069-M, http://www.sciencedirect.com/science/article/pii/030192689190069M
Nonesuch, http://ngmdb.usgs.gov/Geolex/Units/Nonesuch_3017.html
The sulfur to carbon ratios and the intimate association fo framboidal pyrite and organic matter are suggested to he the result of bacterial sulfate reduction in the Nonesuch sediments . Verified

DARROCH, 2018, EDIACARAN-STYLE DECAY EXPERIMENTS USING MOLLUSKS AND SEA ANEMONES, PALAIOS, 33, 5, 185--203, 10.2110/palo.2017.091, http://dx.doi.org/10.2110/palo.2017.091
Fermeuse, http://weblex.nrcan.gc.ca/html/004000/GSCC00053004904.html
In addition , analyses of Aspidella in cross-section from the Fermeuse Formation , Newfoundland , uncovered pyrite and iron oxides disseminated within thin clay layers enveloping the fossils (La<U+FB02>amme et al. 2011) .Further , pyritized microbial <U+FB01>laments have been discovered in association with Ediacaran fossil deposits in Russia (Callow and Brasier 2009) , and remineralized pyrite framboids have been described from deep water fossil surfaces associated with turbidite <U+FB02>ows (Liu et al. 2015) .
Verified as framboids in the time-equivalent Doushantuo Fm (via Wang et al. 2012)

Mathieu, J.; Kontak, D.J.; Turner, E.C.; Fayek, M.; Layne, G., 2015, Geochemistry of Phanerozoic Diagenesis on Victoria Island, NWT, Canada, Chemical Geology, , , , 10.1016/j.chemgeo.2015.08.016
Wynniatt, http://weblex.nrcan.gc.ca/html/016000/GSCC00053016736.html
Paleogeographic reconstructions , together with oxygenated signatures suggest that precipitation of calcite cement occurred sometime after the Ellesmerian Orogeny , when Victoria Island was at comparatively high latitude and the Wynniatt Formation was exhumed to depths less than 1 km .Although not associated with any mineralisation , characteristics of the calcite cement resemble those of the late calcite cement at the Polaris deposit .The Victoria Island

formation dolostone and cements (quartz , dolomite 1 , and dolomite 2) , indicate a complex <U+FB02>uid history that commenced with possible hydrothermal <U+FB02>uids that silici<U+FB01>ed the host dolostone .Subsequently , a high-salinity , quartz-precipitating <U+FB02>uid that had interacted with an underlying lithology (e.g. , Quyuk formation) at depth mixed with a reduced-sulphur-bearing <U+FB02>uid on-site to precipitate cogenetic quartz and framboidal pyrite . Verified but note late diagenetic framboid origin

Wacey, D.; Kilburn, M. R.; Saunders, M.; Cliff, J. B.; Kong, C.; Liu, A. G.; Matthews, J. J.; Brasier, M. D., 2014, Uncovering framboidal pyrite biogenicity using nano-scale CNorg mapping, Geology, 43, 1, 27--30, 10.1130/g36048.1, http://dx.doi.org/10.1130/g36048.1
Fermeuse, http://weblex.nrcan.gc.ca/html/004000/GSCC00053004904.html
Example of sulfur isotope data (d34S ‰) obtained in situ from framboidal pyrite in the ca. 560 Ma Fermeuse Formation , Newfoundland (Canada) . Verified

Slotznick, Sarah P.; Webb, Samuel M.; Kirschvink, Joseph L.; Fischer, Woodward W., NA, Mid-Proterozoic ferruginous conditions reflect post-depositional processes, Geophysical Research Letters, , , , 10.1029/2018GL081496,
https://onlinelibrary.wiley.com/doi/abs/10.1029/2018GL081496
Newland, http://ngmdb.usgs.gov/Geolex/Units/Newland_9523.html
*(a) Recrystallized framboidal iron sulfides in the Newland Formation (T095-389) , (b) pyrite disaggregated framboids from the Appekunny Formation , east Glacier National Park (GP14-35) with arrow pointing in field up-direction , (c) FeTiMnO grain and surrounding coarse-grained matrix from the Prichard Formation (BS13-37) , (d) small pyrite grains within calcite nodule rimmed by chlorite with arrows pointing out Fe-dolomite and zoned calcite/Fe dolomite grains in the Appekunny Formation , west Glacier National Park (GP14-27) , (e) dolomite (Dol) rimmed by Fe-bearing dolomite (Fe-Dol) in the Appekunny Formation , east Glacier National Park (GP14-35) , and (f) Fe-bearing dolomite cements in the Newland Formation (T112-334) .*Verified

Schieber, Juergen, 1989, Pyrite mineralization in microbial mats from the mid-Proterozoic Newland Formation, Belt Supergroup, Montana, U.S.A., Sedimentary Geology, 64, 1-3, 79--90, 10.1016/0037-0738(89)90085-7,
http://www.sciencedirect.com/science/article/pii/0037073889900857
Newland, http://ngmdb.usgs.gov/Geolex/Units/Newland_9523.html
Whereas scattered and framboidal pyrite is a common minor constituent (up to 4 %) of all the shales in the Newland Formation , laminated pyrite beds are only found in distinct horizons of pyritic shale .Laminated pyrite Laminated pyrite beds are some millimeters to several centimeters thick , and are separated by beds of dolomitic clayey shale (Fig. 3) . Verified

Hiatt, Eric E.; Pufahl, Peir K.; Edwards, Cole T., 2015, Sedimentary phosphate and associated fossil bacteria in a Paleoproterozoic tidal flat in the 1.85Ga Michigamme Formation, Michigan, USA, Sedimentary Geology, , , 24--39, 10.1016/j.sedgeo.2015.01.006,
http://www.sciencedirect.com/science/article/pii/S003707381500038X
Michigamme, http://ngmdb.usgs.gov/Geolex/Units/Michigamme_2753.html
The Michigamme Formation accumulated near the end of the Earth 's initial phosphogenic episode (ca. 2.2 and 1.8 Ga) to produce one of the <U+FB01>rst granular phosphorites

.Phosphatic lithofacies consist of <U+FB01>ne - to medium-sand-sized francolite peloids concentrated on bedding surfaces in peritidal facies .Granular beds are up to 2 cm thick and peloids are often partially to completely replaced by dolomite and chert .The grains contain organic matter and pyrite framboids that suggest bacterial breakdown of organic matter and bacterial sulfate reduction . Verified

Winter, Bryce L.; Knauth, L.Paul, 1992, Stable isotope geochemistry of early Protezozoic carbonate concretions in the Animikie group of the Lake Superior region: evidence for anaerobic bacterial processes, Precambrian Research, 54, 2-4, 131--151, 10.1016/0301-9268(92)90067-X, http://www.sciencedirect.com/science/article/pii/030192689290067X
Rove, http://ngmdb.usgs.gov/Geolex/Units/Rove_14330.html
The total ~ 345 range of + 6.5 TABLE 4 Sulfur isotopic composition and description of pyrite in carbonate concretions of the Rove Formation Sample No .d ; 34S Description Pyrite R2-12 R6-4 R6-5 R6-4 RI 1-4-5 R13-3-1 R13-3-2 R13-3-4 R13-3-6 R13-3-8 R13-3-9 RI5 R1-530 +14.3 +6.5 +6.8 +7.5 + 12.0 +9.9 +13.5 +11.4 +12.7 + 10.7 + 10.1 +7.0 +6.8 disseminated microscopic framboids euhedral pyrite (3 mm diameter) continuous lamina 10 cm spheriodal concretion core 1 mm cubes and disseminated framboids disseminated microscopic framboids 1mm cubes and disseminated framboids disseminated microscopic framboids disseminated microscopic framboids disseminated microscopic framboids disseminated microscopic framboids framboidal , concretion rim euhedral pyrite (3 mm diameter) Isotopic data given in per mil (% 0) . Verified

Retallack, Gregory J.; Krinsley, David H.; Fischer, Robert; Razink, Joshua J.; Langworthy, Kurt A., 2016, Archean coastal-plain paleosols and life on land, Gondwana Research, , , 1--20, 10.1016/j.gr.2016.08.003, http://www.sciencedirect.com/science/article/pii/S1342937X16301770
Society Cliffs, http://weblex.nrcan.gc.ca/html/014000/GSCC00053014034.html
from the 1.2 Ga Society Cliffs Formation of Baf<U+FB01>n Island (Hofmann and Jackson , 1991) .Canadian Arctic permineralizations show a similar curvature as the Farrel Quartzite spindles and some have <U+FB01>laments attached at one end .Eopoikilofusa is the most similar valid form genus to the Farrel Quartzite microfossil spindles .The Farrel Quartzite also contains numerous perfectly spherical opaque grains of pyrite , which are not microfossils , but likely framboids , and may represent results of microbial sulfur reduction (Sawlowicz , 1993) . Verified

Liu, Alexander G., 2016, Framboidal Pyrite Shroud Confirms The 'Death Mask' Model For Moldic Preservation Of Ediacaran Soft-Bodied Organisms, Palaios, 31, 5, 259--274, 10.2110/palo.2015.095, http://dx.doi.org/10.2110/palo.2015.095
Conception, http://weblex.nrcan.gc.ca/html/003000/GSCC00053003261.html
The mechanisms by which soft-bodied organisms were preserved in late Ediacaran deep-marine environments are revealed by petrographic and geochemical investigation of fossil-bearing surfaces from the Conception and St. John 's groups (Newfoundland , Canada) .Framboidal pyrite veneers are documented on fossilbearing horizons at multiple localities . Verified

Manual assessment of a random 5% sample

Manual assessment of pyrite framboid, concretion and nodule mentions (n = 123, representing a 5% random sample of all mentions, including stratgraphic packages/units of any age, and not excluding multiple mentions in one document)
117 verified hits
4 erroneous or false positive hits
2 unverified hits
95 % accuracy

Wilson, M.J.; Shaldybin, M.V.; Wilson, L., 2016, Clay mineralogy and unconventional hydrocarbon shale reservoirs in the USA. I. Occurrence and interpretation of mixed-layer R3 ordered illite/smectite, Earth-Science Reviews, , , 31--50, 10.1016/j.earscirev.2016.04.004, http://www.sciencedirect.com/science/article/pii/S0012825216300708
Utica, http://ngmdb.usgs.gov/Geolex/Units/Utica_4218.html
SEM images showing (a) lath-like illite growing in Utica shale and (b) Utica shale fabric showing an intimate mixture of platy and tiny lath-like particles (lower left corner) , quartz grains and pyrite framboids (lower right corner) (after Daniels et al. , 2011) Fig. 13 . Verified

REYNOLDS, RICHARD L.; FISHMAN, NEIL S.; WANTY, RICHARD B.; GOLDHABER, MARTIN B., 1990, Iron sulfide minerals at Cement oil field, Oklahoma: Implications for magnetic detection of oil fields, Geological Society of America Bulletin, 102, 3, 368--380, 10.1130/0016-7606(1990)102<0368:ismaco>2.3.co;2, http://dx.doi.org/10.1130/0016-7606(1990)102<0368:ismaco>2.3.co;2
Wellington, http://ngmdb.usgs.gov/Geolex/Units/Wellington_11096.html
The sulfide in the sample from well 9 (609.6-664 .6 m ; Wellington Formation) is isotopically light (-30.0 ° / oo) and consists partly of framboidal pyrite .The < 534S values of sulfide listed in Table 2 are very similar to the isotopic ratios (+10.1 to -9.4 per mil) obtained by Lilburn and Al-Shaieb (1984) from 30 samples from 16 other wells and from 2 pyrite nodules at the surface near the center of the Cement field (-9.3 and -12.0 per mil) . Verified

Slotznick, Sarah P.; Webb, Samuel M.; Kirschvink, Joseph L.; Fischer, Woodward W., NA, Mid-Proterozoic ferruginous conditions reflect post-depositional processes, Geophysical Research Letters, , , , 10.1029/2018GL081496, https://onlinelibrary.wiley.com/doi/abs/10.1029/2018GL081496#
Belt, http://ngmdb.usgs.gov/Geolex/Units/Belt_6823.html
To evaluate the mechanics of mid-Proterozoic environmental iron transport and deposition , we coupled microscale textural and bulk rock magnetic techniques to study the ~ 1.4 Ga lower Belt group , Belt Supergroup , Montana and Idaho .We identi<U+FB01>ed a pyrrhotite-siderite isograd that marks metamorphic iron-bearing mineral reactions beginning in subgreenschist facies samples .Even in the best-preserved parts of the basin , secondary overprints were common including recrystallization of iron-bearing sul<U+FB01>des , base metal sul<U+FB01>des , and nanophase pyrrhotite .Despite these overprints , a record of redox chemistry was preserved in the early diagenetic framboidal pyrite and detrital iron oxides including trace nanoscale magnetite that remained after sul<U+FB01>dization in anoxic and sul<U+FB01>dic sedimentary pore <U+FB02>uids Verified.

Grasby, Stephen E.; Beauchamp, Benoit, 2009, Latest Permian to Early Triassic basin-to-shelf anoxia in the Sverdrup Basin, Arctic Canada, Chemical Geology, 264, 1-4, 232--246, 10.1016/j.chemgeo.2009.03.009, http://www.sciencedirect.com/science/article/pii/S0009254109001181
Blind Fiord, http://weblex.nrcan.gc.ca/html/001000/GSCC00053001513.html
Onset of euxinic conditions (Fig. 8e) Elemental and isotopic geochemical evidence as well as the small size of abundant disseminated pyrite framboids indicates the onset of euxinic conditions as recorded some 1 -- 2 m below the base of the Blind Fiord Formation at Buchanan Lake . Verified [strictly unit below Blind Fiord]

Morin, R.H.; Sorey, M.L.; Jacobson, R.D., 1993, Results of the flowmeter-injection test in the Long Valley Exploratory Well (Phase II), Long Valley, California, Water-Resources Investigations Report, , , , 10.3133/wri934127, https://pubs.er.usgs.gov/publication/wri934127
Kincaid, http://ngmdb.usgs.gov/Geolex/Units/Kincaid_8852.html
Holt describes the Kincaid Formation as a glauconitic , sandy , greenish-gray shale overlain by an impure glauconitic , sandy , yellowish-gray limestone containing pyrite nodules . Verified

Hiatt, Eric E.; Pufahl, Peir K.; Edwards, Cole T., 2015, Sedimentary phosphate and associated fossil bacteria in a Paleoproterozoic tidal flat in the 1.85Ga Michigamme Formation, Michigan, USA, Sedimentary Geology, , , 24--39, 10.1016/j.sedgeo.2015.01.006, http://www.sciencedirect.com/science/article/pii/S003707381500038X
Michigamme, http://ngmdb.usgs.gov/Geolex/Units/Michigamme_2753.html
The Michigamme Formation accumulated near the end of the Earth 's initial phosphogenic episode (ca. 2.2 and 1.8 Ga) to produce one of the <U+FB01>rst granular phosphorites .Phosphatic lithofacies consist of <U+FB01>ne - to medium-sand-sized francolite peloids concentrated on bedding surfaces in peritidal facies .Granular beds are up to 2 cm thick and peloids are often partially to completely replaced by dolomite and chert .The grains contain organic matter and pyrite framboids that suggest bacterial breakdown of organic matter and bacterial sulfate reduction . Verified

Ardakani, Omid H.; Chappaz, Anthony; Sanei, Hamed; Mayer, Bernhard, 2016, Effect of thermal maturity on remobilization of molybdenum in black shales, Earth and Planetary Science Letters, , , 311--320, 10.1016/j.epsl.2016.06.004, http://www.sciencedirect.com/science/article/pii/S0012821X16302916
Utica, http://ngmdb.usgs.gov/Geolex/Units/Utica_4218.html
The breakdown of OM through thermal maturation in the deeper interval Utica Shale samples leads to deterioration of the Mo -- TOC correlation with depth (Fig. 2A , B , and C) .The Mo -- TOC correlation is affected in intervals where TSR coupled with OM oxidation occurs , and induces elevated Mo concentrations (Figs. 2B , C) .Pyrite recrystallization during TSR (Figs. 2B and C) likely causes Mo remobilization to pore <U+FB02>uids and the surrounding matrix (e.g. , Large et al. , 2007 ; Chappaz et al. , 2014 ; Gregory et al. , 2015) and , as a consequence , deteriorates the Mo -- S correlation (Fig. 2C) .Laser ablation-inductively coupled plasma-mass spectrometry (LA-ICP-MS) analysis on a large sample set of sedimentary pyrite (diagenetic and syngenetic) has shown that early formed framboidal pyrite has higher Mo concentrations than associated recrystallized pyrite in the same rock sample (Gregory et al. , 2015) . Verified [within paper]

Fisher, Donald M.; Brantley, Susan L., 1992, Models of quartz overgrowth and vein formation: Deformation and episodic fluid flow in an ancient subduction zone, Journal of Geophysical Research, 97, B13, 20043, 10.1029/92JB01582, http://doi.wiley.com/10.1029/92JB01582
Kodiak, http://ngmdb.usgs.gov/Geolex/Units/Kodiak_5802.html
For framboidal pyrite in the Kodiak Formation , the wavelength of irregularites along the growth interface is constant and largely reflects the pyrite grain size at the surfaceof the framboid (approximately0.5-2 gm , see Figure 2) . Verified

Cohee, George Vincent; West, Walter S.; Wilkie, Lorna C., 1967, Changes in stratigraphic nomenclature by the U.S. Geological Survey, 1966, Bulletin, , , , 10.3133/b1254A, https://pubs.er.usgs.gov/publication/b1254A
Exshaw, http://ngmdb.usgs.gov/Geolex/Units/Exshaw_8061.html
7) : Exshaw Formation : TLi .mest , one mem , ber : Thickness 3 .Limestone , black , argillaceous , quartzose , silty ; scattered pyrite nodules ; weathers rusty brown ; poorly preserved brachiopods at top of unit ; pelecypods , goniatites , orthoceratids , and trilobite in lower 2 ft _ _______________________ 37 Shale member : 2 . Verified

Edited by Tuttle, M. L., 1991, Geochemical, biogeochemical, and sedimentological studies of the Green River Formation, Wyoming, Utah, and Colorado, Bulletin, , , , , https://www.sciencebase.gov/catalog/item/4f4e4afde4b07f02db696e0c
Green River, http://ngmdb.usgs.gov/Geolex/Units/GreenRiver_8483.html
Framboidal pyrite almost always is found in organic stringers (filaments) in Green River Formation samples . Verified

Michel, F.A., 1986, Hydrogeology of the central Mackenzie Valley, Journal of Hydrology, 85, 3-4, 379--405, 10.1016/0022-1694(86)90068-5, http://www.sciencedirect.com/science/article/pii/0022169486900685
Canol, http://weblex.nrcan.gc.ca/html/002000/GSCC00053002271.html
In some areas the Canol Formation shales contain large pyrite nodules (Cook and Aitken , 1975) . Verified

Hudson, J. D.; Coleman, M. L.; Barreiro, B. A.; Hollingworth, N. T. J., 2001, Septarian concretions from the Oxford Clay (Jurassic, England, UK): involvement of original marine and multiple external pore fluids, Sedimentology, 48, 3, 507--531, 10.1046/j.1365-3091.2001.00374.x, http://doi.wiley.com/10.1046/j.1365-3091.2001.00374.x
Oxford Clay, http://www.bgs.ac.uk/lexicon/lexicon.cfm?pub=OXC
Like many other calcitic concretions (e.g. the Jet Rock , Coleman & Raiswell , 1995) , the Oxford Clay examples have rims that are strongly pyritic .This pyrite forms coalescent equant crystals , unlike the framboids which are dominant in the sediment outside the concretions .The pyritic rim is cut , in some cases , by septarian fractures which taper and do not extend to the margin of the concretion .In their inner parts , these cracks are themselves margined by thinner rims of pyrite , which permeates the adjacent concretion body , particularly affecting faecal pellets (Hudson , 1978 ; Figs 3 , 7A and B) . Verified

Smale, David; Mauk, Jeffrey L.; Palmer, Julie; Soong, Raymond; Blattner, Peter, 1999, Variations in sandstone diagenesis with depth, time, and space, onshore Taranaki wells, New Zealand, New Zealand Journal of Geology and Geophysics, 42, 2, 137--154, 10.1080/00288306.1999.9514836, http://www.tandfonline.com/doi/abs/10.1080/00288306.1999.9514836
Moki, NA
Framboidal pyrite from the Moki Formation in Kaimiro-2 is also later than the quartz overgrowths on which it has formed . Verified

Smale, David; Mauk, Jeffrey L.; Palmer, Julie; Soong, Raymond; Blattner, Peter, 1999, Variations in sandstone diagenesis with depth, time, and space, onshore Taranaki wells, New Zealand, New Zealand Journal of Geology and Geophysics, 42, 2, 137--154, 10.1080/00288306.1999.9514836, http://www.tandfonline.com/doi/abs/10.1080/00288306.1999.9514836
Moki, NA, Framboidal pyrite from the Moki Formation in Kaimiro-2 is also later than the quartz overgrowths on which it has formed . Verified

Tuttle, Michele L.; Goldhaber, Martin B., 1993, Sedimentary sulfur geochemistry of the Paleogene Green River Formation, western USA: Implications for interpreting depositional and diagenetic processes in saline alkaline lakes, Geochimica et Cosmochimica Acta, 57, 13, 3023--3039, 10.1016/0016-7037(93)90291-4, http://www.sciencedirect.com/science/article/pii/0016703793902914
Green River, http://ngmdb.usgs.gov/Geolex/Units/GreenRiver_8483.html
Photomicrographs showing typical sulfide-mineral morphology of the Green River Formation .(a) Pyrite framboids and framboids in which pyrite has filled spaces between pyrite crystals . Verified

Slotznick, Sarah P.; Webb, Samuel M.; Kirschvink, Joseph L.; Fischer, Woodward W., NA, Mid-Proterozoic ferruginous conditions reflect post-depositional processes, Geophysical Research Letters, , , , 10.1029/2018GL081496, https://onlinelibrary.wiley.com/doi/abs/10.1029/2018GL081496
Belt, http://ngmdb.usgs.gov/Geolex/Units/Belt_6823.html
Early diagenetic pyrite framboids and Fe-dolomite diagenetic cements in the lower Belt samples highlight the presence of anoxic and sulfidic pore fluid conditions ; these euxinic conditions potentially extended into deep portions of the water column in downdip settings in the Helena Embayment and further to the west . Verified

ZONNEVELD, J.-P.; BEATTY, T. W.; PEMBERTON, S. G., 2007, LINGULIDE BRACHIOPODS AND THE TRACE FOSSIL LINGULICHNUS FROM THE TRIASSIC OF WESTERN CANADA: IMPLICATIONS FOR FAUNAL RECOVERY AFTER THE END-PERMIAN MASS EXTINCTION, PALAIOS, 22, 1, 74--97, 10.2110/palo.2005.p05-103r, http://dx.doi.org/10.2110/palo.2005.p05-103r
Montney, http://weblex.nrcan.gc.ca/html/010000/GSCC00053010064.html
Abundant pyrite framboids and well-preserved palynomorphs with sulfide pseudomorphs on pollen and spore exines support an interpretation of anoxic-dysoxic conditions in proximal offshore-offshore transition successions in the Griesbachian sections of the lower Montney

Formation in northeastern British Columbia (Utting et al. , 2005 ; Zonneveld et al. , in press) . Verified

Loucks, R. G.; Reed, R. M.; Ruppel, S. C.; Jarvie, D. M., 2009, Morphology, Genesis, and Distribution of Nanometer-Scale Pores in Siliceous Mudstones of the Mississippian Barnett Shale, Journal of Sedimentary Research, 79, 12, 848--861, 10.2110/jsr.2009.092, http://dx.doi.org/10.2110/jsr.2009.092
Barnett, http://ngmdb.usgs.gov/Geolex/Units/Barnett_6685.html
We have now shown that naturally occurring pores in Barnett mudrocks are predominantly associated with organic matter and pyrite framboids .It should be noted , however , that even though mean pyrite abundance is high (average 9 %) , only framboidal pyrite commonly contains pores . Verified

Geldsetzer, Helmut H. J.; Goodfellow, Wayne D.; McLaren, Digby J.; Orchard, Mike J., 1987, Sulfur-isotope anomaly associated with the Frasnian-Famennian extinction, Medicine Lake, Alberta, Canada, Geol, 15, 5, 393, 10.1130/0091-7613(1987)15<393:saawtf>2.0.co;2, http://dx.doi.org/10.1130/0091-7613(1987)15<393:saawtf>2.0.co;2
Sassenach, http://weblex.nrcan.gc.ca/html/013000/GSCC00053013317.html
Thin section of basal Sassenach Formation .Dark grains along foresets are pyritic framboids . Verified

Kelly, William C.; Nishioka, Gail K., 1985, Precambrian oil inclusions in late veins and the role of hydrocarbons in copper mineralization at White Pine, Michigan, Geol, 13, 5, 334, 10.1130/0091-7613(1985)13<334:poiilv>2.0.co;2, http://dx.doi.org/10.1130/0091-7613(1985)13<334:poiilv>2.0.co;2
Nonesuch, http://ngmdb.usgs.gov/Geolex/Units/Nonesuch_3017.html
On the basis of abrupt disappearance of normal Nonesuch pyrite as shale beds enter the ore zone , and of the occurrence of some Cu-Fe sulfide pseudomorphs of pyrite framboids and euhedra within the nar - GEOLOGY , May 1985 335 row `` fringe , " Brown (1971) concluded that replacement of diagenetic pyrite was the principal copper control in the main ore zone . Verified

Carrigan, William J.; Cameron, Eion M., 1991, Petrological and stable isotope studies of carbonate and sulfide minerals from the Gunflint Formation, Ontario: evidence for the origin of early Proterozoic iron-formation, Precambrian Research, 52, 3-4, 347--380, 10.1016/0301-9268(91)90088-R, http://www.sciencedirect.com/science/article/pii/030192689190088R
Black, NA,
Black shales overlying the folded and brecciated beds contain three types of pyrite : fine-grained disseminated pyrite , coarse-grained pyrite , and pyrite concretions .Shale containing Types 1 and 2 have ~ 348 values between - 2 and + 35 % o that show an upward trend to heavier values .Pyrite concretions overgrow the first two types of pyrite , but formed during early diagenesis . Erroneous result

Weber, Bodo; Mota, Asdrulymar; Helenes, Javier; RamÃ-rez, Rafael; Valencia, Yoryi, 2016, Age and provenance of Late Miocene-Early Pliocene sedimentary rocks from the Patao high hydrocarbon reservoir offshore NE Venezuela â€ U-Pb detrital zircon age, Sm-Nd isotope, and biostratigraphic data, Journal of Natural Gas Science and Engineering, , , 459--473,

10.1016/j.jngse.2016.03.058,
http://www.sciencedirect.com/science/article/pii/S1875510016301652
Cubagua, NA
Deep-water sedimentary rocks with a uniform lithology of gray shale with abundant glauconite and pyrite nodules as well as sandy intervals that are intercalated with fine-grained clastic sedimentary rocks characterize the lower part of the Cubagua Formation . Verified

Morin, R.H.; Sorey, M.L.; Jacobson, R.D., 1993, Results of the flowmeter-injection test in the Long Valley Exploratory Well (Phase II), Long Valley, California, Water-Resources Investigations Report, , , , 10.3133/wri934127, https://pubs.er.usgs.gov/publication/wri934127
Kincaid, http://ngmdb.usgs.gov/Geolex/Units/Kincaid_8852.html
Holt describes the Kincaid Formation as a glauconitic , sandy , greenish-gray shale overlain by an impure glauconitic , sandy , yellowish-gray limestone containing pyrite nodules . Verified

Spinazola, Joseph M., 1993, Simulation of changes in water levels and ground-water flow in response to water-use alternatives in the Mud Lake area, eastern Snake River plain, eastern Idaho, Water-Resources Investigations Report, , , , 10.3133/wri934228,
https://pubs.er.usgs.gov/publication/wri934228
Kincaid, http://ngmdb.usgs.gov/Geolex/Units/Kincaid_8852.html
The Kincaid Formation is a glauconitic , sandy , greenish-gray shale overlain by an impure glauconitic , sandy , yellowish-gray limestone containing pyrite nodules . Verified

Ko, Lucy T.; Ruppel, Stephen C.; Loucks, Robert G.; Hackley, Paul C.; Zhang, Tongwei; Shao, Deyong, 2018, Pore-types and pore-network evolution in Upper Devonian-Lower Mississippian Woodford and Mississippian Barnett mudstones: Insights from laboratory thermal maturation and organic petrology, International Journal of Coal Geology, 190, , 3--28,
10.1016/j.coal.2017.10.001,
https://www.sciencedirect.com/science/article/pii/S0166516217303853
Woodford, NA
Original pore network (light blue) in the immature Woodford mudstone is predominantly composed of intraparticle pores between claymineral platelets , from dissolution of dolomite rims , and within pyrite framboids . Verified

Pates, Stephen; Daley, Allison C.; Lieberman, Bruce S., 2018, Hurdiid radiodontans from the middle Cambrian (Series 3) of Utah, Journal of Paleontology, 92, 01, 99--113,
10.1017/jpa.2017.11,
https://www.cambridge.org/core/product/identifier/S0022336017000117/type/journal_article
Pioche, http://ngmdb.usgs.gov/Geolex/Units/Pioche_6130.html
Similar structures , which were identified as clusters of pyrite framboids , have been reported from the middle Cambrian (Series 3) Pioche Shale by Moore and Lieberman (2009) . Verified

Clark, Sandra H.; Mosier, Elwin L., 1989, Barite nodules in Devonian shale and mudstone of western Virginia, Bulletin, , , , 10.3133/b1880, https://pubs.er.usgs.gov/publication/b1880
Millboro, http://ngmdb.usgs.gov/Geolex/Units/Millboro_2778.html
Although barite does not occur as discrete nodules at locality 8 , as at the other localities described , this locality is included because of the similarity in geologic setting to the other

localities in the Millboro Shale and because of the occurrence of barite within pyrite nodules .Equigranular baritic nodules occur south of McDowell (locality 9 , table 1 , fig. 1) but are rare .Baritic , calcitic , and pyritic nodules are in dark-gray to black shale that 6 Barite Nodules in Devonian Shale and Mudstone of Western Virginia Figure 5 . Verified

Harrison, R. W.; Litwin, R. J.; Repetski, J. E.; Mason, David; Schultz, A. P., 1996, Results of drilling in the English Hill area, Benton Hills, Scott County, Missouri, Open-File Report, , , , , https://www.sciencebase.gov/catalog/item/4f4e4a4ae4b07f02db62491a
Post Creek, http://ngmdb.usgs.gov/Geolex/Units/PostCreek_3384.html
pyrite nodule at 197.3 ft ; silt grains of dominantly clear quartz ------------------------------------ 194.8-198 .2 Post Creek Formation : Gravel , heterolithic clasts in a buff sandy matrix ; very coarse pebbles to cobbles ; subangular to subrounded ; clasts of light - 62 to dark-gray chert ; rounded to subrounded frosted quartz sand ------------------------ 198.2-205 .0 Sand and gravel , medium to dark gray ; about 50-50 % mixture ; massive , no apparent bedding ; subrounded to rounded , finegrained , well-sorted , frosted-quartz sand ; angular , matrix-supported , coarse - to finepebble , light - to dark-gray chert gravel ; gravel fines downward ; gray clay coats all clasts ; sparse pyrite ; common gypsum bloom - 205.0-212 .5 Clay , silt , and gravel , medium to dark gray ; lithologies similar to above , only finer grain sizes ; about 15 % fine-pebble to granule gravel , decreases in amount downward ; spotty very coarse pebble ; rounded , frosted-quartz silt ; common gypsum bloom -- 212.5-222 .0 Lower Cretaceous Series ? Unverified (unable to access original paper)

Prosser, D.J.; Daws, J.A.; Fallick, A.E.; Williams, B.P.J., 1994, THE OCCURRENCE AND Î´ 34 S OF AUTHIGENIC PYRITE IN MIDDLE JURASSIC BRENT GROUP SEDIMENTS, Journal of Petroleum Geology, 17, 4, 407--428, 10.1111/j.1747-5457.1994.tb00148.x, http://doi.wiley.com/10.1111/j.1747-5457.1994.tb00148.x
Brent, http://www.bgs.ac.uk/lexicon/lexicon.cfm?pub=BRNT
Illustration of some of the occurrences of pyrite within the Brent Group .A. Backscattered scanning electron image , illustrating framboidal pyrite (p) and clustered pyrite (c) associated with the replacement of plant dCbris within an organic-rich lamina .Note also scattered pyrite euhedra and zoned siderite cements (s) . Verified

Armstrong, Augustus K.; Bagby, W.C.; Ekburg, Charles; Repetski, John, 1987, Petrographic and scanning electron microscope studies of samples from the Roberts Mountains and Popovich formations, Carlin Mine area, Eureka County, Nevada, Bulletin, , , , 10.3133/b1684, https://pubs.er.usgs.gov/publication/b1684
Roberts Mountains, http://ngmdb.usgs.gov/Geolex/Units/RobertsMountains_6153.html
Roberts Mountains Formation : altered Carlin mine samples Samples from the Roberts Mountains Formation in the Carlin mine may be divided into the following categories : type I is dark-gray , organically rich arenaceous dolomite containing pyrite and illitic material ; type II is oxidized , light-yellow to orange and tan arenaceous rock composed of goethite-stained illitic material , quartz , and minor calcite ; and type III is oxidized , reddish-brown jasperoid .In hand specimens , type-1 rocks are medium-dark to dark gray and finely laminated ; they contain mud chips , worm burrows , and weakly developed cross laminations .These very subtle sedimentary structures are well preserved .Framboidal pyrite is preserved and common . Verified

Winter, Bryce L.; Knauth, L.Paul, 1992, Stable isotope geochemistry of early Protezozoic carbonate concretions in the Animikie group of the Lake Superior region: evidence for anaerobic bacterial processes, Precambrian Research, 54, 2-4, 131--151, 10.1016/0301-9268(92)90067-X, http://www.sciencedirect.com/science/article/pii/030192689290067X
Rove, http://ngmdb.usgs.gov/Geolex/Units/Rove_14330.html
The total ~ 345 range of + 6.5 TABLE 4 Sulfur isotopic composition and description of pyrite in carbonate concretions of the Rove Formation Sample No .d ; 34S Description Pyrite R2-12 R6-4 R6-5 R6-4 RI 1-4-5 R13-3-1 R13-3-2 R13-3-4 R13-3-6 R13-3-8 R13-3-9 RI5 R1-530 +14.3 +6.5 +6.8 +7.5 + 12.0 +9.9 +13.5 +11.4 +12.7 + 10.7 + 10.1 +7.0 +6.8 disseminated microscopic framboids euhedral pyrite (3 mm diameter) continuous lamina 10 cm spheriodal concretion core 1 mm cubes and disseminated framboids disseminated microscopic framboids 1mm cubes and disseminated framboids disseminated microscopic framboids disseminated microscopic framboids disseminated microscopic framboids disseminated microscopic framboids framboidal , concretion rim euhedral pyrite (3 mm diameter) Isotopic data given in per mil (% 0) . Verified

Schieber, J., 2011, Marcasite in Black Shales--a Mineral Proxy for Oxygenated Bottom Waters and Intermittent Oxidation of Carbonaceous Muds, Journal of Sedimentary Research, 81, 7, 447--458, 10.2110/jsr.2011.41, http://dx.doi.org/10.2110/jsr.2011.41
Powers Steps, http://weblex.nrcan.gc.ca/html/012000/GSCC00053012173.html
Carbonaceous shales of the Powers Steps Formation of Newfoundland show remnant spherical shapes that are interpreted as degraded pyrite framboids , pyrite patches , marcasite as euhedral equant grains or as overgrowth on pyrite framboids , differential compaction around marcasite-bearing regions , and a close spatial association between marcasite and early diagenetic quartz (Fig. 12) . Verified

Taylor, K.G.; Macquaker, J.H.S., 2000, Early diagenetic pyrite morphology in a mudstone-dominated succession: the Lower Jurassic Cleveland Ironstone Formation, eastern England, Sedimentary Geology, 131, 1-2, 77--86, 10.1016/S0037-0738(00)00002-6, http://www.sciencedirect.com/science/article/pii/S0037073800000026
Cleveland Ironstone, http://www.bgs.ac.uk/lexicon/lexicon.cfm?pub=CDI
Diagenetic pyrite in the mudstones and ironstones of the Lower Jurassic Cleveland Ironstone Formation of eastern England exhibits two distinct morphologies : framboidal pyrite , commonly associated with organic matter , and euhedral pyrite , associated with detrital clay pellets . Verified

Trewin, Nigel H; Fayers, Stephen R; Kelman, Ruth, 2003, Subaqueous silicification of the contents of small ponds in an Early Devonian hot-spring complex, Rhynie, Scotland, Canadian Journal of Earth Sciences, 40, 11, 1697--1712, 10.1139/e03-065, http://www.nrcresearchpress.com/doi/abs/10.1139/e03-065
Rhynie, http://www.bgs.ac.uk/lexicon/lexicon.cfm?pub=RHNC
In many samples from Rhynie , initial silica overlays appear to be contemporaneous with the precipitation of pyrite framboids , indicating at least mildly reducing pore-water conditions . Verified

Ardakani, Omid H.; Chappaz, Anthony; Sanei, Hamed; Mayer, Bernhard, 2016, Effect of thermal maturity on remobilization of molybdenum in black shales, Earth and Planetary Science

Letters, , , 311--320, 10.1016/j.epsl.2016.06.004, http://www.sciencedirect.com/science/article/pii/S0012821X16302916
Utica, http://ngmdb.usgs.gov/Geolex/Units/Utica_4218.html
The breakdown of OM through thermal maturation in the deeper interval Utica Shale samples leads to deterioration of the Mo -- TOC correlation with depth (Fig. 2A , B , and C) .The Mo -- TOC correlation is affected in intervals where TSR coupled with OM oxidation occurs , and induces elevated Mo concentrations (Figs. 2B , C) .Pyrite recrystallization during TSR (Figs. 2B and C) likely causes Mo remobilization to pore <U+FB02>uids and the surrounding matrix (e.g. , Large et al. , 2007 ; Chappaz et al. , 2014 ; Gregory et al. , 2015) and , as a consequence , deteriorates the Mo -- S correlation (Fig. 2C) .Laser ablation-inductively coupled plasma-mass spectrometry (LA-ICP-MS) analysis on a large sample set of sedimentary pyrite (diagenetic and syngenetic) has shown that early formed framboidal pyrite has higher Mo concentrations than associated recrystallized pyrite in the same rock sample (Gregory et al. , 2015) . Verified [within paper]

REYNOLDS, RICHARD L.; FISHMAN, NEIL S.; WANTY, RICHARD B.; GOLDHABER, MARTIN B., 1990, Iron sulfide minerals at Cement oil field, Oklahoma: Implications for magnetic detection of oil fields, Geological Society of America Bulletin, 102, 3, 368--380, 10.1130/0016-7606(1990)102<0368:ismaco>2.3.co;2, http://dx.doi.org/10.1130/0016-7606(1990)102<0368:ismaco>2.3.co;2
Wellington, http://ngmdb.usgs.gov/Geolex/Units/Wellington_11096.html
The sulfide in the sample from well 9 (609.6-664 .6 m ; Wellington Formation) is isotopically light (-30.0 ° / oo) and consists partly of framboidal pyrite . Verified

Carter, R.M., 1976, Stratigraphy of Maruia and Matiri Formations in their type section (Trent stream, Matiri river, Murchison), Journal of the Royal Society of New Zealand, 6, 4, 459--487, 10.1080/03036758.1976.10421485, http://www.tandfonline.com/doi/abs/10.1080/03036758.1976.10421485
Kaiata, NA
The main body of the siliceous concretions in the Kaiata Formation (OU 31182) is made up of a fine-grained mixture of fine clastic detritus (4-6 < 1 " micrite and microspar , and diagenetic groundmass quartz .Locally large amounts of framboidal pyrite may occur (up to 15 %) . Verified

Wignall, P.B., 2001, Sedimentology of the Triassic-Jurassic boundary beds in Pinhay Bay (Devon, SW England), Proceedings of the Geologists' Association, 112, 4, 349--360, 10.1016/S0016-7878(01)80014-6, http://www.sciencedirect.com/science/article/pii/S0016787801800146
Paper, NA
An unweathered block from the centre of the Paper Shale , collected from section 6 , was cut and polished and examined using the backscatter facility of a scanning electron microscope (SEM) .This enabled the size distribution of the abundant pyrite framboids in this lithology to be measured . Verified

REYNOLDS, RICHARD L.; FISHMAN, NEIL S.; WANTY, RICHARD B.; GOLDHABER, MARTIN B., 1990, Iron sulfide minerals at Cement oil field, Oklahoma: Implications for

magnetic detection of oil fields, Geological Society of America Bulletin, 102, 3, 368--380, 10.1130/0016-7606(1990)102<0368:ismaco>2.3.co;2, http://dx.doi.org/10.1130/0016-7606(1990)102<0368:ismaco>2.3.co;2
Wellington, http://ngmdb.usgs.gov/Geolex/Units/Wellington_11096.html
The sulfide in the sample from well 9 (609.6-664 .6 m ; Wellington Formation) is isotopically light (-30.0 ° / oo) and consists partly of framboidal pyrite .The < 534S values of sulfide listed in Table 2 are very similar to the isotopic ratios (+10.1 to -9.4 per mil) obtained by Lilburn and Al-Shaieb (1984) from 30 samples from 16 other wells and from 2 pyrite nodules at the surface near the center of the Cement field (-9.3 and -12.0 per mil) . Verified

FELDMANN, R. M.; FRANTESCU, A.; FRANTESCU, O. D.; KLOMPMAKER, A. A.; LOGAN, G.; ROBINS, C. M.; SCHWEITZER, C. E.; WAUGH, D. A., 2012, FORMATION OF LOBSTER-BEARING CONCRETIONS IN THE LATE CRETACEOUS BEARPAW SHALE, MONTANA, UNITED STATES, IN A COMPLEX GEOCHEMICAL ENVIRONMENT, PALAIOS, 27, 12, 842--856, 10.2110/palo.2012.p12-035r, http://dx.doi.org/10.2110/palo.2012.p12-035r
Bearpaw, http://weblex.nrcan.gc.ca/html/000000/GSCC00053000966.html
Eleven concretions containing the nephropid lobster , Palaeonephrops browni (Whitfield , 1907) , from the Upper Cretaceous (Campanian) , Bearpaw Formation in northeastern Montana , were examined using visual and geochemical methods .The concretions were zoned , with an axial , phosphate-rich core also containing calcium surrounding the lobster remains and an outer , calcium-rich zone lacking phosphate .The overall composition documents these as carbonate concretions , not phosphatic concretions .Where visible , the inner zone is sheathed in a thin layer dominated by framboidal pyrite , suggesting formation by a microbial film . Verified

Nelson, Gabriel J.; Pufahl, Peir K.; Hiatt, Eric E., 2010, Paleoceanographic constraints on Precambrian phosphorite accumulation, Baraga Group, Michigan, USA, Sedimentary Geology, 226, 1-4, 9--21, 10.1016/j.sedgeo.2010.02.001, http://www.sciencedirect.com/science/article/pii/S0037073810000321
Baraga, http://ngmdb.usgs.gov/Geolex/Units/Baraga_6671.html
Its highest concentration in the Baraga Group occurs within these organic-rich hemipelagites .Framboidal pyrite is much less abundant in prodelta siltstones (F4) and is absent in delta front deposits (F3) of the TST and HST .Such a marked difference in pyrite abundance between hemipelagites and G.J. Nelson et al. / Sedimentary Geology 226 (2010) 9 -- 21 17 Fig. 9 . Verified

Formolo, M. J.; Lyons, T. W., 2007, Accumulation and Preservation of Reworked Marine Pyrite Beneath an Oxygen-Rich Devonian Atmosphere: Constraints from Sulfur Isotopes and Framboid Textures, Journal of Sedimentary Research, 77, 8, 623--633, 10.2110/jsr.2007.062, http://dx.doi.org/10.2110/jsr.2007.062
Leicester Pyrite, http://ngmdb.usgs.gov/Geolex/Units/Leicester_13468.html
Because of the broad range of pyrite grain sizes observed locally in the Leicester Pyrite Member (from framboids to large nodules) , the absence of carbonate can not easily be explained by sorting of constituents with distinctively different hydrodynamic properties . Verified

Fraser, Tiffani A.; Hutchison, Matt P., 2017, Lithogeochemical characterization of the Middle-Upper Devonian Road River Group, Canol and Imperial formations on Trail River, east Richardson Mountains, Yukon: age constraints and a depositional model for fine-grained strata in the Lower Paleozoic Richardson trough, Canadian Journal of Earth Sciences, , , , 10.1139/cjes-2016-0216, http://www.nrcresearchpress.com/doi/10.1139/cjes-2016-0216
Canol, http://weblex.nrcan.gc.ca/html/002000/GSCC00053002271.html
Canol Formation chemozones C-G are all characterized by clearly defined cyclically decreasing/increasing major oxides (Al2O3 , TiO2 , K2O , Na2O) ; Zr , Th , and TIP values ; and increasing/decreasing SiO2 (except for LC-C which has a negative peak at 57m) ; U , Mo , V , SiO2/Zr ; efU ; efMo ; efV ; and Mo/TOC values .Except for LC-C , each basal chemozone boundary immediately precedes a positive excursion in d13Corg values followed by a decrease .All chemozones contain disseminated framboidal pyrite , and each chemozone boundary correlates to the more recessive siliceous shale or interbedded siliceous shale/chert lithofacies and in many cases to a locally higher TOC value (but not everywhere) . Verified

Ardakani, Omid H.; Chappaz, Anthony; Sanei, Hamed; Mayer, Bernhard, 2016, Effect of thermal maturity on remobilization of molybdenum in black shales, Earth and Planetary Science Letters, , , 311--320, 10.1016/j.epsl.2016.06.004, http://www.sciencedirect.com/science/article/pii/S0012821X16302916
Utica, http://ngmdb.usgs.gov/Geolex/Units/Utica_4218.html
Relationship between Mo concentration and d34S of pyrite in Utica Shale samples .The majority of samples associated with elevated Mo concentration and recrystallized pyrite in the intermediate (Mo > 3 ppm) and deep wells (Mo > 20 ppm) (broken-line box) have higher d34S values in comparison to those samples with low Mo concentration that are associated with framboidal pyrite (dotted line box) .The shaded area represents the assumed sulfur isotopic composition of Upper Ordovician seawater (after Kampschulte and Strauss , 2004) .Samples with recrystallized/coarse-grained pyrite show much less sulfur isotope fractionation between sulfate and sul<U+FB01>de compared to samples containing framboidal pyrite . Verified [within paper]

Lalonde, Stefan V.; Pecoits, Ernesto; von Gunten, Konstantin; Robbins, Leslie J.; Alessi, Daniel S.; Philippot, Pascal; Konhauser, Kurt O., 2018, Petrology and geochemistry of the Boolgeeda Iron Formation, Hamersley Basin, Western Australia, Precambrian Research, 316, , 155--173, 10.1016/j.precamres.2018.07.015, https://www.sciencedirect.com/science/article/pii/S0301926817306290
Boolgeeda Iron, http://dbforms.ga.gov.au/pls/www/geodx.strat_units.sch_full?wher=stratno=2227
Notably , martite and geothite are absent from thin sections , suggesting microplaty hematite within the Boolgeeda Iron Formation is not a paragenetic product of alteration typically associated with the formation of high grade hematite ore .Further , minnesotaite and stilpnomelane appear absent from thin section , attesting to the pristine nature of the iron bands .The prevalence of chlorite in the green mudstone/siltstones is likely the result of metamorphic alteration of primary clay material that composed these intervals .Philippot et al. (2018) documented the presence of multiple pyrite textures within TCDP1 , including nodular pyrite aggregates , clusters of microcrystalline pyrite , inclusion free pyrite overgrowths around pyrite framboids , <U+FB01>nely disseminated euhedral to subhedral pyrite crystals , euhedral to

subhedral pyrite crystals aligned with bedding and conforming to soft sediment deformation features , as well as bands of densely packed microcrystalline pyrite aggregates . Verified [via Philippot et al.]

Millward, David; Davies, Sarah J.; Williamson, Fiona; Curtis, Rachel; Kearsey, Timothy I.; Bennett, Carys E.; Marshall, John E. A.; Browne, Michael A. E., NA, Early Mississippian evaporites of coastal tropical wetlands, Sedimentology, , , , 10.1111/sed.12465, http://doi.wiley.com/10.1111/sed.12465
Ballagan, http://www.bgs.ac.uk/lexicon/lexicon.cfm?pub=BGN
Summary of the diagenesis of the evaporite rocks in the Ballagan Formation .References cited support the interpretation except for Scott (1986) and Kearsey et al. (2016) which are speci<U+FB01>c to these rocks .Phase Mineralogy & texture Where Timing and depth References Primary gypsum Gypsum dehydration to anhydrite Bacterial fermentation diagenesis Former presence inferred from lenticular pseudomorphs of aphanitic anhydrite Aphanitic anhydrite cores with wheatsheaf or fasciculate anhydrite surrounding ; granoblastic anhydrite .Poikilotopic anhydrite cement Laminae of dolomite crystals 8 to 25 lm containing thin layers of clay minerals , corroded quartz grains and associated clumps of pyrite framboids Hoddom : in evaporite laminites and sandy siltstone beds Norham : sporadic at margin of nodules Aphanitic in Hoddom and Norham ; granoblastic in Facies 2 Norham Putative microbial mats at base of Facies 2 in Norham Tournaisian ; primary sediment and in shallow subjacent sediment Tournaisian ; shallow subsurface Carboniferous , during burial 10 to 1000 m -- Shearman & Fuller (1969) ; Kasprzyk & Orti (1998) ; Aleali et al. (2013) Irwin (1980) Burial Rehydration secondary gypsum Dolomitization Calcitization Possible grain growth of granoblastic anhydrite , but otherwise little effect inferred ; granular anhydrite veins 1 . Verified

Clark, Sandra H.; Mosier, Elwin L., 1989, Barite nodules in Devonian shale and mudstone of western Virginia, Bulletin, , , , 10.3133/b1880, https://pubs.er.usgs.gov/publication/b1880
Millboro, http://ngmdb.usgs.gov/Geolex/Units/Millboro_2778.html
Pyritic nodules in which barite fills central fractures in the Millboro Shale near Flood (locality 8) .

Summary of lake stages represented by the samples in this study G4 Contents Petrography of Iron Sulfide Minerals in the Green River Formation of Wyoming , Utah , and Colorado By Michele L. Tuttle Abstract The Paleogene Green River Formation contains three iron sulfide minerals that are easily examined using reflectedlight microscopy : pyrite , pyrrhotite , and marcasite .At least four generations of pyrite were identified .Framboidal pyrite formed in organic stringers by the sulfidization of iron oxide minerals that coat clay minerals and were very reactive to H 2S (episode 1) .Euhedral and anhedral pyrite grains and pyrite infilling framboids formed when less reactive iron oxide minerals were dissolved , possibly during iron reduction by bacteria (episode 2) . Verified

Taylor, K.G.; Macquaker, J.H.S., 2000, Early diagenetic pyrite morphology in a mudstone-dominated succession: the Lower Jurassic Cleveland Ironstone Formation, eastern England, Sedimentary Geology, 131, 1-2, 77--86, 10.1016/S0037-0738(00)00002-6, http://www.sciencedirect.com/science/article/pii/S0037073800000026
Cleveland Ironstone, http://www.bgs.ac.uk/lexicon/lexicon.cfm?pub=CDI

Therefore , both pyrite morphologies in the Cleveland Ironstone Formation began to form during very early diagenesis .We , therefore , discount the possibility that framboidal pyrite formed during earliest diagenesis , and euhedral pyrite formed during later diagenesis .This assertion is further supported by the fact that the two pyrite morphologies are virtually always mutually exclusive in their occurrence . Verified

Armstrong, Joseph G.T.; Parnell, John; Bullock, Liam A.; Perez, Magali; Boyce, Adrian J.; Feldmann, Jorg, NA, Tellurium, selenium and cobalt enrichment in Neoproterozoic black shales, Gwna Group, UK: deep marine trace element enrichment during the Second Great Oxygenation Event, Terra Nova, , , , 10.1111/ter.12331, http://doi.wiley.com/10.1111/ter.12331
Gwna, http://www.bgs.ac.uk/lexicon/lexicon.cfm?pub=NGW
Framboids are 5 -- 10 lm in diameter and are disseminated throughout the Gwna Group black shale , while euhedral pyrites are generally larger (0.05 -- 2 mm) and present as discrete lenses within the unit (Figure 5b) .Clausthalite inclusions occur exclusively within the framboids , while Ni -- Co -- As phases are limited to the euhedral morphologies .Euhedral pyrites are observed to overgrow the framboids (Figure 5c) .LA-ICP-MS confirms that Se within the black shale occurs as evenly distributed , discrete PbSe phases , associated with micronscale pyrite , while Ni -- Co -- As mineralisation trends with larger pyrite (Figure 6) . Verified

Daws, J. A.; Prosser, D. J., 1992, SCALES OF PERMEABILITY HETEROGENEITY WITHIN THE BRENT GROUP, Journal of Petroleum Geology, 15, 3, 397--418, 10.1111/j.1747-5457.1992.tb00716.x, http://doi.wiley.com/10.1111/j.1747-5457.1992.tb00716.x
Rannoch, http://www.bgs.ac.uk/lexicon/lexicon.cfm?pub=RANN
The enhancement or occlusion of reservoir porosity changes the morphology and connectivity of the pore network , producing changes in rock permeability , e.g. carbonate-cemented horizons (doggers) within the Rannoch Formation cause complete occlusion of porosity and drastically reduce permeability (Fig. 3) .These are easily recognisable on wireline logs by the sharp increase in formation density (FDC) and decrease in neutron (CNL) log responses .Nodular pyrite cements form a further type of fifth-order heterogeneity encountered during this study . Verified

Faggetter, Luke E.; Wignall, Paul B.; Pruss, Sara B.; Newton, Robert J.; Sun, Yadong; Crowley, Stephen F., 2017, Trilobite extinctions, facies changes and the ROECE carbon isotope excursion at the Cambrian Series 2â€3 boundary, Great Basin, western USA, Palaeogeography, Palaeoclimatology, Palaeoecology, 478, , 53--66, 10.1016/j.palaeo.2017.04.009, https://www.sciencedirect.com/science/article/pii/S0031018217303668
Pioche, http://ngmdb.usgs.gov/Geolex/Units/Pioche_6130.html
Pyrite Framboid analysis Framboid size analysis was performed on the Series 2 -- Series 3 boundary strata (and thus the extinction horizon) from the Pioche Formation at Oak Springs Summit , where 11 samples were collected in a 7 m interval spanning 3.5 m either side of the extinction horizon .All samples contained abundant scattered crystals of pyrite ranging in size from 1 to 10 μm , often found agglomerated in clustered patches . Verified

Schieber, J.; Baird, G., 2001, On the Origin and Significance of Pyrite Spheres in Devonian Black Shales of North America, Journal of Sedimentary Research, 71, 1, 155--166, 10.1306/051600710155, http://dx.doi.org/10.1306/051600710155
Powers Steps, http://weblex.nrcan.gc.ca/html/012000/GSCC00053012173.html

Other examples occur in the Mid-Proterozoic Belt Series of Montana (Schieber 1985) , the Cambrian of Belgium (Love 1971) , the Early Ordovician Powers Steps Formation of Newfoundland (Ranger 1979 ; Schieber , unpublished data) , and the Triassic `` Rogenpyrit " of Germany (Fabricius 1961) .Finally , aggregates of framboids , irregular as well as spherical in shape , have been described from modern sediments by various authors (Bertolin et al. 1995 ; Bailey and Blackson 1984 ; Bailey , personal communication 1998) .Several of these are clearly framboid accumulations in cavities of organic remains (Kato 1967 ; Love 1969 ; Bailey and Blackson 1984) .Pending pyrite cementation , they represent potential pyrite spheres that could be reworked into lags as described from the Devonian sediments of this study . Verified

Carter, R.M., 1976, Stratigraphy of Maruia and Matiri Formations in their type section (Trent stream, Matiri river, Murchison), Journal of the Royal Society of New Zealand, 6, 4, 459--487, 10.1080/03036758.1976.10421485, http://www.tandfonline.com/doi/abs/10.1080/03036758.1976.10421485
Kaiata, NA
The main body of the siliceous concretions in the Kaiata Formation (OU 31182) is made up of a fine-grained mixture of fine clastic detritus (4-6 < 1 " micrite and microspar , and diagenetic groundmass quartz .Locally large amounts of framboidal pyrite may occur (up to 15 %) . Verified

John Bloch (2); H. Roy Krouse, 1992, Sulfide Diagenesis and Sedimentation in the Albian Harmon Member, Western Canada, SEPM Journal of Sedimentary Research, Vol. 62, , , 10.1306/d42678cf-2b26-11d7-8648000102c1865d, http://dx.doi.org/10.1306/d42678cf-2b26-11d7-8648000102c1865d
Harmon, http://weblex.nrcan.gc.ca/html/006000/GSCC00053006272.html
Direct precipitation of pyrite in the Harmon Member is inferred from the euhedral outer terminations of transition zone framboidal aggregates . Verified

Carter, R.M., 1976, Stratigraphy of Maruia and Matiri Formations in their type section (Trent stream, Matiri river, Murchison), Journal of the Royal Society of New Zealand, 6, 4, 459--487, 10.1080/03036758.1976.10421485, http://www.tandfonline.com/doi/abs/10.1080/03036758.1976.10421485
Kaiata, NA
The main body of the siliceous concretions in the Kaiata Formation (OU 31182) is made up of a fine-grained mixture of fine clastic detritus (4-6 < 1 " micrite and microspar , and diagenetic groundmass quartz .Locally large amounts of framboidal pyrite may occur (up to 15 %) . Verified

Park, John K., 1995, Paleomagnetism of the late Neoproterozoic Blueflower and Risky formations of the northern Cordillera, Canada, Canadian Journal of Earth Sciences, 32, 6, 718--729, 10.1139/e95-061, http://www.nrcresearchpress.com/doi/abs/10.1139/e95-061
Blueflower, http://weblex.nrcan.gc.ca/html/001000/GSCC00053001558.html
The Blueflower locality contains much pyrite , which occurs as single grains of cubic or framboidal aspect , and as overgrowths . Verified

Smale, David; Mauk, Jeffrey L.; Palmer, Julie; Soong, Raymond; Blattner, Peter, 1999, Variations in sandstone diagenesis with depth, time, and space, onshore Taranaki wells, New Zealand, New Zealand Journal of Geology and Geophysics, 42, 2, 137--154, 10.1080/00288306.1999.9514836, http://www.tandfonline.com/doi/abs/10.1080/00288306.1999.9514836
Moki, NA
Framboidal pyrite from the Moki Formation in Kaimiro-2 is also later than the quartz overgrowths on which it has formed . Verified

Allison, C.W., 1988, Paleontology of late Proterozoic and Early Cambrian rocks of east-central Alaska, Professional Paper, , , , 10.3133/pp1449, https://pubs.er.usgs.gov/publication/pp1449
Tindir, http://ngmdb.usgs.gov/Geolex/Units/Tindir_12100.html
Similarity of the pyrite-replaced multiunits described from these samples to pyrite framboids led Kalliokoski (1974) to interpret a nonbiologic origin for this Tindir material . Verified

Taylor, K.G.; Macquaker, J.H.S., 2000, Early diagenetic pyrite morphology in a mudstone-dominated succession: the Lower Jurassic Cleveland Ironstone Formation, eastern England, Sedimentary Geology, 131, 1-2, 77--86, 10.1016/S0037-0738(00)00002-6, http://www.sciencedirect.com/science/article/pii/S0037073800000026
Cleveland Ironstone, http://www.bgs.ac.uk/lexicon/lexicon.cfm?pub=CDI
We propose that the control on the pyrite morphology within the Cleveland Ironstone Formation mudstones was the rate of sul<U+FB01>de production during early diagenesis , which determined whether FeS or FeS_2 saturation was reached in early diagenetic porewaters .In clay-rich and silt-rich mudstones , containing framboidal pyrite , sul<U+FB01>de production rates were high as a result of low O_2 contents in depositional bottom waters and high organic matter reactivity , leading to high bacterial sulfate reduction rates . Verified

Fisher, Donald M.; Brantley, Susan L., 1992, Models of quartz overgrowth and vein formation: Deformation and episodic fluid flow in an ancient subduction zone, Journal of Geophysical Research, 97, B13, 20043, 10.1029/92JB01582, http://doi.wiley.com/10.1029/92JB01582
Kodiak, http://ngmdb.usgs.gov/Geolex/Units/Kodiak_5802.html
Displacement-ControlledFibrous Overgrowths Fibrous overgrowthsof quartz , chlorite , and phengite are distributed throughout the Kodiak Formation .Pressure shadows are observed around rigid objects such as spherical pyrite framboids (radius 10-100 gm) , fragments of metamorphosed organic material , and large (20-100 gm) detrital grains . Verified

Allison, Carol W.; Moorman, Mary A., 1974, Pyritized Microfossils and Pyrite Framboids: Reply, Geol, 2, 4, 202, 10.1130/0091-7613(1974)2<202:pmapfr>2.0.co;2, http://dx.doi.org/10.1130/0091-7613(1974)2<202:pmapfr>2.0.co;2
Tindir, http://ngmdb.usgs.gov/Geolex/Units/Tindir_12100.html
References Cited Allison , C. W. , and Moorman , M. A. , 1973 , Microbiota from the late Proterozoic Tindir Group , Alaska : Geology , v. 1 , no. 2 , p. 6 5 - 6 8 .Kalliokoski , J. , 1974 , Pyrite framboids : Animal , vegetable , or mineral ? Verified [see Emmings et al. 2019 RPP for examples of this texture]

Han, Kui; Ju, Yiwen; Wang, Guochang; Bao, Shujing; Bu, Hongling; Neupane, Bhupati, 2016, Shale composition and pore structure variations in the progradation direction: A case study of transitional shales in the Xu-Huai district, southern North China, Journal of Natural Gas Science and Engineering, 36, , 1178--1187, 10.1016/j.jngse.2016.03.022, http://www.sciencedirect.com/science/article/pii/S1875510016301287
Barnett, http://ngmdb.usgs.gov/Geolex/Units/Barnett_6685.html
The mineral compositions and characteristics vary with location , even within the same environment (Zhu et al. , 2012) , such as the variable biota and pyrite framboid yield in the Barnett shale in the Fort Worth Basin (Loucks and Ruppel , 2007) . Verified

Martinez R, J.I.; Hernandez, R., 1992, Evolution and drowning of the late cretaceous Venezuelan carbonate platform, Journal of South American Earth Sciences, 5, 2, 197--210, 10.1016/0895-9811(92)90038-Z, http://www.sciencedirect.com/science/article/pii/089598119290038Z
La Luna, NA
Excepting Inoceramas spp. , no other evidence of benthic life has been found in the La Luna Formation .The planktonic/benthonic ratio varies from 100 % at the base to ~ 95 % toward the top (Table 1) ; these figures , however , show wide regional variations .As authigenic minerals , silt-size framboidal pyrite is abundant , whereas silt-size glauconite occurs occasionally . Verified

Azaraien, Hassan; Shahabpour, Jamshid; Aminzadeh, Balandeh, 2017, Metallogenesis of the sediment-hosted stratiform Cu deposits of the Ravar Copper Belt (RCB), Central Iran, Ore Geology Reviews, 81, , 369--395, 10.1016/j.oregeorev.2016.09.035, http://www.sciencedirect.com/science/article/pii/S0169136816300440
Red, NA
Replacement , veinlet Unconformity type U deposits , IOCG deposits Chlorite , albitic , Fe calcite Faulting , algal mats , hydrocarbon fluids Presence intense Cu - Co-Ag-Au Hematite-Chalcocite-bornite-chalcopyritepyrite Red beds-evaporites Algal mats , bituminous , hydrocarbon Calcareous or dolomitic shale and siltstone Thin bedded stromatolites , fenestral structure cross bedding Permian Epi - continental shallow marine basins Red beds - ore bedevaporites -- dolomite and limestone Rift Chalcocite , bornite , covellite , pyrite , marcasite , argentite , spionkopite , malachite Calcite , quartz Finely dissemination , framboidal pyrite , colloform replacement veinlet Pb - Zn deposits , evaporites Chlorite Faulting , algal mats , marine black shale Presence Cu-Pb-Zn-Ag Hematite-Chalcocite-bornite-chalcopyritepyrite Red beds-evaporites Hydrocarbon , gas , black shale Sandstone , micro conglomerate and Siltstone Cross bedding , ripple mark Rip-up clast Upper Jurassic - Lower Cretaceous Fluvial and delta Evaporites -- red beds with interbeded of ore bed -- limestone Foreland basin Chalcocite , chalcopyrite , bornite , pyrite , carrollite , linaeit , Ge minerals , Co pyrite , azurite , malachite Calcite , quartz , anhydrite , dolomite Dissemination , replacement , veinlet lamination MVT deposits Bleaching , minor chlorite Woody fragments , permeability Presence Cu-Ag-U -- Red beds - organic matters , evaporites Plant fossils and woody fragments Fig. 25 . Erroneous result

Prosser, D.J.; Daws, J.A.; Fallick, A.E.; Williams, B.P.J., 1994, THE OCCURRENCE AND Î´ 34 S OF AUTHIGENIC PYRITE IN MIDDLE JURASSIC BRENT GROUP SEDIMENTS, Journal of Petroleum Geology, 17, 4, 407--428, 10.1111/j.1747-5457.1994.tb00148.x, http://doi.wiley.com/10.1111/j.1747-5457.1994.tb00148.x

Brent, http://www.bgs.ac.uk/lexicon/lexicon.cfm?pub=BRNT
More recently , Boyce et al. (1993) have documented very wide ranging 634Spyrite values (-27 to +71.7 % 0CDT) within Brent Group Sandstones using laser 634Smeasurementtechniques ; andMcConville etal .(1993) have shown that extreme variation in 634S (40 % 0) may occur at 100 pm sampling scale .Pyrite framboids , clusters , aggregates and finely-disseminated euhedra within shales , mudstones and siltstones display a wide range in 634S (-14.9 to +42.5 % 0CDT) , and are interpreted as including the earliest sulphide precipitates . Verified

John Bloch (2); H. Roy Krouse, 1992, Sulfide Diagenesis and Sedimentation in the Albian Harmon Member, Western Canada, SEPM Journal of Sedimentary Research, Vol. 62, , , 10.1306/d42678cf-2b26-11d7-8648000102c1865d, http://dx.doi.org/10.1306/d42678cf-2b26-11d7-8648000102c1865d
Harmon, http://weblex.nrcan.gc.ca/html/006000/GSCC00053006272.html
Direct precipitation of pyrite in the Harmon Member is inferred from the euhedral outer terminations of transition zone framboidal aggregates .These overgrowths preserve relict mackinawite or greigite morphology (Fig. 5) .The data presented in this study are consistent with previous work on the association of pyrite texture , isotopic composition and depositional processes and environments (Goldhaber and Kaplan 1974 ; Migdisov et al. 1974) .Raiswell (1982) correlated pyrite texture and isotopic composition to different chemical environments in the Lower Jurassic Jet Rock and concluded that later euhedral pyrite formation resulted from a lower degree of pyrite supersaturation and hence slower growth rate . Verified

Allison, C.W., 1988, Paleontology of late Proterozoic and Early Cambrian rocks of east-central Alaska, Professional Paper, , , , 10.3133/pp1449, https://pubs.er.usgs.gov/publication/pp1449
Tindir, http://ngmdb.usgs.gov/Geolex/Units/Tindir_12100.html
Similarity of the pyrite-replaced multiunits described from these samples to pyrite framboids led Kalliokoski (1974) to interpret a nonbiologic origin for this Tindir material .However , maceration of the samples , including treatment with nitric acid to remove the pyrite , yielded honeycomb-like membranous structures (Allison and Moorman 1973 , fig .IB) entirely comparable to the undoubtedly biogenic Sphaerocongregus multiunits in macerations of A-1408 (pi .14 , figs. 1 , 2) , which do not contain pyrite . Verified

Tosdal, Richard M., 1998, Contributions to the gold metallogeny of northern Nevada, Open-File Report, , , , 10.3133/ofr98338B, https://pubs.usgs.gov/of/1998/of98-338/
Roberts Mountains, http://ngmdb.usgs.gov/Geolex/Units/RobertsMountains_6153.html
Millimeter laminations are the most distinguishing characteristic of the Roberts Mountains Formation (Mullens , 1980) .SEM studies show the intercrystalline spaces contain sulfur-rich carbon , which is interpreted as derived from `` thermally altered " hydrocarbons .Environment ofDeposition .The presence of pyrite , in particular framboidal pyrite , and the preservation of the laminations indicates a reducing environment existed during deposition and was toxic to an infauna or boring organisms (Mullens , 1980) . Verified

Taylor, K.G.; Macquaker, J.H.S., 2000, Early diagenetic pyrite morphology in a mudstone-dominated succession: the Lower Jurassic Cleveland Ironstone Formation, eastern England, Sedimentary Geology, 131, 1-2, 77--86, 10.1016/S0037-0738(00)00002-6, http://www.sciencedirect.com/science/article/pii/S0037073800000026

Cleveland Ironstone, http://www.bgs.ac.uk/lexicon/lexicon.cfm?pub=CDI
Diagenetic pyrite in the mudstones and ironstones of the Lower Jurassic Cleveland Ironstone Formation of eastern England exhibits two distinct morphologies : framboidal pyrite , commonly associated with organic matter , and euhedral pyrite , associated with detrital clay pellets .
Verified

Edited by Tuttle, M. L., 1991, Geochemical, biogeochemical, and sedimentological studies of the Green River Formation, Wyoming, Utah, and Colorado, Bulletin, , , , , https://www.sciencebase.gov/catalog/item/4f4e4afde4b07f02db696e0c
Green River, http://ngmdb.usgs.gov/Geolex/Units/GreenRiver_8483.html
Framboidal pyrite almost always is found in organic stringers (filaments) in Green River Formation samples . Verified

Williford, Kenneth H.; Van Kranendonk, Martin J.; Ushikubo, Takayuki; Kozdon, Reinhard; Valley, John W., 2011, Constraining atmospheric oxygen and seawater sulfate concentrations during Paleoproterozoic glaciation: In situ sulfur three-isotope microanalysis of pyrite from the Turee Creek Group, Western Australia, Geochimica et Cosmochimica Acta, 75, 19, 5686--5705, 10.1016/j.gca.2011.07.010,
http://www.sciencedirect.com/science/article/pii/S0016703711003978
Marshall, http://ngmdb.usgs.gov/Geolex/Units/Marshall_2654.html
the Marshall Sandstone of southeastern Michigan , where As-rich (> 7 wt .% As) pyrite overgrowths have formed on As-poor framboidal pyrite , leading to As concentrations of up to 300 lg/L in local groundwater (Kolker and Nordstrom , 2001) . Verified

McCabe, Chad; Van der Voo, Rob; Peacor, Donald R.; Scotese, Christopher R.; Freeman, Roy, 1983, Diagenetic magnetite carries ancient yet secondary remanence in some Paleozoic sedimentary carbonates, Geol, 11, 4, 221, 10.1130/0091-7613(1983)11<221:dmcays>2.0.co;2,
http://dx.doi.org/10.1130/0091-7613(1983)11<221:dmcays>2.0.co;2
Bonneterre, http://ngmdb.usgs.gov/Geolex/Units/Bonneterre_556.html
Extracts from samples of the Bonneterre Formation also contain a b u n d a n t spherules , and E D A shows that only iron is present as a major or minor element .These spherules are very large , up to 150) im in diameter .They resemble diagenetic pyrite framboids that are also common in sedimentary carbonates . Ambiguous/erroneous result

Smale, David; Mauk, Jeffrey L.; Palmer, Julie; Soong, Raymond; Blattner, Peter, 1999, Variations in sandstone diagenesis with depth, time, and space, onshore Taranaki wells, New Zealand, New Zealand Journal of Geology and Geophysics, 42, 2, 137--154, 10.1080/00288306.1999.9514836,
http://www.tandfonline.com/doi/abs/10.1080/00288306.1999.9514836
Moki, NA
Framboidal pyrite from the Moki Formation in Kaimiro-2 is also later than the quartz overgrowths on which it has formed . Verified

Edited by Tuttle, M. L., 1991, Geochemical, biogeochemical, and sedimentological studies of the Green River Formation, Wyoming, Utah, and Colorado, Bulletin, , , , , https://www.sciencebase.gov/catalog/item/4f4e4afde4b07f02db696e0c

Green River, http://ngmdb.usgs.gov/Geolex/Units/GreenRiver_8483.html
Contemporaneous with formation of the earliest sulfide minerals (precursor FeS and framboidal pyrite) , organic matter in the Green River sediments was also sulfidized . Verified

O'BRIEN, LORNA J.; BRADDY, SIMON J.; RADLEY, JONATHAN D., 2009, A new arthropod resting trace and associated suite of trace fossils from the Lower Jurassic of Warwickshire, England, Palaeontology, 52, 5, 1099--1112, 10.1111/j.1475-4983.2009.00901.x, http://doi.wiley.com/10.1111/j.1475-4983.2009.00901.x
Saltford, http://www.bgs.ac.uk/lexicon/lexicon.cfm?pub=SASH
The horizon of interest for this study lies approximately 7 m above the base of the Saltford Shale Member where a concentration of calcareous siltstone lenticles representing shallow scour-<U+FB01>lls preserves the suite of trace fossils in hyporelief and epirelief .The lenticles are up to 205 mm long and 15 mm thick .The generally uneven , undulating , lower surfaces preserve shallow <U+FB02>ute casts and minute , disarticulated bivalve shells .Framboidal pyrite concretions are clustered on the lower surfaces of the lenticles . Verified

Loucks, R. G.; Reed, R. M.; Ruppel, S. C.; Jarvie, D. M., 2009, Morphology, Genesis, and Distribution of Nanometer-Scale Pores in Siliceous Mudstones of the Mississippian Barnett Shale, Journal of Sedimentary Research, 79, 12, 848--861, 10.2110/jsr.2009.092, http://dx.doi.org/10.2110/jsr.2009.092
Barnett, http://ngmdb.usgs.gov/Geolex/Units/Barnett_6685.html
Although fracture porosity has been proposed as a storage and transport mechanism for hydrocarbons in shales (e.g. , Dewhurst et al. 1999) , only one naturally occurring microfracture containing porosity has been found in the Barnett Shale , despite extensive searching using a variety of megascopic and microscopic investigative techniques .Cemented microfractures and fractures are present , however , particularly in carbonate-rich mudstones (Gale et al. 2007) .Micropores Most micropores are associated with whole microfossils , fragmentary fossil material , or pyrite framboids. Verified

Algeo, T.; Henderson, C. M.; Ellwood, B.; Rowe, H.; Elswick, E.; Bates, S.; Lyons, T.; Hower, J. C.; Smith, C.; Maynard, B.; Hays, L. E.; Summons, R. E.; Fulton, J.; Freeman, K. H., 2012, Evidence for a diachronous Late Permian marine crisis from the Canadian Arctic region, Geological Society of America Bulletin, 124, 9-10, 1424--1448, 10.1130/b30505.1, http://dx.doi.org/10.1130/b30505.1
Blind Fiord, http://weblex.nrcan.gc.ca/html/001000/GSCC00053001513.html
Total S concentrations in the Blind Fiord Formation are uniformly low (< 0.2 %) between the formation contact and the latest Permian mass extinction (5.6 -- 10.2 m) but rise to substantially higher values (mostly > 1.0 %) above the latest Permian mass extinction horizon (10.2 m) .More than 95 % of total S is pyrite S (data not shown) , and most of this S (especially in the high-S intervals) is `` excess S '' (Fig. 13A) , i.e. , S that exceeds the amount of reduced S expected relative to TOC for oxic-suboxic marine facies (Berner and Raiswell , 1983 ; Leventhal , 1983) .Petrographic study showed that pyrite was present both as irregular masses and framboids , and that pyrite was associated almost exclusively with organic clumps of probable marine origin (Fig. 11) .In contrast , organic macerals of undoubted terrestrial provenance show virtually no pyrite overgrowths . Verified

Fraser, Tiffani A.; Hutchison, Matt P., 2017, Lithogeochemical characterization of the Middle-Upper Devonian Road River Group, Canol and Imperial formations on Trail River, east Richardson Mountains, Yukon: age constraints and a depositional model for fine-grained strata in the Lower Paleozoic Richardson trough, Canadian Journal of Earth Sciences, , , , 10.1139/cjes-2016-0216, http://www.nrcresearchpress.com/doi/10.1139/cjes-2016-0216
Canol, http://weblex.nrcan.gc.ca/html/002000/GSCC00053002271.html
Canol Formation chemozones C-G are all characterized by clearly defined cyclically decreasing/increasing major oxides (Al2O3 , TiO2 , K2O , Na2O) ; Zr , Th , and TIP values ; and increasing/decreasing SiO2 (except for LC-C which has a negative peak at 57m) ; U , Mo , V , SiO2/Zr ; efU ; efMo ; efV ; and Mo/TOC values .Except for LC-C , each basal chemozone boundary immediately precedes a positive excursion in d13Corg values followed by a decrease .All chemozones contain disseminated framboidal pyrite , and each chemozone boundary correlates to the more recessive siliceous shale or interbedded siliceous shale/chert lithofacies and in many cases to a locally higher TOC value (but not everywhere) . Verified

Banerjee, Indranil; Ghosh, Santosh K.; Abercrombie, Hugh J.; Davies, Edward H., 1994, An integrated subsurface study of the Mannvilleâ€Colorado group boundary in the Cessford Field, Alberta, Canadian Journal of Earth Sciences, 31, 3, 489--504, 10.1139/e94-044, http://www.nrcresearchpress.com/doi/abs/10.1139/e94-044
Mannville, http://weblex.nrcan.gc.ca/html/009000/GSCC00053009141.html
Kaolinite is abundant , as in situ replacement through alteration of mica flakes to a mixture of kaolinite and illite is typical of Mannville sandstones , as is also the occurrence of dispersed framboidal pyrite crystal aggregates . Verified

Edited by Tuttle, M. L., 1991, Geochemical, biogeochemical, and sedimentological studies of the Green River Formation, Wyoming, Utah, and Colorado, Bulletin, , , , , https://www.sciencebase.gov/catalog/item/4f4e4afde4b07f02db696e0c
Green River, http://ngmdb.usgs.gov/Geolex/Units/GreenRiver_8483.html
Summary of lake stages represented by the samples in this study G4 Contents Petrography of Iron Sulfide Minerals in the Green River Formation of Wyoming , Utah , and Colorado By Michele L. Tuttle Abstract The Paleogene Green River Formation contains three iron sulfide minerals that are easily examined using reflectedlight microscopy : pyrite , pyrrhotite , and marcasite .At least four generations of pyrite were identified .Framboidal pyrite formed in organic stringers by the sulfidization of iron oxide minerals that coat clay minerals and were very reactive to H 2S (episode 1) .Euhedral and anhedral pyrite grains and pyrite infilling framboids formed when less reactive iron oxide minerals were dissolved , possibly during iron reduction by bacteria (episode 2) . Verified

Kabanov, Pavel; Gouwy, Sofie Annie, 2016, The Devonian Horn River Group and the basal Imperial Formation of the central Mackenzie Plain, N.W.T., Canada: Multiproxy stratigraphic framework of a black shale basin, Canadian Journal of Earth Sciences, , , , 10.1139/cjes-2016-0096, http://www.nrcresearchpress.com/doi/10.1139/cjes-2016-0096
Canol, http://weblex.nrcan.gc.ca/html/002000/GSCC00053002271.html
In new cores from the SOB area , the dominant facies of the Canol Formation is black laminated siliceous mudrock enriched in very fine grained (< 50µm) pyrite (Fig. 8A and 8B) .The latter is described at the hand lens scale as pyrite dust (evenly dispersed in rock matrix) , pyritic streaks

(laminar features laterally not exceeding 1 cm ; Fig. 8D) , and pyritic laminae (laminar features laterally approaching or exceeding core diameter) .These types of pyrite are regarded a matrix pyrite , in contrast to nodular pyrite that also occurs in plenty (Fig. 8E) . Verified

Kabanov, Pavel; Gouwy, Sofie Annie, 2016, The Devonian Horn River Group and the basal Imperial Formation of the central Mackenzie Plain, N.W.T., Canada: Multiproxy stratigraphic framework of a black shale basin, Canadian Journal of Earth Sciences, , , , 10.1139/cjes-2016-0096, http://www.nrcresearchpress.com/doi/10.1139/cjes-2016-0096
Canol, http://weblex.nrcan.gc.ca/html/002000/GSCC00053002271.html
In new cores from the SOB area , the dominant facies of the Canol Formation is black laminated siliceous mudrock enriched in very fine grained (< 50μm) pyrite (Fig. 8A and 8B) .The latter is described at the hand lens scale as pyrite dust (evenly dispersed in rock matrix) , pyritic streaks (laminar features laterally not exceeding 1 cm ; Fig. 8D) , and pyritic laminae (laminar features laterally approaching or exceeding core diameter) .These types of pyrite are regarded a matrix pyrite , in contrast to nodular pyrite that also occurs in plenty (Fig. 8E) .The matrix pyrite is likely composed of framboids diagnostic of anoxic sedimentary environments (Wilkin et al. 1996) . Verified

Ko, Lucy T.; Ruppel, Stephen C.; Loucks, Robert G.; Hackley, Paul C.; Zhang, Tongwei; Shao, Deyong, 2018, Pore-types and pore-network evolution in Upper Devonian-Lower Mississippian Woodford and Mississippian Barnett mudstones: Insights from laboratory thermal maturation and organic petrology, International Journal of Coal Geology, 190, , 3--28, 10.1016/j.coal.2017.10.001, https://www.sciencedirect.com/science/article/pii/S0166516217303853
Woodford, NA
Original pore network (light blue) in the immature Woodford mudstone is predominantly composed of intraparticle pores between claymineral platelets , from dissolution of dolomite rims , and within pyrite framboids . Verified

Taylor, K.G.; Macquaker, J.H.S., 2000, Early diagenetic pyrite morphology in a mudstone-dominated succession: the Lower Jurassic Cleveland Ironstone Formation, eastern England, Sedimentary Geology, 131, 1-2, 77--86, 10.1016/S0037-0738(00)00002-6, http://www.sciencedirect.com/science/article/pii/S0037073800000026
Cleveland Ironstone, http://www.bgs.ac.uk/lexicon/lexicon.cfm?pub=CDI
In this paper we show that within an ancient shallow marine mudstone succession (the Lower Jurassic Cleveland Ironstone Formation of eastern England) , early diagenetic pyrite morphology varies in a systematic manner .In some parts of the succession framboidal pyrite is the dominant morphology of early diagenetic pyrite , whereas in other parts of the succession euhedral pyrite is the dominant morphology of early diagenetic pyrite . Verified

Hieshima, G.B.; Pratt, L.M., 1991, Sulfur/carbon ratios and extractable organic matter of the middle proterozoic nonesuch formation, north american midcontinent rift, Precambrian Research, 54, 1, 65--79, 10.1016/0301-9268(91)90069-M, http://www.sciencedirect.com/science/article/pii/030192689190069M
Nonesuch, http://ngmdb.usgs.gov/Geolex/Units/Nonesuch_3017.html

The sulfur to carbon ratios and the intimate association fo framboidal pyrite and organic matter are suggested to he the result of bacterial sulfate reduction in the Nonesuch sediments . Verified

Allison, C.W., 1988, Paleontology of late Proterozoic and Early Cambrian rocks of east-central Alaska, Professional Paper, , , , 10.3133/pp1449, https://pubs.er.usgs.gov/publication/pp1449, Tindir, http://ngmdb.usgs.gov/Geolex/Units/Tindir_12100.html, Similarity of the pyrite-replaced multiunits described from these samples to pyrite framboids led Kalliokoski (1974) to interpret a nonbiologic origin for this Tindir material .

Ardakani, Omid H.; Chappaz, Anthony; Sanei, Hamed; Mayer, Bernhard, 2016, Effect of thermal maturity on remobilization of molybdenum in black shales, Earth and Planetary Science Letters, , , 311--320, 10.1016/j.epsl.2016.06.004, http://www.sciencedirect.com/science/article/pii/S0012821X16302916

Utica, http://ngmdb.usgs.gov/Geolex/Units/Utica_4218.html

All framboidal pyrite associated with low Mo contents within the Utica Shale was characterized by an average d34S value (Table 2) of - 34h (- 46.4 h to - 27.6 h ; Fig. 4) , more than 50h lower than the d34S value of the Upper Ordovi - O.H. Ardakani et al. / Earth and Planetary Science Letters 449 (2016) 311 -- 320 317 Fig. 3 . Verified

Clark, Sandra H.; Mosier, Elwin L., 1989, Barite nodules in Devonian shale and mudstone of western Virginia, Bulletin, , , , 10.3133/b1880, https://pubs.er.usgs.gov/publication/b1880

Millboro, http://ngmdb.usgs.gov/Geolex/Units/Millboro_2778.html

Although barite does not occur as discrete nodules at locality 8 , as at the other localities described , this locality is included because of the similarity in geologic setting to the other localities in the Millboro Shale and because of the occurrence of barite within pyrite nodules . Verified

Clark, Sandra H.; Mosier, Elwin L., 1989, Barite nodules in Devonian shale and mudstone of western Virginia, Bulletin, , , , 10.3133/b1880, https://pubs.er.usgs.gov/publication/b1880

Millboro, http://ngmdb.usgs.gov/Geolex/Units/Millboro_2778.html

Pyritic nodules in which barite fills central fractures in the Millboro Shale near Flood (locality 8) 9 8 . Verified

Cody, J.D; Hutcheon, I.e; Krouse, H.R, 1999, Fluid flow, mixing and the origin of CO2 and H2S by bacterial sulphate reduction in the Mannville Group, southern Alberta, Canada, Marine and Petroleum Geology, 16, 6, 495--510, 10.1016/S0264-8172(99)00012-4, http://www.sciencedirect.com/science/article/pii/S0264817299000124

Mannville, http://weblex.nrcan.gc.ca/html/009000/GSCC00053009141.html

bonates contain limited amounts of evaporites and could be a source of sulphate -LSB- Most Mannville Group sediments Fig -LSB- 6 -LSB- The d23S of `` a # H1S and `` b # SO3 compared to concentration of the associated SO3 -LSB- J -LSB- D -LSB- Cody et al -LSB- : Marine and Petroleum Geolo ` y 05 `` 0888 # 384Đ409 492 Fig -LSB- 7 -LSB- Photomicrographs of common pyrite textures observed in Mannville sediments -RSB- `` a # intergranular pyrite cements ^ `` b # large pyrite nodules ^ `` c# pyrite associated with detrital rock fragments ^ and `` d # pyrite associated with authigenic chlorite -LSB- isotopic fractionation from a limited reservoir of sulphate \ as suggested above \ and the anomalous iso ! Verified

McCabe, Chad; Van der Voo, Rob; Peacor, Donald R.; Scotese, Christopher R.; Freeman, Roy, 1983, Diagenetic magnetite carries ancient yet secondary remanence in some Paleozoic sedimentary carbonates, Geol, 11, 4, 221, 10.1130/0091-7613(1983)11<221:dmcays>2.0.co;2, http://dx.doi.org/10.1130/0091-7613(1983)11<221:dmcays>2.0.co;2
Bonneterre, http://ngmdb.usgs.gov/Geolex/Units/Bonneterre_556.html
Extracts from samples of the Bonneterre Formation also contain a b u n d a n t spherules , and E D A shows that only iron is present as a major or minor element .These spherules are very large , up to 150) im in diameter .They resemble diagenetic pyrite framboids that are also common in sedimentary carbonates . Ambiguous/erroneous result

Carter, R.M., 1976, Stratigraphy of Maruia and Matiri Formations in their type section (Trent stream, Matiri river, Murchison), Journal of the Royal Society of New Zealand, 6, 4, 459--487, 10.1080/03036758.1976.10421485, http://www.tandfonline.com/doi/abs/10.1080/03036758.1976.10421485
Kaiata, NA
A further major characteristic of lagoonal sediments is an unusual suite of diagenetic minerals , similar to that described under the petrography of Kaiata Formation .Significant points include : (1) the presence of abundant framboidal pyrite , formed from the reaction between natural iron salts and hydrogen sulphide produced by saprophytic bacteria ; (2) the absence of calcareous microfossils because of the dissolution of their tests in sulphuric acid produced by sulphur oxidising bacteria (e.g. Thiobacillus) ; (3) the presence of large septarian concretions that formed as a carbonate gel (shrinkage cracks) within the top few metres of sediment (thin sections of concretion centres show that porosity approaches that of the original sediment) , the carbonate for which probably derives from step 2 ; (4) the presence of small rhombs of siderite , often nucleating around a small oil-spot which may represent the residuum of the organic material that could have provided the necessary ferrous iron , while the carbonate again derives from step 2 ; (5) the presence of authigenic quartz , as replacement of calcite shell material and as a cement within the concretions that form around such shell fragments . Verified

Wilson, M.J.; Shaldybin, M.V.; Wilson, L., 2016, Clay mineralogy and unconventional hydrocarbon shale reservoirs in the USA. I. Occurrence and interpretation of mixed-layer R3 ordered illite/smectite, Earth-Science Reviews, , , 31--50, 10.1016/j.earscirev.2016.04.004, http://www.sciencedirect.com/science/article/pii/S0012825216300708
Utica, http://ngmdb.usgs.gov/Geolex/Units/Utica_4218.html
SEM images showing (a) lath-like illite growing in Utica shale and (b) Utica shale fabric showing an intimate mixture of platy and tiny lath-like particles (lower left corner) , quartz grains and pyrite framboids (lower right corner) (after Daniels et al. , 2011) Fig. 13 . Verified

Faggetter, Luke E.; Wignall, Paul B.; Pruss, Sara B.; Newton, Robert J.; Sun, Yadong; Crowley, Stephen F., 2017, Trilobite extinctions, facies changes and the ROECE carbon isotope excursion at the Cambrian Series 2â€3 boundary, Great Basin, western USA, Palaeogeography, Palaeoclimatology, Palaeoecology, 478, , 53--66, 10.1016/j.palaeo.2017.04.009, https://www.sciencedirect.com/science/article/pii/S0031018217303668
Pioche, http://ngmdb.usgs.gov/Geolex/Units/Pioche_6130.html
Seven samples were also analysed from the Pioche Formation at Ruin Wash where the olenellid extinction horizon has been located within a succession of marls (Palmer , 1998 ; Lieberman ,

2003 ; Fig .S1) .Generally , framboidal pyrite was absent at this location with the exception of two samples from 10 and 15 cm below the extinction horizon where they had size ranges that plot in the anoxic <U+FB01>eld (Fig. 8) . Verified

Ardakani, Omid H.; Chappaz, Anthony; Sanei, Hamed; Mayer, Bernhard, 2016, Effect of thermal maturity on remobilization of molybdenum in black shales, Earth and Planetary Science Letters, , , 311--320, 10.1016/j.epsl.2016.06.004,
http://www.sciencedirect.com/science/article/pii/S0012821X16302916
Utica, http://ngmdb.usgs.gov/Geolex/Units/Utica_4218.html
The breakdown of OM through thermal maturation in the deeper interval Utica Shale samples leads to deterioration of the Mo -- TOC correlation with depth (Fig. 2A , B , and C) .The Mo -- TOC correlation is affected in intervals where TSR coupled with OM oxidation occurs , and induces elevated Mo concentrations (Figs. 2B , C) .Pyrite recrystallization during TSR (Figs. 2B and C) likely causes Mo remobilization to pore <U+FB02>uids and the surrounding matrix (e.g. , Large et al. , 2007 ; Chappaz et al. , 2014 ; Gregory et al. , 2015) and , as a consequence , deteriorates the Mo -- S correlation (Fig. 2C) .Laser ablation-inductively coupled plasma-mass spectrometry (LA-ICP-MS) analysis on a large sample set of sedimentary pyrite (diagenetic and syngenetic) has shown that early formed framboidal pyrite has higher Mo concentrations than associated recrystallized pyrite in the same rock sample (Gregory et al. , 2015) . Verified

Moore, Rachel A.; Lieberman, Bruce S., 2009, Preservation of early and Middle Cambrian soft-bodied arthropods from the Pioche Shale, Nevada, USA, Palaeogeography, Palaeoclimatology, Palaeoecology, 277, 1-2, 57--62, 10.1016/j.palaeo.2009.02.014,
http://www.sciencedirect.com/science/article/pii/S0031018209000856
Pioche, http://ngmdb.usgs.gov/Geolex/Units/Pioche_6130.html
By contrast , the preservation of the soft-bodied fauna in the Middle Cambrian part of the Pioche Shale shows similarity to other softbodied faunas , in particular , the Early Cambrian Chengjiang biota .For example , both have soft tissues similarly replaced by framboidal and disseminated oxidized pyrite , which are also less frequently preserved as carbon <U+FB01>lms (Gabbott et al. , 2004 ; Zhu et al. , 2005) . Verified

Pates, Stephen; Daley, Allison C.; Lieberman, Bruce S., 2018, Hurdiid radiodontans from the middle Cambrian (Series 3) of Utah, Journal of Paleontology, 92, 01, 99--113, 10.1017/jpa.2017.11,
https://www.cambridge.org/core/product/identifier/S0022336017000117/type/journal_article
Pioche, http://ngmdb.usgs.gov/Geolex/Units/Pioche_6130.html
Similar structures , which were identified as clusters of pyrite framboids , have been reported from the middle Cambrian (Series 3) Pioche Shale by Moore and Lieberman (2009) . Verified

Grasby, Stephen E.; Beauchamp, Benoit, 2009, Latest Permian to Early Triassic basin-to-shelf anoxia in the Sverdrup Basin, Arctic Canada, Chemical Geology, 264, 1-4, 232--246, 10.1016/j.chemgeo.2009.03.009,
http://www.sciencedirect.com/science/article/pii/S0009254109001181
Blind Fiord, http://weblex.nrcan.gc.ca/html/001000/GSCC00053001513.html
Onset of euxinic conditions (Fig. 8e) Elemental and isotopic geochemical evidence as well as the small size of abundant disseminated pyrite framboids indicates the onset of euxinic conditions as

recorded some 1 -- 2 m below the base of the Blind Fiord Formation at Buchanan Lake .For euxinic conditions to exist , the Sverdrup Basin must have been strati<U+FB01>ed with water bodies of different densities -- an oxygen-rich upper layer and an oxygende<U+FB01>cient lower layer -- separated by a thermocline , picnocline and halocline impeding the downward diffusion of molecular oxygen .In the Black Sea , this transition occurs between 100 and 200 m (Ozsoy , and Unluata , 1997) .Pyrite forms through bacterial sulphate reduction at the base of the redox boundary and then rains down on the basin <U+FB02>oor . Verified [strictly just beneath Blind Fiord Fm]

LIU, ALEXANDER G., 2016, FRAMBOIDAL PYRITE SHROUD CONFIRMS THE â€˜DEATH MASK' MODEL FOR MOLDIC PRESERVATION OF EDIACARAN SOFT-BODIED ORGANISMS, PALAIOS, 31, 5, 259--274, 10.2110/palo.2015.095, http://dx.doi.org/10.2110/palo.2015.095
Conception, http://weblex.nrcan.gc.ca/html/003000/GSCC00053003261.html
The presence of pyrite in Newfoundland con<U+FB01>rms the applicability of the ` death mask ' model of Ediacaran taphonomy (cf. , Gehling 1999) to what has formerly been termed Conception-type preservation (cf. , Narbonne 2005) , although it is noted that it is the presence of sulfate reducing micro-organisms , and not their formation of laterally continuous microbial mats , which is essential to the process .Framboidal pyrite veneers at bedding interfaces in Newfoundland supplement observations of pyrite associated with Ediacaran fossils from numerous global localities and disparate facies and lithologies , including carbonates , shales , sandstones , and siltstones . Verified

Loucks, R. G.; Reed, R. M.; Ruppel, S. C.; Jarvie, D. M., 2009, Morphology, Genesis, and Distribution of Nanometer-Scale Pores in Siliceous Mudstones of the Mississippian Barnett Shale, Journal of Sedimentary Research, 79, 12, 848--861, 10.2110/jsr.2009.092, http://dx.doi.org/10.2110/jsr.2009.092
Barnett, http://ngmdb.usgs.gov/Geolex/Units/Barnett_6685.html
We have now shown that naturally occurring pores in Barnett mudrocks are predominantly associated with organic matter and pyrite framboids .It should be noted , however , that even though mean pyrite abundance is high (average 9 %) , only framboidal pyrite commonly contains pores . Verified

Faggetter, Luke E.; Wignall, Paul B.; Pruss, Sara B.; Newton, Robert J.; Sun, Yadong; Crowley, Stephen F., 2017, Trilobite extinctions, facies changes and the ROECE carbon isotope excursion at the Cambrian Series 2â€3 boundary, Great Basin, western USA, Palaeogeography, Palaeoclimatology, Palaeoecology, 478, , 53--66, 10.1016/j.palaeo.2017.04.009, https://www.sciencedirect.com/science/article/pii/S0031018217303668
Carrara, http://ngmdb.usgs.gov/Geolex/Units/Carrara_4932.html
This level lies around 45 m above ROECE in the Carrara Formation .Rather than regression , the olenellid extinction occurs within a deepening succession .Transgression and shelf anoxia often go handin-hand , and oxygen stress has been implicated in ROECE extinction (Montañez et al. , 2000) .However , at Oak Springs Summit , pyrite framboid analysis suggests dysoxic but not euxinic conditions in the extinction interval , and the shallower study locations show no evidence for oxygen restriction . Verified

Rogers, Karyne M.; Morgans, Hugh E.G.; Wilson, Gary S., 2001, Identification of a Waipawa Formation equivalent in the upper Te Uri Member of the Whangai Formation â€� implications for depositional history and age, New Zealand Journal of Geology and Geophysics, 44, 2, 347--354, 10.1080/00288306.2001.9514943, http://www.tandfonline.com/doi/abs/10.1080/00288306.2001.9514943
Wanstead, NA
Thin glauconitic beds , concretions , and pyrite nodules mark bedding within the Wanstead Formation . Verified

Ko, Lucy T.; Ruppel, Stephen C.; Loucks, Robert G.; Hackley, Paul C.; Zhang, Tongwei; Shao, Deyong, 2018, Pore-types and pore-network evolution in Upper Devonian-Lower Mississippian Woodford and Mississippian Barnett mudstones: Insights from laboratory thermal maturation and organic petrology, International Journal of Coal Geology, 190, , 3--28, 10.1016/j.coal.2017.10.001, https://www.sciencedirect.com/science/article/pii/S0166516217303853
Woodford, NA
Original pore network (light blue) in the immature Woodford mudstone is predominantly composed of intraparticle pores between claymineral platelets , from dissolution of dolomite rims , and within pyrite framboids . Verified

Hiatt, Eric E.; Pufahl, Peir K.; Edwards, Cole T., 2015, Sedimentary phosphate and associated fossil bacteria in a Paleoproterozoic tidal flat in the 1.85Ga Michigamme Formation, Michigan, USA, Sedimentary Geology, , , 24--39, 10.1016/j.sedgeo.2015.01.006, http://www.sciencedirect.com/science/article/pii/S003707381500038X
Michigamme, http://ngmdb.usgs.gov/Geolex/Units/Michigamme_2753.html
The Michigamme Formation accumulated near the end of the Earth 's initial phosphogenic episode (ca. 2.2 and 1.8 Ga) to produce one of the <U+FB01>rst granular phosphorites .Phosphatic lithofacies consist of <U+FB01>ne - to medium-sand-sized francolite peloids concentrated on bedding surfaces in peritidal facies .Granular beds are up to 2 cm thick and peloids are often partially to completely replaced by dolomite and chert .The grains contain organic matter and pyrite framboids that suggest bacterial breakdown of organic matter and bacterial sulfate reduction . Verified

Haruna, Makoto; Hanamuro, Takahiro; Uyeda, Kaoru; Fujimaki, Hirokazu; Ohmoto, Hiroshi, 2003, Chemical, Isotopic, and Fluid Inclusion Evidence for the Hydrothermal Alteration of the Footwall Rocks of the BIF-Hosted Iron Ore Deposits in the Hamersley District, Western Australia, Resource Geology, 53, 2, 75--88, 10.1111/j.1751-3928.2003.tb00160.x, http://doi.wiley.com/10.1111/j.1751-3928.2003.tb00160.x
Mount McRae, http://dbforms.ga.gov.au/pls/www/geodx.strat_units.sch_full?wher=stratno=12926
The unusually Fig. 3 Bulk chemical compositions of the Mount McRae Shale .The low Na and Ca contents are related to the absence or extremely low modal abun - chemical compositions of the average terrigenous shale (Taylor and McLennan , 1985) are shown by arrows for comparison .dances of both plagioclase and calcite , which are the most common Na and/or Ca minerals in pyrite and pyrite nodules . Verified

Fraser, Tiffani A.; Hutchison, Matt P., 2017, Lithogeochemical characterization of the Middle-Upper Devonian Road River Group, Canol and Imperial formations on Trail River, east Richardson Mountains, Yukon: age constraints and a depositional model for fine-grained strata in the Lower Paleozoic Richardson trough, Canadian Journal of Earth Sciences, , , , 10.1139/cjes-2016-0216, http://www.nrcresearchpress.com/doi/10.1139/cjes-2016-0216
Canol, http://weblex.nrcan.gc.ca/html/002000/GSCC00053002271.html
Canol Formation chemozones C-G are all characterized by clearly defined cyclically decreasing/increasing major oxides (Al2O3 , TiO2 , K2O , Na2O) ; Zr , Th , and TIP values ; and increasing/decreasing SiO2 (except for LC-C which has a negative peak at 57m) ; U , Mo , V , SiO2/Zr ; efU ; efMo ; efV ; and Mo/TOC values .Except for LC-C , each basal chemozone boundary immediately precedes a positive excursion in d13Corg values followed by a decrease .All chemozones contain disseminated framboidal pyrite , and each chemozone boundary correlates to the more recessive siliceous shale or interbedded siliceous shale/chert lithofacies and in many cases to a locally higher TOC value (but not everywhere) . Verified

Loucks, R. G.; Reed, R. M.; Ruppel, S. C.; Jarvie, D. M., 2009, Morphology, Genesis, and Distribution of Nanometer-Scale Pores in Siliceous Mudstones of the Mississippian Barnett Shale, Journal of Sedimentary Research, 79, 12, 848--861, 10.2110/jsr.2009.092, http://dx.doi.org/10.2110/jsr.2009.092
Barnett, http://ngmdb.usgs.gov/Geolex/Units/Barnett_6685.html
We have now shown that naturally occurring pores in Barnett mudrocks are predominantly associated with organic matter and pyrite framboids .It should be noted , however , that even though mean pyrite abundance is high (average 9 %) , only framboidal pyrite commonly contains pores . Verified

White, Timothy S.; Witzke, Brian J.; Ludvigson, Gregory A., 2000, Evidence for an Albian Hudson arm connection between the Cretaceous Western Interior Seaway of North America and the Labrador Sea, Geological Society of America Bulletin, 112, 9, 1342--1355, 10.1130/0016-7606(2000)112<1342:efaaha>2.0.co;2, http://dx.doi.org/10.1130/0016-7606(2000)112<1342:efaaha>2.0.co;2
Mattagami, http://weblex.nrcan.gc.ca/html/009000/GSCC00053009324.html
Wilkin and Barnes (1997) demonstrated that framboids form in the water column of the Pettaquamscutt River estuary , and subsequently settle to the sediment-water interface , so the presence of framboids further suggests that much of the pyrite in the Mattagami Formation formed during deposition .Carruccio et al. (1977) reported a direct correlation between overlying marine strata and the presence of framboids in coal , and White et al. (1990) found that extant marsh chemistries have an overprinting effect on underlying sediments producing relatively high amounts of pyrite beneath a marsh . Verified

Goodfellow, W.D.; Jonasson, I.R.; Morganti, J.M., 1983, Zonation of chalcophile elements about the howard's pass (XY) Zn-Pb deposit, Selwyn Basin, Yukon, Journal of Geochemical Exploration, 19, 1-3, 503--542, 10.1016/0375-6742(83)90044-4, http://www.sciencedirect.com/science/article/pii/0375674283900444
Active, NA
(5) Photomicrograph illustrating the relationship between framboidal pyrite , organic matter , sphalerite (Sph) and galena (Gn) in the Active Member . Verified

Partridge, Michaela A.; Golding, Suzanne D.; Baublys, Kim A.; Young, Elisa, 2008, Pyrite paragenesis and multiple sulfur isotope distribution in late Archean and early Paleoproterozoic Hamersley Basin sediments, Earth and Planetary Science Letters, 272, 1-2, 41--49, 10.1016/j.epsl.2008.03.051, http://www.sciencedirect.com/science/article/pii/S0012821X08002318
Wittenoom, http://dbforms.ga.gov.au/pls/www/geodx.strat_units.sch_full?wher=stratno=20327
As is the case with the deeper water Marra Mamba Iron Formation , pyrite nodules in the shallower water Wittenoom Formation reveal a different sulfur source to that of fine grained pyrite . Verified

Formolo, M. J.; Lyons, T. W., 2007, Accumulation and Preservation of Reworked Marine Pyrite Beneath an Oxygen-Rich Devonian Atmosphere: Constraints from Sulfur Isotopes and Framboid Textures, Journal of Sedimentary Research, 77, 8, 623--633, 10.2110/jsr.2007.062, http://dx.doi.org/10.2110/jsr.2007.062
Leicester Pyrite, http://ngmdb.usgs.gov/Geolex/Units/Leicester_13468.html
These questions are addressed through geochemical and petrographic methods , specifically sulfur isotope compositions and framboidal pyrite distributions within the Leicester Pyrite Member and the overlying and underlying enclosing shales .These questions become particularly relevant in light of their paleoredox significance throughout the geologic record .Detrital pyrite is commonly diagnostic of pre-2 .4 billion year old Precambrian fluvial channel deposits and as such is one of our principal fingerprints of a low O2 atmosphere during Earth 's earliest history (Ramdohr 1958 ; Schidlowski 1981 ; Rasmussen and Buick 1999) . Verified

Kster, J.; Littke, R.; Machel, H. G., 2008, DEVONIAN CARBONATES OF THE NIGEL PEAK AREA, ROCKY MOUNTAINS, CANADA: A FOSSIL PETROLEUM SYSTEM, Journal of Petroleum Geology, 31, 3, 283--301, 10.1111/j.1747-5457.2008.00421.x, http://doi.wiley.com/10.1111/j.1747-5457.2008.00421.x
Perdrix, http://weblex.nrcan.gc.ca/html/011000/GSCC00053011713.html
The first is dispersed throughout the matrix forming a network of either solid bitumen or altered kerogen , or in the Perdrix Formation as elongate stringers (Fig. 9A) , sometimes in association with abundant , early diagenetic , framboidal pyrite . Verified

King, Philip Burke; Henbest, Lloyd G.; Yochelson, E.L.; Cloud, P.E.; Duncan, Helen; Finks, R.M.; Sohn, I.G., 1965, Geology of the Sierra Diablo region, Texas, with special determinative studies of Permian fossils, Professional Paper, , , , 10.3133/pp480, http://ngmdb.usgs.gov/Prodesc/proddesc_4485.htm
Barnett, http://ngmdb.usgs.gov/Geolex/Units/Barnett_6685.html
LITHOLOGY AND THICKNESS The Barnett is black , purplish , or dark-gray , carbonaceous shale , which contains small phosphatic and pyritic nodules and lenses and beds of earthy limestone . Verified

Steadman, Jeffrey A.; Large, Ross R.; Davidson, Garry J.; Bull, Stuart W.; Thompson, Jay; Ireland, Trevor R.; Holden, Peter, 2014, Paragenesis and composition of ore minerals in the Randalls BIF-hosted gold deposits, Yilgarn Craton, Western Australia: Implications for the timing of deposit formation and constraints on gold sources, Precambrian Research, , , 110--132,

10.1016/j.precamres.2014.01.002,
http://www.sciencedirect.com/science/article/pii/S0301926814000217
Black Flag, http://dbforms.ga.gov.au/pls/www/geodx.strat_units.sch_full?wher=stratno=28372
Following uplift , erosion and folding associated with regional D2 compression and low-grade regional metamorphism , the upper Black Flag Group was buried during Belches Supersequence sedimentation , and then metamorphosed again by the intrusive post-Belches granite plutons .The metamorphism associated with granite intrusion may have facilitated pyrite nodule breakdown (to pyrrhotite) and release of trace elements , especially As and Au , to the metamorphic/hydrothermal <U+FB02>uid (Large et al. , 2011) . Verified

Tuttle, Michele L.; Goldhaber, Martin B., 1993, Sedimentary sulfur geochemistry of the Paleogene Green River Formation, western USA: Implications for interpreting depositional and diagenetic processes in saline alkaline lakes, Geochimica et Cosmochimica Acta, 57, 13, 3023--3039, 10.1016/0016-7037(93)90291-4,
http://www.sciencedirect.com/science/article/pii/0016703793902914
Green River, http://ngmdb.usgs.gov/Geolex/Units/GreenRiver_8483.html
Photomicrographs showing typical sulfide-mineral morphology of the Green River Formation .(a) Pyrite framboids and framboids in which pyrite has filled spaces between pyrite crystals .
Verified

Edited by Tuttle, M. L., 1991, Geochemical, biogeochemical, and sedimentological studies of the Green River Formation, Wyoming, Utah, and Colorado, Bulletin, , , , ,
https://www.sciencebase.gov/catalog/item/4f4e4afde4b07f02db696e0c
Green River, http://ngmdb.usgs.gov/Geolex/Units/GreenRiver_8483.html
Summary of lake stages represented by the samples in this study G4 Contents Petrography of Iron Sulfide Minerals in the Green River Formation of Wyoming , Utah , and Colorado By Michele L. Tuttle Abstract The Paleogene Green River Formation contains three iron sulfide minerals that are easily examined using reflectedlight microscopy : pyrite , pyrrhotite , and marcasite .At least four generations of pyrite were identified .Framboidal pyrite formed in organic stringers by the sulfidization of iron oxide minerals that coat clay minerals and were very reactive to H 2S (episode 1) . Verified

Fraser, Tiffani A.; Hutchison, Matt P., 2017, Lithogeochemical characterization of the Middle-Upper Devonian Road River Group, Canol and Imperial formations on Trail River, east Richardson Mountains, Yukon: age constraints and a depositional model for fine-grained strata in the Lower Paleozoic Richardson trough, Canadian Journal of Earth Sciences, , , ,
10.1139/cjes-2016-0216, http://www.nrcresearchpress.com/doi/10.1139/cjes-2016-0216
Canol, http://weblex.nrcan.gc.ca/html/002000/GSCC00053002271.html
The average TOC is 1.7 wt % ; LC - C (19.7-83 .0 m) and LC-D (83.0-145 .0 m) represent the basal two Canol cycles and are characterized by a dominance of chert with minor siliceous shale and pyritic concretions . Verified

Retallack, Gregory J., 2014, Volcanosedimentary paleoenvironments of Ediacaran fossils in Newfoundland, Geological Society of America Bulletin, 126, 5-6, 619--638, 10.1130/b30892.1,
http://dx.doi.org/10.1130/b30892.1
Mistaken Point, http://weblex.nrcan.gc.ca/html/009000/GSCC00053009936.html

Measured sections of Mistaken Point Formation , Newfoundland .25 20 10 5 60 v a a a a av 50 a a 40 a a a a v 30 a a v v a 20 aa a a 10 a R3925-9 R39230-4 R3935-53 a 0 v R4075-9 D. Green Head m. 4 a S-J 2 v S-I 0 gsraasiclvlnteadly R4211 v KEY volcanic tuff sandstone S-H siltstone , claystone red claystone breccia grey claystone breccia volcanic lapilli basaltic scoria bomb a volcanic spindle bomb gray color red color aa brown stained layers S-G pyrite nodules v planar bedding R4011-23 flaser bedding v hummocky cross stratification trough cross bedding ripple marks S-F scour-and-fill slump bedding S-E clastic dykes a discoid fossils S-D (Aspidella , Heimalora) a v frond fossils (Fractifusus , Charniodiscus) a t tilting traces (trails ? Verified

Lohr, S.C.; Baruch, E.T.; Hall, P.A.; Kennedy, M.J., 2015, Is organic pore development in gas shales influenced by the primary porosity and structure of thermally immature organic matter?, Organic Geochemistry, , , 119--132, 10.1016/j.orggeochem.2015.07.010, http://www.sciencedirect.com/science/article/pii/S0146638015001527
Stuart Range, http://dbforms.ga.gov.au/pls/www/geodx.strat_units.sch_full?wher=stratno=17451
The Stuart Range Formation (Permo-Carboniferious Arckaringa Basin , central-northern South Australia) is an organic-rich mudrock comprising chemically immature , coarse silt mineralogy associated with the end-Permian deglaciation (Menpes , 2012) and abundant (up to 30 wt %) framboidal pyrite . Verified

Carter, R.M., 1976, Stratigraphy of Maruia and Matiri Formations in their type section (Trent stream, Matiri river, Murchison), Journal of the Royal Society of New Zealand, 6, 4, 459--487, 10.1080/03036758.1976.10421485, http://www.tandfonline.com/doi/abs/10.1080/03036758.1976.10421485
Kaiata, NA
A further major characteristic of lagoonal sediments is an unusual suite of diagenetic minerals , similar to that described under the petrography of Kaiata Formation .Significant points include : (1) the presence of abundant framboidal pyrite , formed from the reaction between natural iron salts and hydrogen sulphide produced by saprophytic bacteria ; (2) the absence of calcareous microfossils because of the dissolution of their tests in sulphuric acid produced by sulphur oxidising bacteria (e.g. Thiobacillus) ; (3) the presence of large septarian concretions that formed as a carbonate gel (shrinkage cracks) within the top few metres of sediment (thin sections of concretion centres show that porosity approaches that of the original sediment) , the carbonate for which probably derives from step 2 ; (4) the presence of small rhombs of siderite , often nucleating around a small oil-spot which may represent the residuum of the organic material that could have provided the necessary ferrous iron , while the carbonate again derives from step 2 ; (5) the presence of authigenic quartz , as replacement of calcite shell material and as a cement within the concretions that form around such shell fragments .The precise cause for the precipitation of this quartz is unknown , but its perfect crystal form and close association with pyrite and siderite suggest a biochemical origin (see also Krauskopf 1967 and Germann 1971) . Verified

Clark, Sandra H.; Mosier, Elwin L., 1989, Barite nodules in Devonian shale and mudstone of western Virginia, Bulletin, , , , 10.3133/b1880, https://pubs.er.usgs.gov/publication/b1880
Millboro, http://ngmdb.usgs.gov/Geolex/Units/Millboro_2778.html
Although barite does not occur as discrete nodules at locality 8 , as at the other localities described , this locality is included because of the similarity in geologic setting to the other

localities in the Millboro Shale and because of the occurrence of barite within pyrite nodules .Equigranular baritic nodules occur south of McDowell (locality 9 , table 1 , fig. 1) but are rare .Baritic , calcitic , and pyritic nodules are in dark-gray to black shale that 6 Barite Nodules in Devonian Shale and Mudstone of Western Virginia Figure 5 . Verified

FELDMANN, R. M.; FRANTESCU, A.; FRANTESCU, O. D.; KLOMPMAKER, A. A.; LOGAN, G.; ROBINS, C. M.; SCHWEITZER, C. E.; WAUGH, D. A., 2012, FORMATION OF LOBSTER-BEARING CONCRETIONS IN THE LATE CRETACEOUS BEARPAW SHALE, MONTANA, UNITED STATES, IN A COMPLEX GEOCHEMICAL ENVIRONMENT, PALAIOS, 27, 12, 842--856, 10.2110/palo.2012.p12-035r, http://dx.doi.org/10.2110/palo.2012.p12-035r
Bearpaw, http://weblex.nrcan.gc.ca/html/000000/GSCC00053000966.html
Eleven concretions containing the nephropid lobster , Palaeonephrops browni (Whitfield , 1907) , from the Upper Cretaceous (Campanian) , Bearpaw Formation in northeastern Montana , were examined using visual and geochemical methods .The concretions were zoned , with an axial , phosphate-rich core also containing calcium surrounding the lobster remains and an outer , calcium-rich zone lacking phosphate .The overall composition documents these as carbonate concretions , not phosphatic concretions .Where visible , the inner zone is sheathed in a thin layer dominated by framboidal pyrite , suggesting formation by a microbial film . Verified

xDD pyrite hits

This list includes all papers mentioning pyrite framboids, nodules or concretions. Please see GitHub (https://github.com/jemmings-git/pyrite_analysis) for the complete reference list, including undifferentiated pyrite mentions.

Agangi, Andrea; Hofmann, Axel; Eickmann, Benjamin; Marin-Carbonne, Johanna; Reddy, Steven M., 2016, An atmospheric source of S in Mesoarchaean structurally-controlled gold mineralisation of the Barberton Greenstone Belt, Precambrian Research, 285, 10--20, 10.1016/j.precamres.2016.09.004

Aguilera, Orangel; de Aguilera, Dione Rodrigues, 2001, An exceptional coastal upwelling fish assemblage in the Caribbean Neogene, Journal of Paleontology, 75, 03, 732--742, 10.1017/S0022336000039767

Allen, J.R.L.; Fulford, M.G.; Todd, J.A., 2007, Burnt Kimmeridgian Shale At Early Roman Silchester, South-East England, And The Roman Poole? Purbeck Complex-Agglomerated Geomaterials Industry, Oxford Journal Of Archaeology, 26, 2, 167--191, 10.1111/J.1468-0092.2007.00279.X

Allison, C.W., 1988, Paleontology of late Proterozoic and Early Cambrian rocks of east-central Alaska, Professional Paper, 10.3133/pp1449

Allison, Carol W.; Moorman, Mary A., 1974, Pyritized Microfossils and Pyrite Framboids: Reply, Geol, 2, 4, 202, 10.1130/0091-7613(1974)2<202:pmapfr>2.0.co;2

Azaraien, Hassan; Shahabpour, Jamshid; Aminzadeh, Balandeh, 2017, Metallogenesis of the sediment-hosted stratiform Cu deposits of the Ravar Copper Belt (RCB), Central Iran, Ore Geology Reviews, 81, 369--395, 10.1016/j.oregeorev.2016.09.035

B. Thusu, 1972, Depositional Environments of the Rochester Formation (Middle Silurian) in Southern Ontario, SEPM Journal of Sedimentary Research, Vol. 42, 10.1306/74d72685-2b21-11d7-8648000102c1865d

BAI, Baojun; SUN, Yongpeng; LIU, Lingbo, 2016, Petrophysical properties characterization of Ordovician Utica gas shale in Quebec, Canada, Petroleum Exploration and Development, 43, 1, 74--81, 10.1016/S1876-3804(16)30008-8

Banerjee, Indranil; Ghosh, Santosh K.; Abercrombie, Hugh J.; Davies, Edward H., 1994, An integrated subsurface study of the Mannville–Colorado group boundary in the Cessford Field, Alberta, Canadian Journal of Earth Sciences, 31, 3, 489--504, 10.1139/e94-044

Barras, Colin G.; Twitchett, Richard J., 2007, Response of the marine infauna to Triassic–Jurassic environmental change: Ichnological data from southern England, Palaeogeography, Palaeoclimatology, Palaeoecology, 244, 1-4, 223--241, 10.1016/j.palaeo.2006.06.040

Binda, P.L.; Van Eden, J.G., 1972, Sedimentological evidence on the origin of the Precambrian Great Conglomerate (Kundelungu Tillite), Zambia, Palaeogeography, Palaeoclimatology, Palaeoecology, 12, 3, 151--168, 10.1016/0031-0182(72)90057-0

Bond, David P.G.; Wignall, Paul B., 2010, Pyrite framboid study of marine Permian–Triassic boundary sections: A complex anoxic event and its relationship to contemporaneous mass extinction, Geological Society of America Bulletin, 122, 7-8, 1265--1279, 10.1130/b30042.1

Bottomley, Dennis J; Veizer, Jan; Nielsen, Heimo; Moczydlowska, Malgorzata, 1992, Isotopic composition of disseminated sulfur in Precambrian sedimentary rocks, Geochimica et Cosmochimica Acta, 56, 8, 3311--3322, 10.1016/0016-7037(92)90307-5

Burley, S.D.; Mullis, J.; Matter, A., 1989, Timing diagenesis in the Tartan Reservoir (UK North Sea): constraints from combined cathodoluminescence microscopy and fluid inclusion studies, Marine and Petroleum Geology, 6, 2, 98--120, 10.1016/0264-8172(89)90014-7

Bush, Alfred Lerner, 1987, Contributions to mineral resources research, 1984, Bulletin, 10.3133/b1694

Carmona, Noelia B.; Ponce, Juan José; Wetzel, Andreas; Bournod, Constanza N.; Cuadrado, Diana G., 2012, Microbially induced sedimentary structures in Neogene tidal flats from Argentina: Paleoenvironmental, stratigraphic and taphonomic implications, Palaeogeography, Palaeoclimatology, Palaeoecology, 1--9, 10.1016/j.palaeo.2012.06.021

Carrigan, William J.; Cameron, Eion M., 1991, Petrological and stable isotope studies of carbonate and sulfide minerals from the Gunflint Formation, Ontario: evidence for the origin of early Proterozoic iron-formation, Precambrian Research, 52, 3-4, 347--380, 10.1016/0301-9268(91)90088-R

Carter, Lorna M.H., 1988, USGS research on energy resources, 1988; program and abstracts, Circular, 10.3133/cir1025

Carter, R.M., 1976, Stratigraphy of Maruia and Matiri Formations in their type section (Trent stream, Matiri river, Murchison), Journal of the Royal Society of New Zealand, 6, 4, 459--487, 10.1080/03036758.1976.10421485

Chambers, M.H.; Lawrence, D.S.L.; Sellwood, B.W.; Parker, A., 2000, Annual layering in the Upper Jurassic Kimmeridge clay formation, UK, quantified using an ultra-high resolution SEM-EDX investigation, Sedimentary Geology, 137, 1-2, 9--23, 10.1016/S0037-0738(00)00092-0

Clark, Sandra H.; Mosier, Elwin L., 1989, Barite nodules in Devonian shale and mudstone of western Virginia, Bulletin, 10.3133/b1880

Clohessy, S.; Appleyard, S.; Vogwill, R., 2013, Groundwater acidification near the water table of the Superficial aquifer, Gnangara Mound, Swan Coastal Plain, Western Australia, Applied Geochemistry, 140--152, 10.1016/j.apgeochem.2013.06.003

Cloud, Preston, 1976, Beginnings of biospheric evolution and their biogeochemical consequences, Paleobiology, 2, 04, 351--387, 10.1017/S009483730000498X

Cohee, George Vincent; West, Walter S.; Wilkie, Lorna C., 1967, Changes in stratigraphic nomenclature by the U.S. Geological Survey, 1966, Bulletin, 10.3133/b1254A

Cotroneo, S.; Schiffbauer, J. D.; McCoy, V. E.; Wortmann, U. G.; Darroch, S. A. F.; Peng, Y.; Laflamme, M., 2016, A new model of the formation of Pennsylvanian iron carbonate concretions hosting exceptional soft-bodied fossils in Mazon Creek, Illinois, Geobiology, 10.1111/gbi.12197

Criddle, A.J., NA, A Preliminary Description of Microcrystalline Pyrite from the Nannoplankton Ooze at Site 251, Southwest Indian Ocean, Initial Reports of the Deep Sea Drilling Project, 26, 10.2973/dsdp.proc.26.126.1974

Dalrymple, R. W.; Narbonne, G. M., 1996, Continental slope sedimentation in the Sheepbed Formation (Neoproterozoic, Windermere Supergroup), Mackenzie Mountains, N.W.T., Canadian Journal of Earth Sciences, 33, 6, 848--862, 10.1139/e96-064

Daws, J. A.; Prosser, D. J., 1992, Scales Of Permeability Heterogeneity Within The Brent Group, Journal of Petroleum Geology, 15, 4, 397--417, 10.1111/j.1747-5457.1992.tb01042.x

Dineley, D. L.; Williams, B. P. J., 1968, The Devonian continental rocks of the lower Restigouche River, Quebec, Canadian Journal of Earth Sciences, 5, 4, 945--953, 10.1139/e68-091

Dogramaci, Shawan; McLean, Laura; Skrzypek, Grzegorz, 2017, Hydrochemical and stable isotope indicators of pyrite oxidation in carbonate-rich environment; the Hamersley Basin, Western Australia, Journal of Hydrology, 288--298, 10.1016/j.jhydrol.2016.12.009

Dong, Tian; Harris, Nicholas B.; Ayranci, Korhan; Twemlow, Cory E.; Nassichuk, Brent R., 2017, The impact of composition on pore throat size and permeability in high maturity shales: Middle and Upper Devonian Horn River Group, northeastern British Columbia, Canada, Marine and Petroleum Geology, 220--236, 10.1016/j.marpetgeo.2017.01.011

Dow, J. A. S.; Neall, V. E., 1974, Geology of the Lower Rennick Glacier, Northern Victoria Land, Antarctica, New Zealand Journal of Geology and Geophysics, 17, 3, 659--714, 10.1080/00288306.1973.10421588

Driedger, Carolyn L.; Kennard, Paul M., 1986, Ice Volumes on Cascade Volcanoes: Mount Rainier, Mount Hood, Three Sisters, and Mount Shasta, Professional Paper, 10.3133/pp1365

Dyck, W.; McCorkell, R. H., 1983, A study of uranium-rich reduction spheroids in sandstones from Pugwash Harbour, Nova Scotia, Canadian Journal of Earth Sciences, 20, 11, 1738--1746, 10.1139/e83-163

Erlich, R. N.; Farfan, P. F.; Hallock, P., 1993, Biostratigraphy, depositional environments, and diagenesis of the Tamana Formation, Trinidad: a tectonic marker horizon, Sedimentology, 40, 4, 743--768, 10.1111/j.1365-3091.1993.tb01358.x

Faggetter, Luke E.; Wignall, Paul B.; Pruss, Sara B.; Newton, Robert J.; Sun, Yadong; Crowley, Stephen F., 2017, Trilobite extinctions, facies changes and the ROECE carbon isotope excursion at the Cambrian Series 2–3 boundary, Great Basin, western USA, Palaeogeography, Palaeoclimatology, Palaeoecology, 478, 53--66, 10.1016/j.palaeo.2017.04.009

Fallick, Anthony E.; Boyce, Adrian J.; McConville, Paul, 2012, Sulphur stable isotope systematics in diagenetic pyrite from the North Sea hydrocarbon reservoirs revealed by laser combustion analysis, Isotopes in Environmental and Health Studies, 48, 1, 144--165, 10.1080/10256016.2012.658791

Feldmann, R. M.; Frantescu, A.; Frantescu, O. D.; Klompmaker, A. A.; Logan, G.; Robins, C. M.; Schweitzer, C. E.; Waugh, D. A., 2012, Formation Of Lobster-Bearing Concretions In The Late Cretaceous Bearpaw Shale, Montana, United States, In A Complex Geochemical Environment, Palaios, 27, 12, 842--856, 10.2110/Palo.2012.P12-035r

Feldmann, Rodney M.; Rodriguez, M. Fernanda; Martinez, Gabriela A.; Aguirre-Urreta, María, 1997, Costacopluma salamanca new species (Decapoda, Retroplumidae) from the Salamanca Formation (Danian) of Patagonia, Argentina, Journal of Paleontology, 71, 01, 125--130, 10.1017/S0022336000039019

Fisher, Donald M, 1990, Orientation history and rheology in slates, Kodiak and Afognak Islands, Alaska, Journal of Structural Geology, 12, 4, 483--498, 10.1016/0191-8141(90)90036-X

Folkestad, Atle; Veselovsky, Zbynek; Roberts, Paul, 2012, Utilising borehole image logs to interpret delta to estuarine system: A case study of the subsurface Lower Jurassic Cook Formation in the Norwegian northern North Sea, Marine and Petroleum Geology, 29, 1, 255--275, 10.1016/j.marpetgeo.2011.07.008

Franke, O. L.; McClymonds, N. E., 1972, Summary of the hydrologic situation on Long Island, New York, as a guide to water-management alternatives, Professional Paper,

Fraser, Tiffani A.; Hutchison, Matt P., 2017, Lithogeochemical characterization of the Middle-Upper Devonian Road River Group, Canol and Imperial formations on Trail River, east Richardson Mountains, Yukon: age constraints and a depositional model for fine-grained strata in the Lower Paleozoic Richardson trough, Canadian Journal of Earth Sciences, 10.1139/cjes-2016-0216

Garvin, P.L.; Ludvigson, G.A., 1993, Epigenetic sulfide mineralization associated with Pennsylvanian paleokarst in eastern Iowa, U.S.A., Chemical Geology, 105, 4, 271--290, 10.1016/0009-2541(93)90131-2

Geldsetzer, Helmut H. J.; Goodfellow, Wayne D.; McLaren, Digby J.; Orchard, Mike J., 1987, Sulfur-isotope anomaly associated with the Frasnian-Famennian extinction, Medicine Lake, Alberta, Canada, Geol, 15, 5, 393, 10.1130/0091-7613(1987)15<393:saawtf>2.0.co;2

Gill, James R.; Cobban, William Aubrey; Schultz, Leonard Gene, 1972, Stratigraphy and composition of the Sharon Springs Member of the Pierre Shale in western Kansas, Professional Paper,

Glumac, B.; Walker, K. R., 1998, A Late Cambrian positive carbon-isotope excursion in the Southern Appalachians; relation to biostratigraphy, sequence stratigraphy, environments of deposition, and diagenesis, Journal of Sedimentary Research, 68, 6, 1212--1222, 10.2110/jsr.68.1212

Glumac, B.; Walker, K. R., 2002, Effects of Grand-Cycle Cessation on the Diagenesis of Upper Cambrian Carbonate Deposits in the Southern Appalachians, U.S.A., Journal of Sedimentary Research, 72, 4, 570--586, 10.1306/111501720570

Graham, G.E.; Kelley, K.D., 2009, The Drenchwater deposit, Alaska: An example of a natural low pH environment resulting from weathering of an undisturbed shale-hosted Zn–Pb–Ag deposit, Applied Geochemistry, 24, 2, 232--245, 10.1016/j.apgeochem.2008.11.016

Grotzinger, 2017, Pyrite-walled tube structures in a Mesoproterozoic sediment-hosted metal sulfide deposit, GSA Bulletin, 10.1130/b31504.1

Han, Kui; Ju, Yiwen; Wang, Guochang; Bao, Shujing; Bu, Hongling; Neupane, Bhupati, 2016, Shale composition and pore structure variations in the progradation direction: A case study of transitional shales in the Xu-Huai district, southern North China, Journal of Natural Gas Science and Engineering, 36, 1178--1187, 10.1016/j.jngse.2016.03.022

Harrison, R. W.; Litwin, R. J.; Repetski, J. E.; Mason, David; Schultz, A. P., 1996, Results of drilling in the English Hill area, Benton Hills, Scott County, Missouri, Open-File Report,

Haruna, Makoto; Hanamuro, Takahiro; Uyeda, Kaoru; Fujimaki, Hirokazu; Ohmoto, Hiroshi, 2003, Chemical, Isotopic, and Fluid Inclusion Evidence for the Hydrothermal Alteration of the Footwall Rocks of the BIF-Hosted Iron Ore Deposits in the Hamersley District, Western Australia, Resource Geology, 53, 2, 75--88, 10.1111/j.1751-3928.2003.tb00160.x

Hassler, S. W.; Simonson, B. M.; Sumner, D. Y.; Murphy, M., 2005, Neoarchaean impact spherule layers in the Fortescue and Hamersley Groups, Western Australia: stratigraphic and depositional implications of re-correlation, Australian Journal of Earth Sciences, 52, 4-5, 759--771, 10.1080/08120090500170369

Hewitt, Roger A., 1988, Nautiloid shell taphonomy: Interpretations based on water pressure, Palaeogeography, Palaeoclimatology, Palaeoecology, 63, 1-3, 15--25, 10.1016/0031-0182(88)90088-0

Hiatt, Eric E.; Pufahl, Peir K.; Edwards, Cole T., 2015, Sedimentary phosphate and associated fossil bacteria in a Paleoproterozoic tidal flat in the 1.85Ga Michigamme Formation, Michigan, USA, Sedimentary Geology, 24--39, 10.1016/j.sedgeo.2015.01.006

Hieshima, G.B.; Pratt, L.M., 1991, Sulfur/carbon ratios and extractable organic matter of the middle proterozoic nonesuch formation, north american midcontinent rift, Precambrian Research, 54, 1, 65--79, 10.1016/0301-9268(91)90069-M

Hoffman, Paul, 1969, Proterozoic paleocurrents and depositional history of the East Arm fold belt, Great Slave Lake, Northwest Territories, Canadian Journal of Earth Sciences, 6, 3, 441--462, 10.1139/e69-042

Hudson, J. D.; Coleman, M. L.; Barreiro, B. A.; Hollingworth, N. T. J., 2001, Septarian concretions from the Oxford Clay (Jurassic, England, UK): involvement of original marine and multiple external pore fluids, Sedimentology, 48, 3, 507--531, 10.1046/j.1365-3091.2001.00374.x

Hurd, Gregory S.; Kerans, Charles; Fullmer, Shawn; Janson, Xavier, 2016, Large-Scale Inflections in Slope Angle Below the Shelf Break: A First Order Control On the Stratigraphic Architecture of Carbonate Slopes: Cutoff Formation, Guadalupe Mountains National Park, West Texas, U.S.A., Journal of Sedimentary Research, 86, 4, 336--362, 10.2110/jsr.2016.25

Imlay, Ralph Willard, 1976, Middle Jurassic (Bajocian and Bathonian) ammonites from northern Alaska, Professional Paper,

Jell, John S.; Cook, Alex G.; Jell, Peter A., 2011, Australian Cretaceous Cnidaria and Porifera, Alcheringa: An Australasian Journal of Palaeontology, 35, 2, 241--284, 10.1080/03115518.2011.532322

Johnson, C. L.; Graham, S. A., 2004, Cycles in Perilacustrine Facies of Late Mesozoic Rift Basins, Southeastern Mongolia, Journal of Sedimentary Research, 74, 6, 786--804, 10.1306/051304740786

Kelly, S.R.A.; Rawson, P.F., 1983, Some late Jurassic – mid-Cretaceous sections on the East Midlands Shelf, England, as demonstrated on a Field Meeting, 18-20 May 1979, Proceedings of the Geologists' Association, 94, 1, 65--73, 10.1016/S0016-7878(83)80028-5

Kiel, Steffen, 2008, Fossil evidence for micro- and macrofaunal utilization of large nekton-falls: Examples from early Cenozoic deep-water sediments in Washington State, USA, Palaeogeography, Palaeoclimatology, Palaeoecology, 267, 3-4, 161--174, 10.1016/j.palaeo.2008.06.016

King, Philip Burke; Henbest, Lloyd G.; Yochelson, E.L.; Cloud, P.E.; Duncan, Helen; Finks, R.M.; Sohn, I.G., 1965, Geology of the Sierra Diablo region, Texas, with special determinative studies of Permian fossils, Professional Paper, 10.3133/pp480

Klaver, Jop; Desbois, Guillaume; Littke, Ralf; Urai, Janos L., 2015, BIB-SEM characterization of pore space morphology and distribution in postmature to overmature samples from the Haynesville and Bossier Shales, Marine and Petroleum Geology, 451--466, 10.1016/j.marpetgeo.2014.09.020

Komatsu, Toshifumi; Takashima, Reishi; Shigeta, Yasunari; Maekawa, Takumi; Tran, Huyen Dang; Cong, Tien Dinh; Sakata, Susumu; Dinh, Hung Doan; Takahashi, Osamu, 2016, Carbon isotopic excursions and detailed ammonoid and conodont biostratigraphies around Smithian–Spathian boundary in the Bac Thuy Formation, Vietnam, Palaeogeography, Palaeoclimatology, Palaeoecology, 65--74, 10.1016/j.palaeo.2016.04.017

Kster, J.; Littke, R.; Machel, H. G., 2008, Devonian Carbonates Of The Nigel Peak Area, Rocky Mountains, Canada: A Fossil Petroleum System, Journal Of Petroleum Geology, 31, 3, 283--301, 10.1111/J.1747-5457.2008.00421.X

Law, B.E.; Spencer, Charles Winthrop, 1989, Geology of tight gas reservoirs in the Pinedale Anticline area, Wyoming, and at the Multiwell Experiment site, Colorado, Bulletin, 10.3133/b1886

le Roux, J.P., 1993, Genesis of stratiform U-Mo deposits in the Karoo Basin of South Africa, Ore Geology Reviews, 7, 6, 485--509, 10.1016/0169-1368(93)90012-N

Leckie, Dale A.; Kjarsgaard, B. A.; Bloch, John; McIntyre, David; McNeil, David; Stasiuk, Laverne; Heaman, Larry, 1997, Emplacement and reworking of Cretaceous, diamond-bearing, crater facies kimberlite of central Saskatchewan, Canada, Geological Society of America Bulletin, 109, 8, 1000--1020, 10.1130/0016-7606(1997)109<1000:earocd>2.3.co;2

Lecomte, Andreï; Cathelineau, Michel; Michels, Raymond; Peiffert, Chantal; Brouand, Marc, 2017, Uranium mineralization in the Alum Shale Formation (Sweden): Evolution of a U-rich marine black shale from sedimentation to metamorphism, Ore Geology Reviews, 88, 71--98, 10.1016/j.oregeorev.2017.04.021

LoDuca, Steven T.; Wu, Mengyin; Zhao, Yuanlong; Xiao, Shuhai; Schiffbauer, James D.; Caron, Jean-Bernard; Babcock, Loren E., 2015, Reexamination of Yuknessia from the Cambrian of China and first report of Fuxianospira from North America, Journal of Paleontology, 89, 06, 899--911, 10.1017/jpa.2016.3

Löhr, S.C.; Baruch, E.T.; Hall, P.A.; Kennedy, M.J., 2015, Is organic pore development in gas shales influenced by the primary porosity and structure of thermally immature organic matter?, Organic Geochemistry, 119--132, 10.1016/j.orggeochem.2015.07.010

López-Martínez, Rafael; Barragán, Ricardo; Bernal, Juan Pablo; Reháková, Daniela; Gómez-Tuena, Arturo; Martini, Michelangelo; Ortega, Carlos, 2017, Integrated stratigraphy and isotopic ages at the Berriasian–Valanginian boundary at Tlatlauquitepec (Puebla, Mexico), Journal of South American Earth Sciences, 1--10, 10.1016/j.jsames.2016.12.003

Loucks, R. G.; Reed, R. M.; Ruppel, S. C.; Jarvie, D. M., 2009, Morphology, Genesis, and Distribution of Nanometer-Scale Pores in Siliceous Mudstones of the Mississippian Barnett Shale, Journal of Sedimentary Research, 79, 12, 848--861, 10.2110/jsr.2009.092

Lowery, Christopher M.; Corbett, Matthew J.; Leckie, R. Mark; Watkins, David; Miceli Romero, Andrea; Pramudito, Aris, 2014, Foraminiferal and nannofossil paleoecology and paleoceanography of the Cenomanian–Turonian Eagle Ford Shale of southern Texas, Palaeogeography, Palaeoclimatology, Palaeoecology, 49--65, 10.1016/j.palaeo.2014.07.025

Luczaj, J. A.; Goldstein, R. H., 2000, Diagenesis of the Lower Permian Krider Member, Southwest Kansas, U.S.A.: Fluid-Inclusion, U-Pb, and Fission-Track Evidence for Reflux Dolomitization During Latest Permian Time, Journal of Sedimentary Research, 70, 3, 762--773, 10.1306/2dc40936-0e47-11d7-8643000102c1865d

Lyons, Timothy W; Luepke, James J; Schreiber, Madeline E; Zieg, Gerald A, 2000, Sulfur geochemical constraints on mesoproterozoic restricted marine deposition: lower Belt Supergroup, northwestern United States, Geochimica et Cosmochimica Acta, 64, 3, 427--437, 10.1016/S0016-7037(99)00323-3

Marintsch, Edward J.; Finks, Robert M., 1978, Zoophycos Size May Indicate Environmental Gradients, Lethaia, 11, 4, 273--279, 10.1111/J.1502-3931.1978.Tb01880.X

Martinez R, J.I.; Hernandez, R., 1992, Evolution and drowning of the late cretaceous Venezuelan carbonate platform, Journal of South American Earth Sciences, 5, 2, 197--210, 10.1016/0895-9811(92)90038-Z

Mathieu, J.; Kontak, D.J.; Turner, E.C.; Fayek, M.; Layne, G., 2015, Geochemistry of Phanerozoic Diagenesis on Victoria Island, NWT, Canada, Chemical Geology, 10.1016/j.chemgeo.2015.08.016

Mcbride, Earle F.; Milliken, Kitty L., 2006, Giant calcite-cemented concretions, Dakota Formation, central Kansas, USA, Sedimentology, 53, 5, 1161--1179, 10.1111/j.1365-3091.2006.00813.x

McCabe, Chad; Van der Voo, Rob; Peacor, Donald R.; Scotese, Christopher R.; Freeman, Roy, 1983, Diagenetic magnetite carries ancient yet secondary remanence in some Paleozoic sedimentary carbonates, Geol, 11, 4, 221, 10.1130/0091-7613(1983)11<221:dmcays>2.0.co;2

McIlroy, Duncan, 2000, A lower Cambrian protoconodont apparatus from the Placentian of southeastern Newfoundland, Lethaia, 33, 2, 95--102, 10.1080/00241160050150230

Mehlqvist, Kristina; Einarsson, Elisabeth, 2011, Lundadagarna i Historisk Geologi och Paleonotologi XII 17–18th of March, 2011, GFF, 133, 1-2, 57--75, 10.1080/11035897.2011.565885

Michel, F.A., 1986, Hydrogeology of the central Mackenzie Valley, Journal of Hydrology, 85, 3-4, 379--405, 10.1016/0022-1694(86)90068-5

Mills, Joseph W., 1974, Galena-Bearing Pyrite Nodules in the Nelway Formation, Salmo, British Columbia, Canadian Journal of Earth Sciences, 11, 4, 495--502, 10.1139/e74-043

Millward, David; Davies, Sarah J.; Williamson, Fiona; Curtis, Rachel; Kearsey, Timothy I.; Bennett, Carys E.; Marshall, John E. A.; Browne, Michael A. E., NA, Early Mississippian evaporites of coastal tropical wetlands, Sedimentology, 10.1111/sed.12465

Morin, R.H.; Sorey, M.L.; Jacobson, R.D., 1993, Results of the flowmeter-injection test in the Long Valley Exploratory Well (Phase II), Long Valley, California, Water-Resources Investigations Report, 10.3133/wri934127

Mortimore, R.N.; Pomerol, B., 1987, Correlation of the Upper Cretaceous White Chalk (Turonian to Campanian) in the Anglo-Paris Basin, Proceedings of the Geologists' Association, 98, 2, 97--143, 10.1016/S0016-7878(87)80001-9

Murton, Julian B.; Bowen, David Q.; Candy, Ian; Catt, John A.; Currant, Andrew; Evans, John G.; Frogley, Michael R.; Green, Christopher P.; Keen, David H.; Kerney, Michael P.; Parish, David; Penkman, Kirsty; Schreve, Danielle C.; Taylor, Sheila; Toms, Phillip S.; Worsley, Peter; York, Linda L., 2015, Middle and Late Pleistocene environmental history of the Marsworth area, south-central England, Proceedings of the Geologists' Association, 10.1016/j.pgeola.2014.11.003

Neef, G., 1981, Cenozoic stratigraphy and structure of Karamea-Little Wanganui district, Buller, South Island, New Zealand, New Zealand Journal of Geology and Geophysics, 24, 2, 177--208, 10.1080/00288306.1981.10422713

Nelson, Campbell S., 1978, Stratigraphy and paleontology of the oligocene Te Kuiti group, Waitomo County, South Auckland, New Zealand, New Zealand Journal of Geology and Geophysics, 21, 5, 553--594, 10.1080/00288306.1978.10424086

Nelson, Gabriel J.; Pufahl, Peir K.; Hiatt, Eric E., 2010, Paleoceanographic constraints on Precambrian phosphorite accumulation, Baraga Group, Michigan, USA, Sedimentary Geology, 226, 1-4, 9--21, 10.1016/j.sedgeo.2010.02.001

Norris, Michelle S.; Hallam, Anthony, 1995, Facies variations across the Middle-Upper Jurassic boundary in Western Europe and the relationship to sea-level changes, Palaeogeography, Palaeoclimatology, Palaeoecology, 116, 3-4, 189--245, 10.1016/0031-0182(94)00096-Q

Núñez-Useche, Fernando; Canet, Carles; Barragán, Ricardo; Alfonso, Pura, 2016, Bioevents and redox conditions around the Cenomanian–Turonian anoxic event in Central Mexico, Palaeogeography, Palaeoclimatology, Palaeoecology, 205--226, 10.1016/j.palaeo.2016.01.035

O’brien, Lorna J.; Braddy, Simon J.; Radley, Jonathan D., 2009, A new arthropod resting trace and associated suite of trace fossils from the Lower Jurassic of Warwickshire, England, Palaeontology, 52, 5, 1099--1112, 10.1111/j.1475-4983.2009.00901.x

Olempska, Ewa; Wacey, David, 2016, Ambient inclusion trails in Palaeozoic crustaceans (Phosphatocopina and Ostracoda), Palaeogeography, Palaeoclimatology, Palaeoecology, 441, 949--958, 10.1016/j.palaeo.2015.10.052

Park, John K., 1995, Paleomagnetism of the late Neoproterozoic Blueflower and Risky formations of the northern Cordillera, Canada, Canadian Journal of Earth Sciences, 32, 6, 718--729, 10.1139/e95-061

Pates, Stephen; Daley, Allison C.; Lieberman, Bruce S., 2018, Hurdiid radiodontans from the middle Cambrian (Series 3) of Utah, Journal of Paleontology, 92, 01, 99--113, 10.1017/jpa.2017.11

Phiri, Cryton; Wang, Pujun; Nyambe, Imasiku Anayawa, 2016, Geology and potential hydrocarbon play system of Lower Karoo Group in the Maamba Coalfield Basin, southern Zambia, Journal of African Earth Sciences, 245--262, 10.1016/j.jafrearsci.2016.03.006

Pirajno, Franco; Burlow, Rick; Huston, David, 2010, The Magellan Pb deposit, Western Australia; a new category within the class of supergene non-sulphide mineral systems, Ore Geology Reviews, 37, 2, 101--113, 10.1016/j.oregeorev.2010.01.001

Pojeta, John, 1986, Devonian rocks and Lower and Middle Devonian pelecypods of Guangxi, China, and the Traverse Group of Michigan, Professional Paper, 10.3133/pp1394AG

Pratt, Lisa M.; Summons, Roger E.; Hieshima, Glenn B., 1991, Sterane and triterpane biomarkers in the Precambrian Nonesuch Formation, North American Midcontinent Rift, Geochimica et Cosmochimica Acta, 55, 3, 911--916, 10.1016/0016-7037(91)90351-5

Prudic, David E., 1986, Ground-water hydrology and subsurface migration of radionuclides at a commercial radioactive-waste burial site, West Valley, Cattaraugus County, New York, Professional Paper, 10.3133/pp1325

Rahman, Habibur M.; Kennedy, Martin; Löhr, Stefan; Dewhurst, David N.; Sherwood, Neil; Yang, Shengyu; Horsfield, Brian, 2018, The influence of shale depositional fabric on the kinetics of hydrocarbon generation through control of mineral surface contact area on clay catalysis, Geochimica et Cosmochimica Acta, 220, 429--448, 10.1016/j.gca.2017.10.012

Rahman, Habibur M.; Kennedy, Martin; Löhr, Stefan; Dewhurst, David N., 2017, Clay-organic association as a control on hydrocarbon generation in shale, Organic Geochemistry, 42--55, 10.1016/j.orggeochem.2017.01.011

Ramón, J.C.; Dzou, L.I.; Hughes, W.B.; Holba, A.G., 2001, Evolution of the Cretaceous organic facies in Colombia: implications for oil composition, Journal of South American Earth Sciences, 14, 1, 31--50, 10.1016/S0895-9811(01)00010-4

Retallack, G. J., 2014, Affirming life aquatic for the Ediacara biota in China and Australia: COMMENT, Geology, 42, 3, e325--e325, 10.1130/g35030c.1

Retallack, Gregory J., 2014, Volcanosedimentary paleoenvironments of Ediacaran fossils in Newfoundland, Geological Society of America Bulletin, 126, 5-6, 619--638, 10.1130/b30892.1

Reynolds, Richard L.; Fishman, Neil S.; Wanty, Richard B.; Goldhaber, Martin B., 1990, Iron sulfide minerals at Cement oil field, Oklahoma: Implications for magnetic detection of oil fields, Geological Society of America Bulletin, 102, 3, 368--380, 10.1130/0016-7606(1990)102<0368:ismaco>2.3.co;2

Richard M. Pollastro, 1981, Authigenic Kaolinite and Associated Pyrite in Chalk of the Cretaceous Niobrara Formation, Eastern Colorado, SEPM Journal of Sedimentary Research, Vol. 51, 10.1306/212f7cd4-2b24-11d7-8648000102c1865d

Rijsdijk, K. F.; Kroon, I. C.; Meijer, T.; Passchier, S.; Van Dijk, T. A. G. P.; Bunnik, F. P. M.; Janse, A. C., 2013, Reconstructing Quaternary Rhine-Meuse dynamics in the southern North Sea: architecture, seismo-lithofacies associations and malacological biozonation, Journal of Quaternary Science, 28, 5, 453--466, 10.1002/jqs.2627

Roberts, Eric M., 2007, Facies architecture and depositional environments of the Upper Cretaceous Kaiparowits Formation, southern Utah, Sedimentary Geology, 197, 3-4, 207--233, 10.1016/j.sedgeo.2006.10.001

Rogers, Karyne M.; Morgans, Hugh E.G.; Wilson, Gary S., 2001, Identification of a Waipawa Formation equivalent in the upper Te Uri Member of the Whangai Formation - implications for depositional history and age, New Zealand Journal of Geology and Geophysics, 44, 2, 347--354, 10.1080/00288306.2001.9514943

Rouchon, Véronique; Béthoux, Olivier; Ren, Dong, 2018, Gypsum growth induced by pyrite oxidation jeopardises the conservation of fossil specimens: an example from the Xiaheyan entomofauna (Late Carboniferous, China), Palaeogeography, Palaeoclimatology, Palaeoecology, 507, 15--29, 10.1016/j.palaeo.2018.05.035

Rowe, Harry D.; Loucks, Robert G.; Ruppel, Stephen C.; Rimmer, Susan M., 2008, Mississippian Barnett Formation, Fort Worth Basin, Texas: Bulk geochemical inferences and Mo–TOC constraints on the severity of hydrographic restriction, Chemical Geology, 257, 1-2, 16--25, 10.1016/j.chemgeo.2008.08.006

Ruffell, Alastair H.; Batten, David J., 1990, The Barremian-Aptian arid phase in western Europe, Palaeogeography, Palaeoclimatology, Palaeoecology, 80, 3-4, 197--212, 10.1016/0031-0182(90)90132-Q

Sageman, Bradley B; Murphy, Adam E; Werne, Josef P; Ver Straeten, Charles A; Hollander, David J; Lyons, Timothy W, 2003, A tale of shales: the relative roles of production, decomposition, and dilution in the accumulation of organic-rich strata, Middle–Upper Devonian, Appalachian basin, Chemical Geology, 195, 1-4, 229--273, 10.1016/S0009-2541(02)00397-2

Sample, J. C.; Fisher, D. M., 1986, Duplex accretion and underplating in an ancient accretionary complex, Kodiak Islands, Alaska, Geol, 14, 2, 160, 10.1130/0091-7613(1986)14<160:daauia>2.0.co;2

Sandstrom, Mark W., 1980, Organic geochemistry of some Cambrian phosphorites, Physics and Chemistry of the Earth, 123--131, 10.1016/0079-1946(79)90094-6

Sanei, Hamed; Haeri-Ardakani, Omid; Curtis, Mark E.; Akai, Takashi; Currie, Carolyn, 2018, Solid bitumen in the Montney Formation: Diagnostic petrographic characteristics and significance for hydrocarbon migration, International Journal of Coal Geology, 198, 48--62, 10.1016/j.coal.2018.09.004

Santos, C.; Jaramillo, C.; Bayona, G.; Rueda, M.; Torres, V., 2008, Late Eocene marine incursion in north-western South America, Palaeogeography, Palaeoclimatology, Palaeoecology, 264, 1-2, 140--146, 10.1016/j.palaeo.2008.04.010

Saul, Louella R.; Squires, Richard L.; Goedert, James L., 1996, A new genus of cryptic lucinid? bivalve from Eocene cold seeps and turbidite-influenced mudstone, western Washington, Journal of Paleontology, 70, 05, 788--794, 10.1017/S0022336000023829

Schieber, 2017, Association Between Wave- and Current-aided Hyperpycnites and Flooding Surfaces in Shelfal Mudstones: an Integrated Sedimentologic, Sequence Stratigraphic, and Geochemical Approach, Journal of Sedimentary Research, 87, 11, 1143--1155, 10.2110/jsr.2017.62

Schieber, J.; Baird, G., 2001, On the Origin and Significance of Pyrite Spheres in Devonian Black Shales of North America, Journal of Sedimentary Research, 71, 1, 155--166, 10.1306/051600710155

Schultz, L.G., 1965, Mineralogy and stratigraphy of the lower part of the Pierre Shale, South Dakota and Nebraska, Professional Paper, 10.3133/pp392B

Sergeev, Nikita; Burlow, Rick; Tessalina, Svetlana, 2017, The Paroo Station Mine supergene lead deposits, Western Australia: Geological and geochemical constraints, Ore Geology Reviews, 564--593, 10.1016/j.oregeorev.2016.07.017

Sharma, Sajal; Dix, George R; Riva, J FV, 2003, Late Ordovician platform foundering, its paleoceanography and burial, as preserved in separate (eastern Michigan Basin, Ottawa Embayment) basins, southern Ontario, Canadian Journal of Earth Sciences, 40, 2, 135--148, 10.1139/e02-099

Shchepetkina, A.; Gingras, M.K.; Pemberton, S.G., 2018, Reconstruction of brackish-water systems using an ichnological framework, Russian Geology and Geophysics, 59, 1, 55--71, 10.1016/j.rgg.2018.01.004

Shipboard Scientific Party, NA, Site 1168, Proceedings of the Ocean Drilling Program, 189, 10.2973/odp.proc.ir.189.103.2001

Slotznick, Sarah P.; Webb, Samuel M.; Kirschvink, Joseph L.; Fischer, Woodward W., NA, Mid-Proterozoic ferruginous conditions reflect post-depositional processes, Geophysical Research Letters, 10.1029/2018GL081496

Smale, David; Mauk, Jeffrey L.; Palmer, Julie; Soong, Raymond; Blattner, Peter, 1999, Variations in sandstone diagenesis with depth, time, and space, onshore Taranaki wells, New Zealand, New Zealand Journal of Geology and Geophysics, 42, 2, 137--154, 10.1080/00288306.1999.9514836

Snavely, P. D.; Niem, A.R.; MacLeod, N.S.; Pearl, J.E.; Rau, W.W., 1980, Makah Formation; a deep-marginal-basin sequence of late Eocene and Oligocene age in the northwestern Olympic Peninsula, Washington, Professional Paper, 10.3133/pp1162B

Spinazola, Joseph M., 1993, Simulation of changes in water levels and ground-water flow in response to water-use alternatives in the Mud Lake area, eastern Snake River plain, eastern Idaho, Water-Resources Investigations Report, 10.3133/wri934228

Steadman, Jeffrey A.; Large, Ross R.; Davidson, Garry J.; Bull, Stuart W.; Thompson, Jay; Ireland, Trevor R.; Holden, Peter, 2014, Paragenesis and composition of ore minerals in the Randalls BIF-hosted gold deposits, Yilgarn Craton, Western Australia: Implications for the

timing of deposit formation and constraints on gold sources, Precambrian Research, 110--132, 10.1016/j.precamres.2014.01.002

Strang, Katie M.; Armstrong, Howard A.; Harper, David A. T.; Trabucho-Alexandre, João P., 2016, The Sirius Passet Lagerstätte: silica death masking opens the window on the earliest matground community of the Cambrian explosion, Lethaia, 49, 4, 631--643, 10.1111/let.12174

Swanner, Elizabeth D.; Planavsky, Noah J.; Lalonde, Stefan V.; Robbins, Leslie J.; Bekker, Andrey; Rouxel, Olivier J.; Saito, Mak A.; Kappler, Andreas; Mojzsis, Stephen J.; Konhauser, Kurt O., 2014, Cobalt and marine redox evolution, Earth and Planetary Science Letters, 253--263, 10.1016/j.epsl.2014.01.001

Sweetman, Steven C.; Underwood, Charlie J., 2006, A Neoselachian Shark From The Non-Marine Wessex Formation (Wealden Group: Early Cretaceous, Barremian) Of The Isle Of Wight, Southern England, Palaeontology, 49, 2, 457--465, 10.1111/J.1475-4983.2006.00549.X

Świerczewska-Gładysz, 2018, A Record Of Sequestration Of Plant Material By Marine Burrowing Animals As A New Feeding Strategy Under Oligotrophic Conditions Evidenced By Pyrite Microtextures, Palaios, 33, 7, 312--322, 10.2110/Palo.2018.002

Taotao, Cao; Zhiguang, Song; Sibo, Wang; Xinxing, Cao; Yan, Li; Jia, Xia, 2015, Characterizing the pore structure in the Silurian and Permian shales of the Sichuan Basin, China, Marine and Petroleum Geology, 10.1016/j.marpetgeo.2014.12.007

Taylor, Graham; Walker, P. H., 1986, Tertiary Lake Bunyan, Northern Monaro, NSW, part II: Facies analysis and palaeoenvironmental implications, Australian Journal of Earth Sciences, 33, 2, 231--251, 10.1080/08120098608729362

Taylor, K.G.; Macquaker, J.H.S., 2000, Early diagenetic pyrite morphology in a mudstone-dominated succession: the Lower Jurassic Cleveland Ironstone Formation, eastern England, Sedimentary Geology, 131, 1-2, 77--86, 10.1016/S0037-0738(00)00002-6

Terfelt, Fredrik; Eriksson, Mats E.; Schmitz, Birger, 2014, The Cambrian–Ordovician transition in dysoxic facies in Baltica — diverse faunas and carbon isotope anomalies, Palaeogeography, Palaeoclimatology, Palaeoecology, 59--73, 10.1016/j.palaeo.2013.11.021

Tosdal, Richard M., 1998, Contributions to the gold metallogeny of northern Nevada, Open-File Report, 10.3133/ofr98338B

Trewin, Nigel H; Fayers, Stephen R; Kelman, Ruth, 2003, Subaqueous silicification of the contents of small ponds in an Early Devonian hot-spring complex, Rhynie, Scotland, Canadian Journal of Earth Sciences, 40, 11, 1697--1712, 10.1139/e03-065

Tribovillard, Nicolas; Hatem, Ebraheem; Averbuch, Olivier; Barbecot, Florent; Bout-Roumazeilles, Viviane; Trentesaux, Alain, 2015, Iron availability as a dominant control on the primary composition and diagenetic overprint of organic-matter-rich rocks, Chemical Geology, 401, 67--82, 10.1016/j.chemgeo.2015.02.026

Tuttle, Michele L.W.; Breit, George N.; Cozzarelli, Isabelle M., 2009, Processes affecting δ34S and δ18O values of dissolved sulfate in alluvium along the Canadian River, central Oklahoma, USA, Chemical Geology, 265, 3-4, 455--467, 10.1016/j.chemgeo.2009.05.009

van de Schootbrugge, Bas; Bachan, Aviv; Suan, Guillaume; Richoz, Sylvain; Payne, Jonathan L.; Jagt, John, 2013, Microbes, mud and methane: cause and consequence of recurrent Early Jurassic anoxia following the end-Triassic mass extinction, Palaeontology, 56, 4, 685--709, 10.1111/pala.12034

Van de Wetering, Nikola; Sanei, Hamed; Mayer, Bernhard, 2016, Organic matter characterization in mixed hydrocarbon producing areas within the Duvernay Formation, Western Canada Sedimentary Basin, Alberta, International Journal of Coal Geology, 1--11, 10.1016/j.coal.2016.01.012

Van Houten, Franklyn B., 1964, Tertiary geology of the Beaver Rim area, Fremont and Natrona Counties, Wyoming, Bulletin, 10.3133/b1164

Wacey, D.; Kilburn, M. R.; Saunders, M.; Cliff, J. B.; Kong, C.; Liu, A. G.; Matthews, J. J.; Brasier, M. D., 2014, Uncovering framboidal pyrite biogenicity using nano-scale CNorg mapping, Geology, 43, 1, 27--30, 10.1130/g36048.1

Wacey, David; Saunders, Martin; Cliff, John; Kilburn, Matt R.; Kong, Charlie; Barley, Mark E.; Brasier, Martin D., 2014, Geochemistry and nano-structure of a putative ~3240 million-year-old black smoker biota, Sulphur Springs Group, Western Australia, Precambrian Research, 1--12, 10.1016/j.precamres.2014.04.016

Warwick, Peter D.; Shakoor, Tariq, 1988, Preliminary report on coal characteristics in the Salt Range area of north-central Pakistan, Open-File Report, 10.3133/ofr88637

Weber, Bodo; Mota, Asdrulymar; Helenes, Javier; Ramírez, Rafael; Valencia, Yoryi, 2016, Age and provenance of Late Miocene-Early Pliocene sedimentary rocks from the Patao high hydrocarbon reservoir offshore NE Venezuela – U-Pb detrital zircon age, Sm-Nd isotope, and biostratigraphic data, Journal of Natural Gas Science and Engineering, 459--473, 10.1016/j.jngse.2016.03.058

Weir, Gordon Whitney; Gualtieri, James Louis; Schlanger, Seymour O., 1966, Borden Formation (Mississippian) in south and southeast-central Kentucky, Bulletin, 10.3133/b1224F

Wignall, P. B.; Newton, R., 2003, Contrasting Deep-water Records from the Upper Permian and Lower Triassic of South Tibet and British Columbia: Evidence for a Diachronous Mass Extinction, PALAIOS, 18, 2, 153--167, 10.1669/0883-1351(2003)18<153:cdrftu>2.0.co;2

Wignall, P.B; Newton, R, 2001, Black shales on the basin margin: a model based on examples from the Upper Jurassic of the Boulonnais, northern France, Sedimentary Geology, 144, 3-4, 335--356, 10.1016/S0037-0738(01)00125-7

Williford, Kenneth H.; Van Kranendonk, Martin J.; Ushikubo, Takayuki; Kozdon, Reinhard; Valley, John W., 2011, Constraining atmospheric oxygen and seawater sulfate concentrations

during Paleoproterozoic glaciation: In situ sulfur three-isotope microanalysis of pyrite from the Turee Creek Group, Western Australia, Geochimica et Cosmochimica Acta, 75, 19, 5686--5705, 10.1016/j.gca.2011.07.010

Winter, Bryce L.; Knauth, L.Paul, 1992, Stable isotope geochemistry of early Protezozoic carbonate concretions in the Animikie group of the Lake Superior region: evidence for anaerobic bacterial processes, Precambrian Research, 54, 2-4, 131--151, 10.1016/0301-9268(92)90067-X

Winter, Bryce L.; Knauth, L.Paul, 1992, Stable isotope geochemistry of cherts and carbonates from the 2.0 Ga gunflint iron formation: implications for the depositional setting, and the effects of diagenesis and metamorphism, Precambrian Research, 59, 3-4, 283--313, 10.1016/0301-9268(92)90061-R

Wisniowiecki, Michael J.; Van der Voo, Rob; McCabe, Chad; Kelly, William C., 1983, A Pennsylvanian paleomagnetic pole from the mineralized Late Cambrian Bonneterre Formation, southeast Missouri, Journal of Geophysical Research, 88, B8, 6540, 10.1029/JB088iB08p06540

Won C. Park; Erik H. Schot, 1968, Stylolites: Their Nature and Origin, SEPM Journal of Sedimentary Research, Vol. 38, 10.1306/74d71910-2b21-11d7-8648000102c1865d

Yao, Jianxin; Tong, Jinnan; Qiao, Yu; Chen, Yao, 2019, Organic matter accumulation on the Dalong Formation (Upper Permian) in western Hubei, South China: Constraints from multiple geochemical proxies and pyrite morphology, Palaeogeography, Palaeoclimatology, Palaeoecology, 514, 677--689, 10.1016/j.palaeo.2018.11.015

Zachara, John M.; Long, Philip E.; Bargar, John; Davis, James A.; Fox, Patricia; Fredrickson, Jim K.; Freshley, Mark D.; Konopka, Allan E.; Liu, Chongxuan; McKinley, James P.; Rockhold, Mark L.; Williams, Kenneth H.; Yabusaki, Steve B., 2013, Persistence of uranium groundwater plumes: Contrasting mechanisms at two DOE sites in the groundwater–river interaction zone, Journal of Contaminant Hydrology, 45--72, 10.1016/j.jconhyd.2013.02.001

xDD snippets API call

Wilkin-pyrite rocks were accessed and assessed using the following API:

https://xdd.wisc.edu/api/snippets?term=Wilkin,framboid&full_results=true&inclusive=true&clean&known_terms=stratigraphic_names

SGP API call

SGP data were called from the API using the following HTTP Post packet:

'{"type":"samples","filters":{"lithology_class":["sedimentary","metamorphic"]},"show":["fe","mn","fe_carb","fe_ox","fe_mag","fe_py","fe_py_fe_hr","fe_hr_fe_t","toc","mo","u","p","ni","v","interpreted_age","fe_hr","analysis_ref_long", "coord_lat","coord_long"]}'

Machine learning accuracy estimates

Number of resamples for cross-validation: 10

Table S1. Accuracy (Fig. S10)

	Min.	1st	Median	Mean	3rd	Max.
**lda**	0.78	0.79	0.80	0.80	0.82	0.83
**cart**	0.49	0.54	0.59	0.58	0.62	0.67
**knn**	0.89	0.90	0.91	0.91	0.91	0.92
**svm**	0.85	0.88	0.88	0.88	0.89	0.90
**rf**	0.87	0.87	0.88	0.88	0.90	0.90

Table S2. Kappa (Fig. S10)

	Min.	1st	Median	Mean	3rd	Max.
**lda**	0.72	0.73	0.75	0.75	0.76	0.79
**cart**	0.28	0.39	0.46	0.45	0.52	0.57
**knn**	0.86	0.87	0.88	0.88	0.89	0.89
**svm**	0.81	0.84	0.85	0.84	0.86	0.87
**rf**	0.83	0.84	0.85	0.85	0.87	0.87

Table S3. Confusion Matrix (kNN at k = 5)

**Prediction**	**Type 5**	**Type 2**	**Type 1**	**Type 3**	**Type 4**
**Type 5**	122	2	0	3	11
**Type 2**	1	132	6	0	3
**Type 1**	0	12	205	15	1
**Type 3**	1	2	12	239	6
**Type 4**	3	2	4	1	87

Overall Statistics (kNN at k = 5)

Accuracy : 0.9023
95% CI : (0.8806, 0.9212)
No Information Rate : 0.2966
P-Value [Acc > NIR] : < 2.2e-16
Kappa : 0.8742

Table S4. Statistics (kNN at k = 5) by class:

Class:	Type 5	Type 2	Type 1	Type 3	Type 4
Sensitivity	0.96	0.88	0.90	0.93	0.81
Specificity	0.98	0.99	0.96	0.97	0.99
Pos pred value	0.88	0.93	0.88	0.92	0.90
Neg pred value	0.99	0.98	0.97	0.97	0.97
Prevalence	0.15	0.17	0.26	0.30	0.12
Detection rate	0.14	0.15	0.24	0.27	0.10
Detection prevalence	0.16	0.16	0.27	0.30	0.11
Balanced accuracy	0.97	0.93	0.93	0.95	0.90

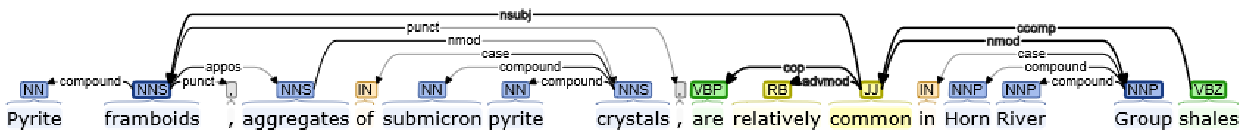

Fig. S1.

An annotated example of a pyrite-stratigraphic name tuple extraction using natural language processing (NLP). Created using Stanford CoreNLP 3.9.2 (at corenlp.run/). Example sentence from Dong et al. 2017 *Marine & Petroleum Geology*. CoreNLP identifies the grammatical relationship ('tuple') between 'pyrite framboids' and the compound noun 'Horn River Group', via the adjective 'common'.

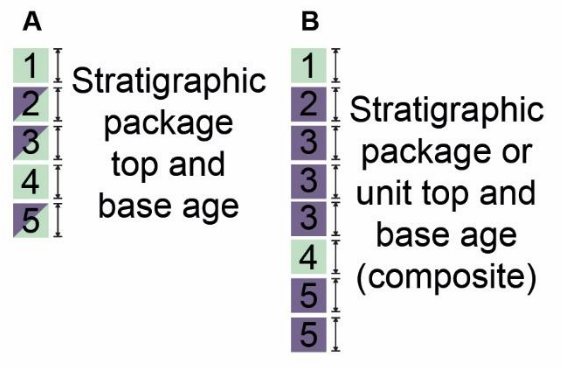

Fig. S2.

Illustration of approach to stratigraphic packages and units. (A) A record based solely on stratigraphic package top and base ages. (B) A composite record of stratigraphic packages and units, via propagation of a hypothetical number of units where defined.

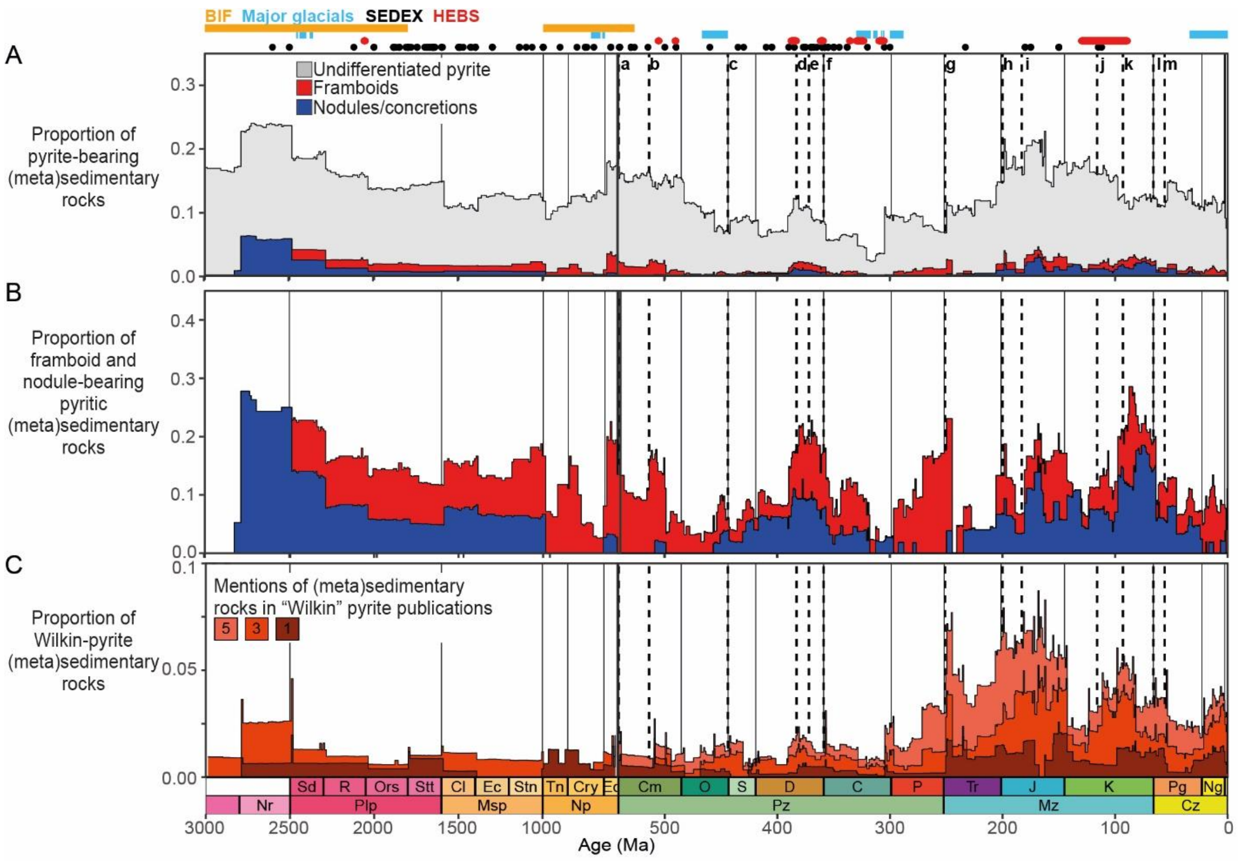

Fig. S3.

Stacked abundances of pyrite-bearing sedimentary and metasedimentary rocks through geological time, expressed as proportions of all (meta)sedimentary rocks (without propagation of Macrostrat units). Omission of Macrostrat units yields a record that is less biased towards the Macrostrat focal area at the expense of temporal resolution, and represents a spatially unweighted record. See Fig. 1 caption for full description.

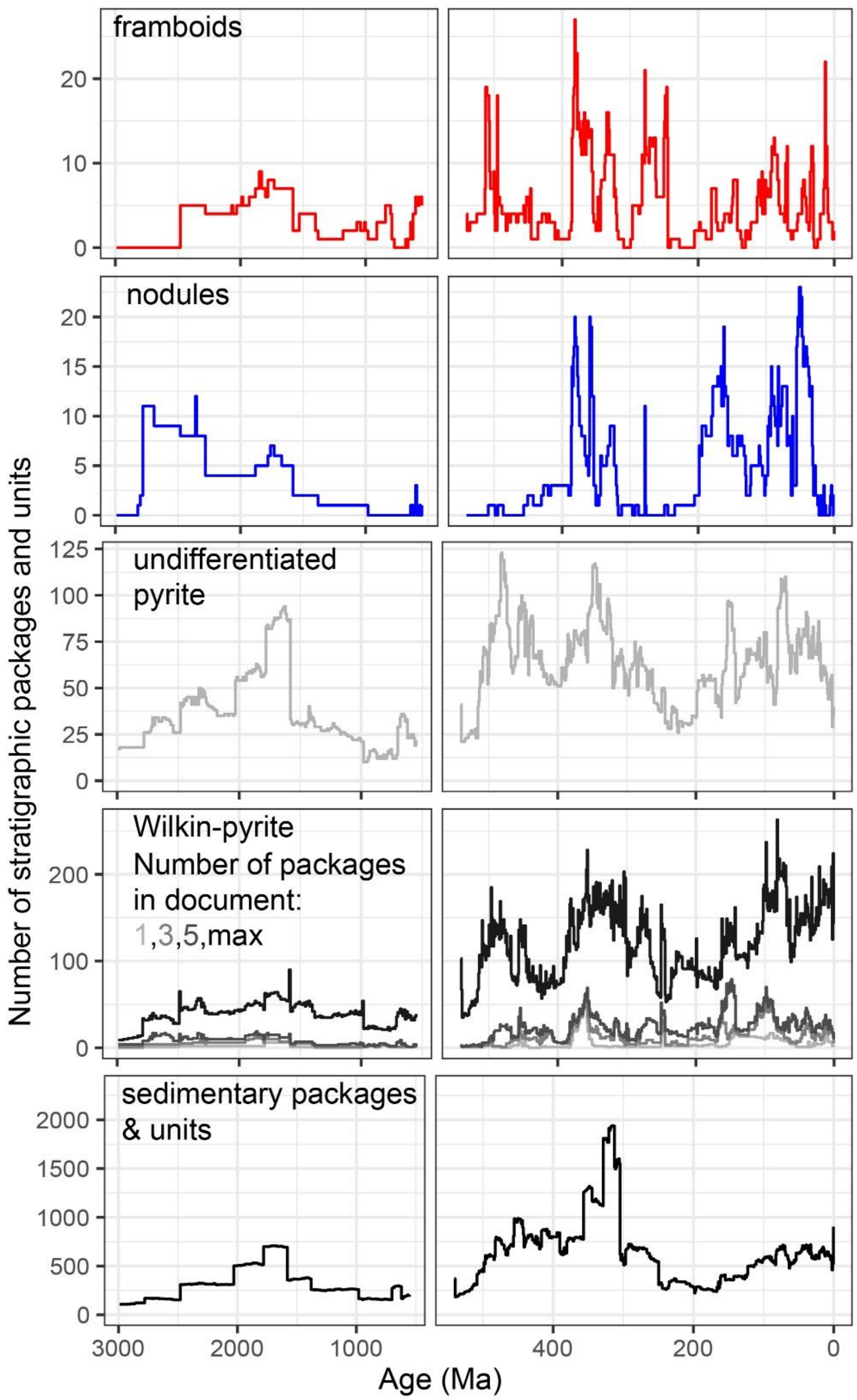

Fig. S4.

The number of pyrite-bearing packages and units, subdivided into framboids, nodules/concretions and undifferentiated pyrite, and total number of (meta)sedimentary packages and units included in the analysis. Note that the large increase in number of units at just over 300 Ma reflects the fine stratigraphic subdivision of Carboniferous cyclothems in the Macrostrat dataset.

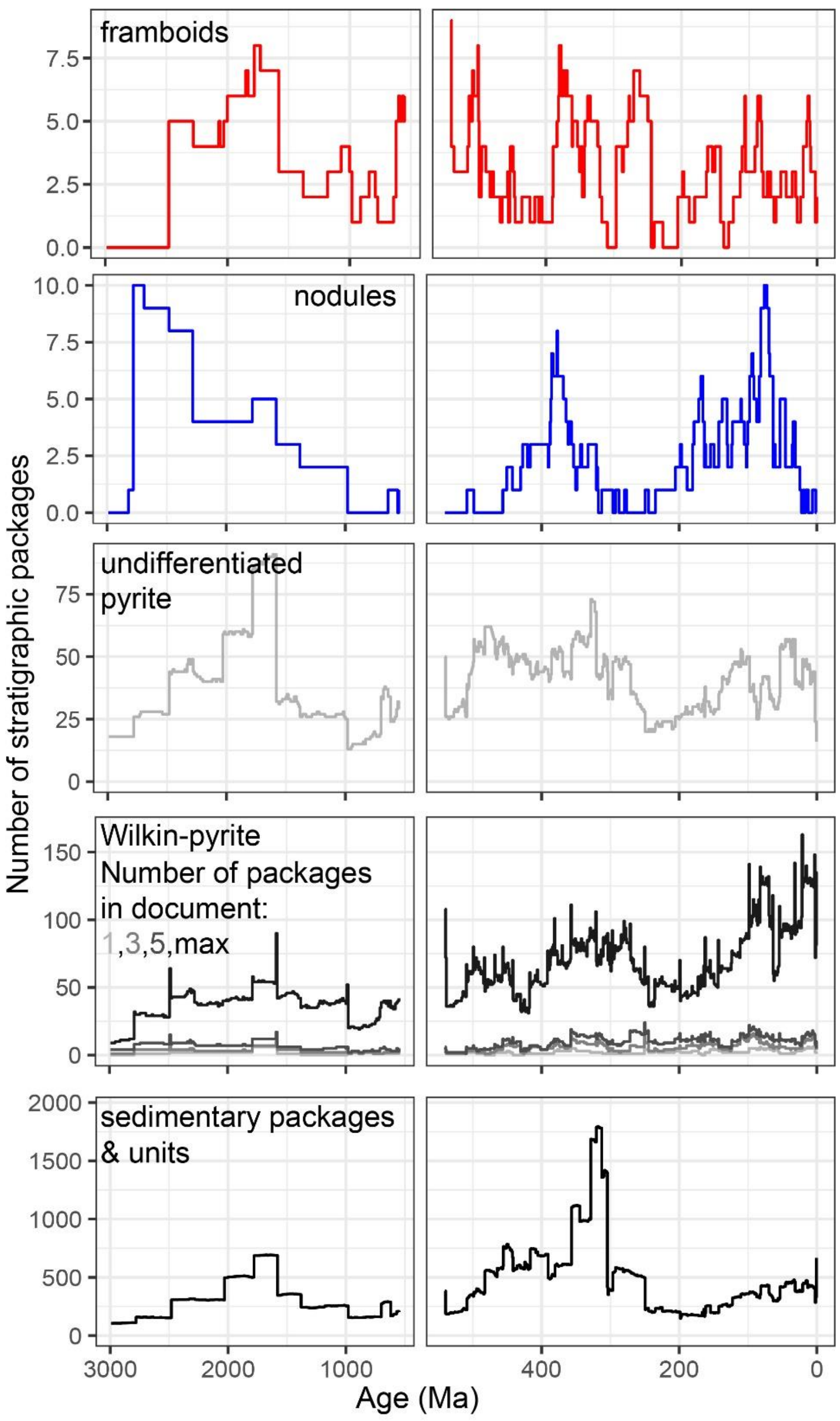

Fig. S5.

The number of pyrite-bearing packages, subdivided into framboids, nodules/concretions and undifferentiated pyrite, and total number of (meta)sedimentary packages included in the analysis. Note that the large increase in number of packages at just over 300 Ma reflects the fine stratigraphic subdivision of Carboniferous cyclothems in the Macrostrat dataset.

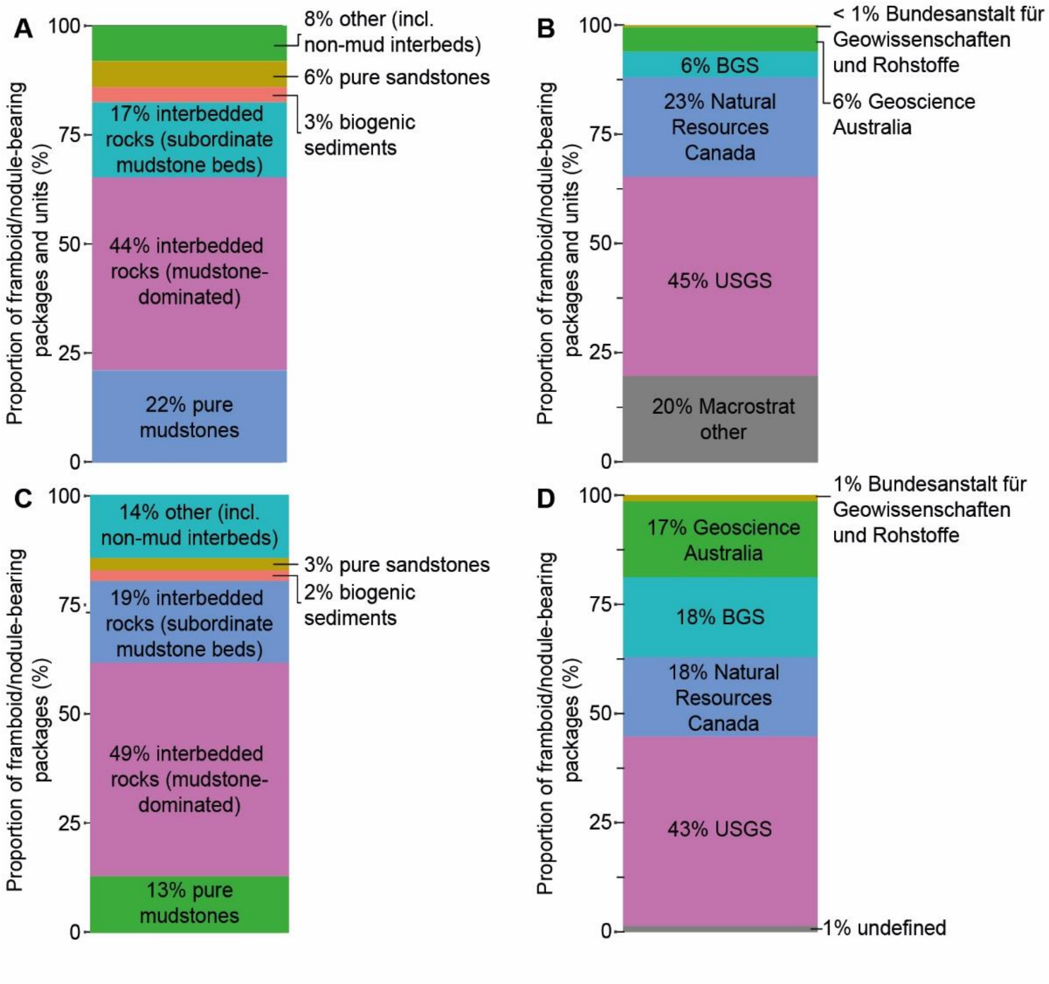

Fig. S6.

Pyrite framboid and concretion/nodule-bearing (meta)sedimentary package and unit lithological descriptions and associated stratigraphic lexicon sources. (A-B) Framboid and concretion/nodule-bearing (meta)sedimentary package and unit (composite) lithological descriptions and lexicon sources. (C-D) Pyrite framboid and concretion/nodule-bearing (meta)sedimentary package lithological descriptions and lexicon sources.

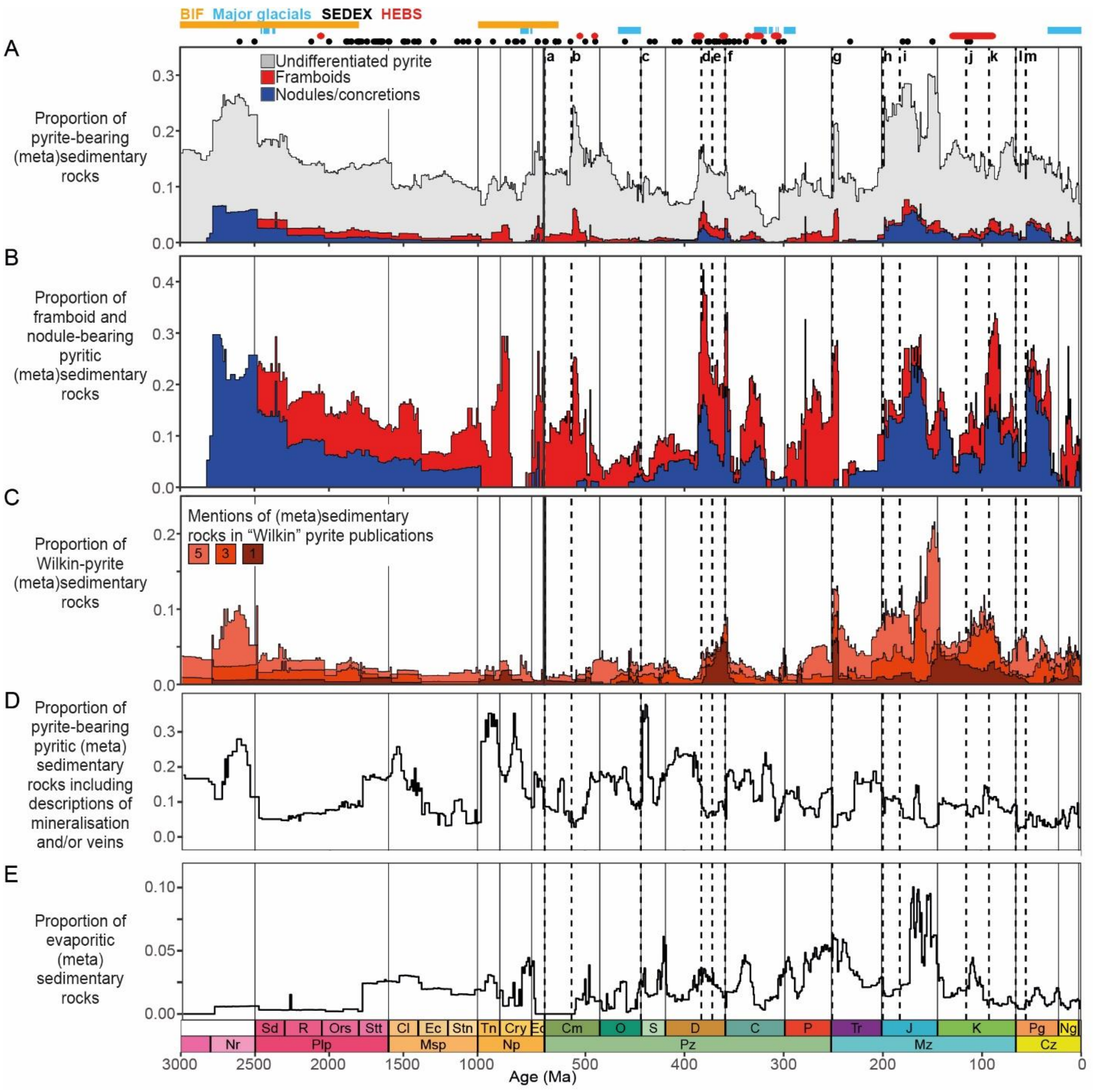

Fig. S7.

(A-C) Composite record (packages and units) of framboid, nodule/concretion and undifferentiated pyritic (meta)sedimentary rocks through geological time (as in Fig. 1). (D) Estimated proportion of pyrite-bearing pyritic (meta)sedimentary rocks related to mineralization and/or veins. (E) Estimated proportion of evaporitic (meta)sedimentary rocks.

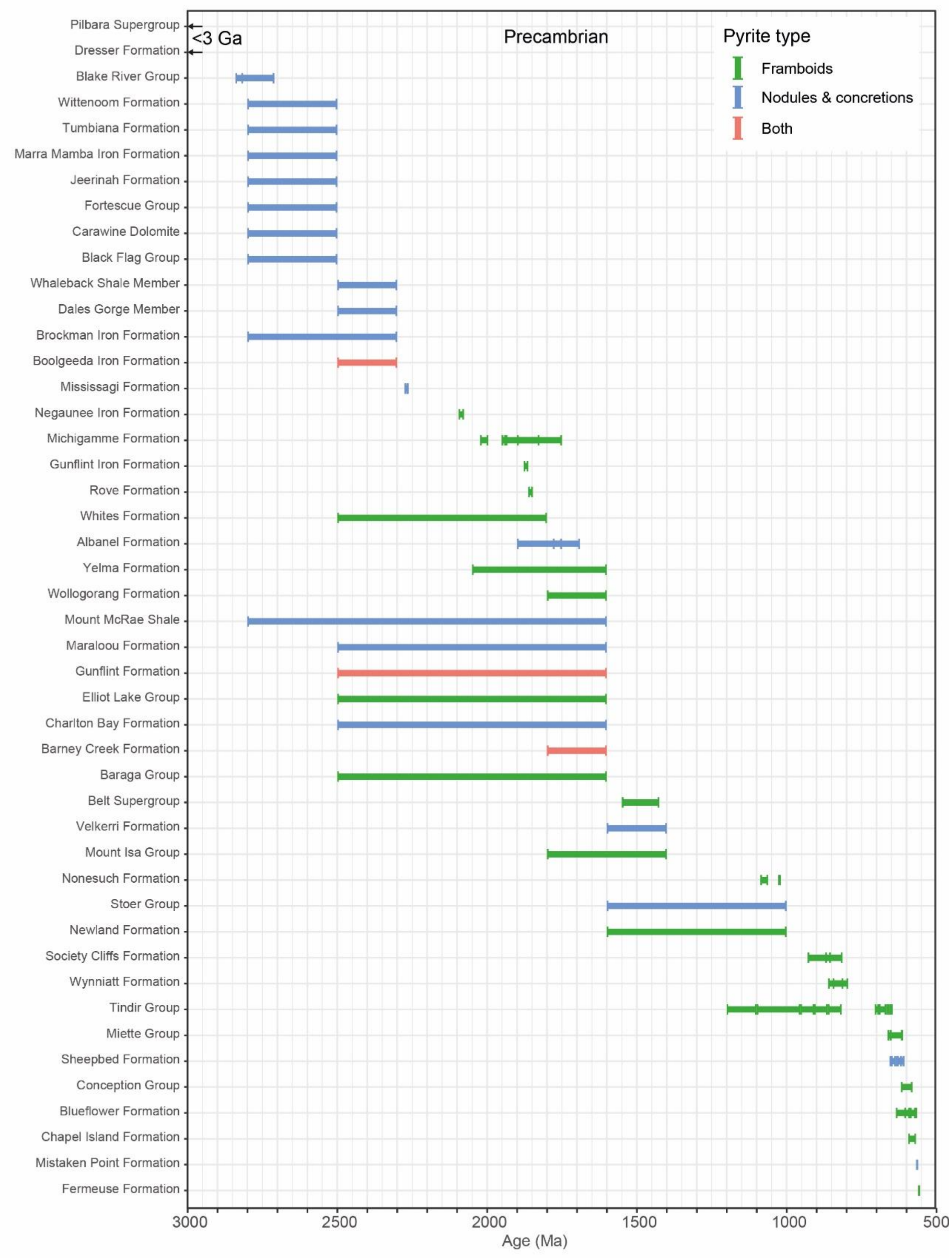

Fig. S8.

Stratigraphic ranges for pyrite framboid and/or nodule-bearing (meta)sedimentary rocks in the Precambrian. Not including undifferentiated pyrite mentions. Internal tick marks indicate unit boundaries where available.

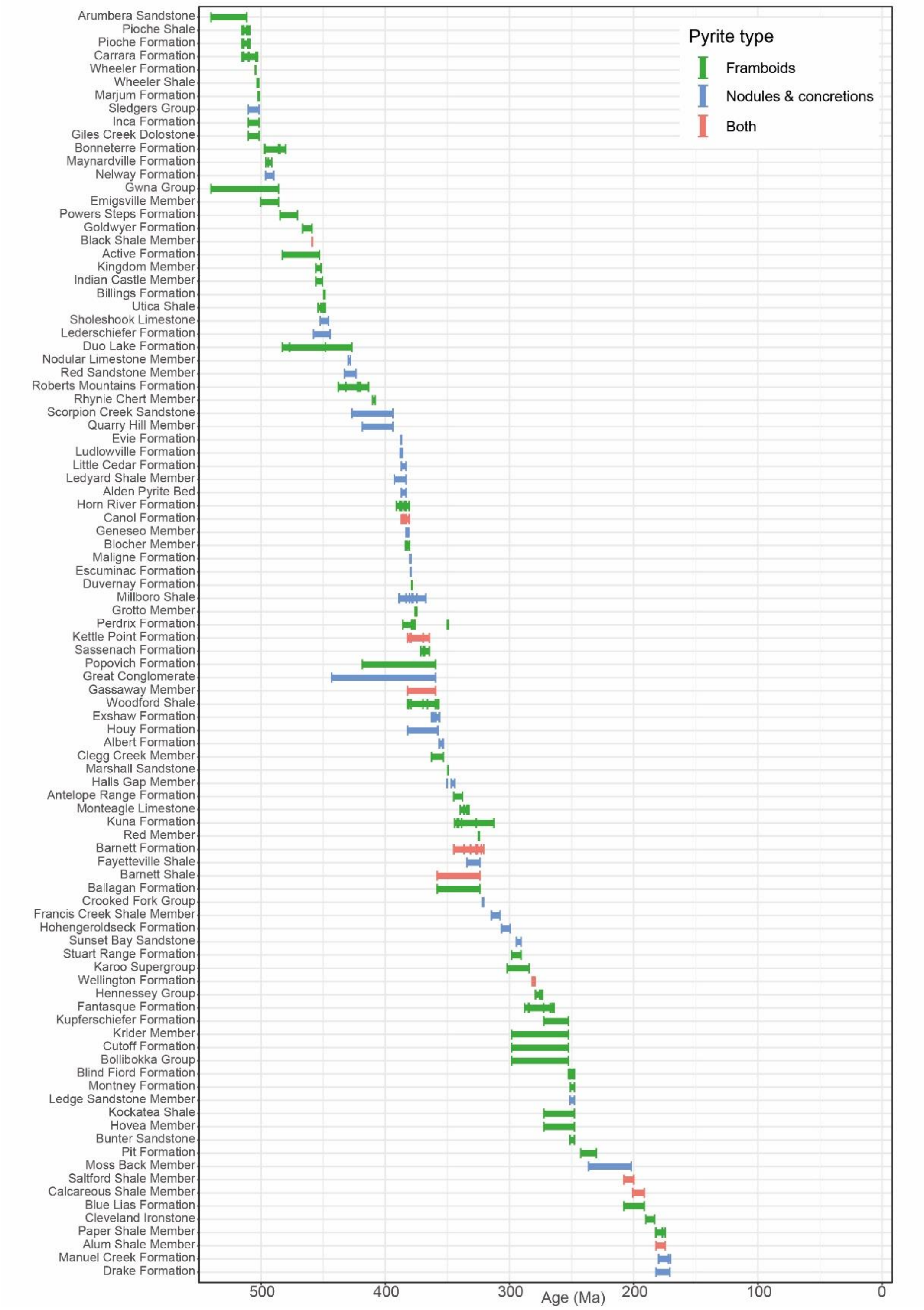

Fig. S9.

Stratigraphic ranges for pyrite framboid and/or nodule-bearing (meta)sedimentary rocks in the Phanerozoic. Not including undifferentiated pyrite mentions. Internal tick marks indicate unit boundaries where available.

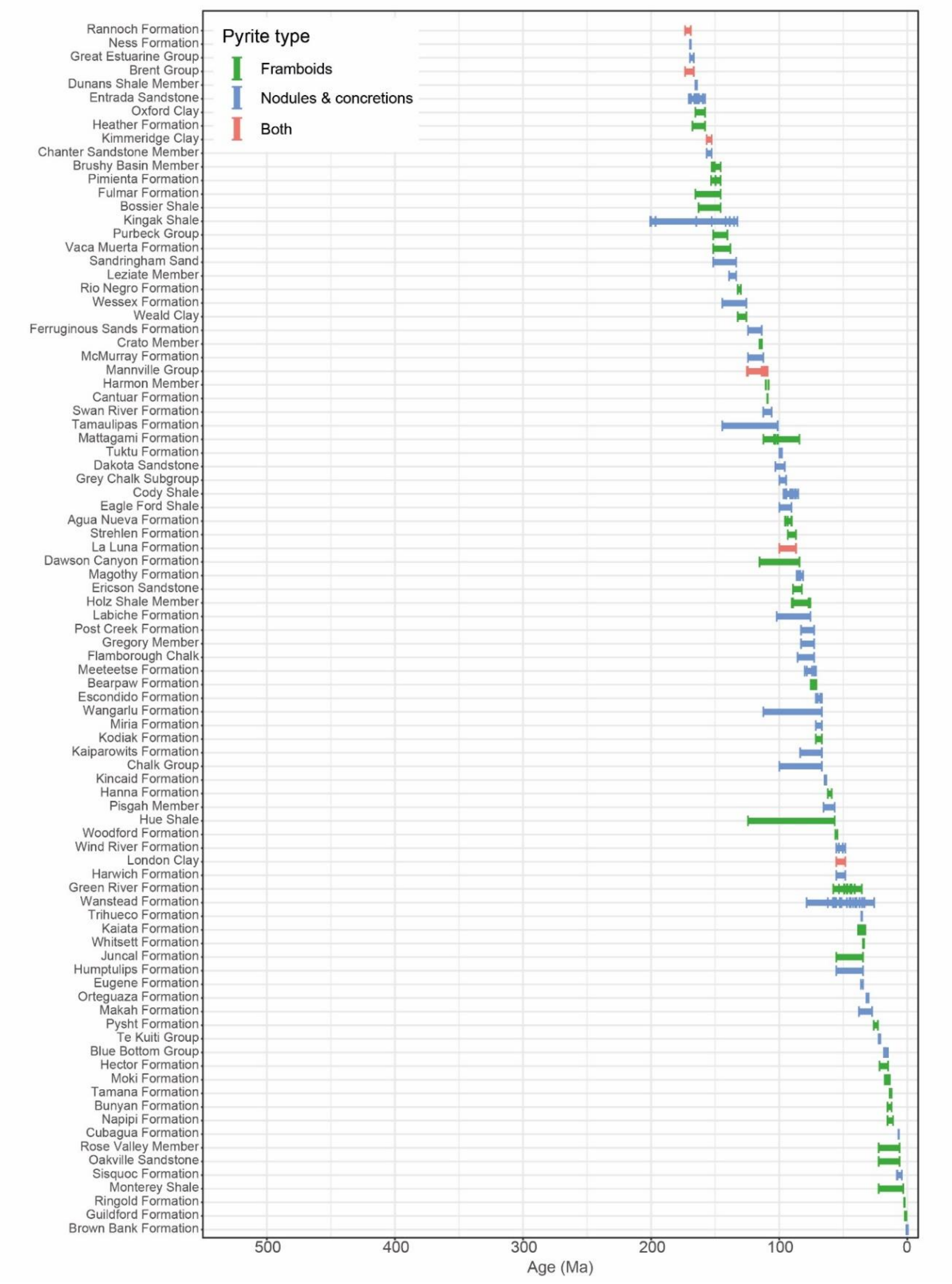

Fig. S10.

Stratigraphic ranges for pyrite framboid and/or nodule-bearing (meta)sedimentary rocks in the Phanerozoic (continued). Not including undifferentiated pyrite mentions. Internal tick marks indicate unit boundaries where available.

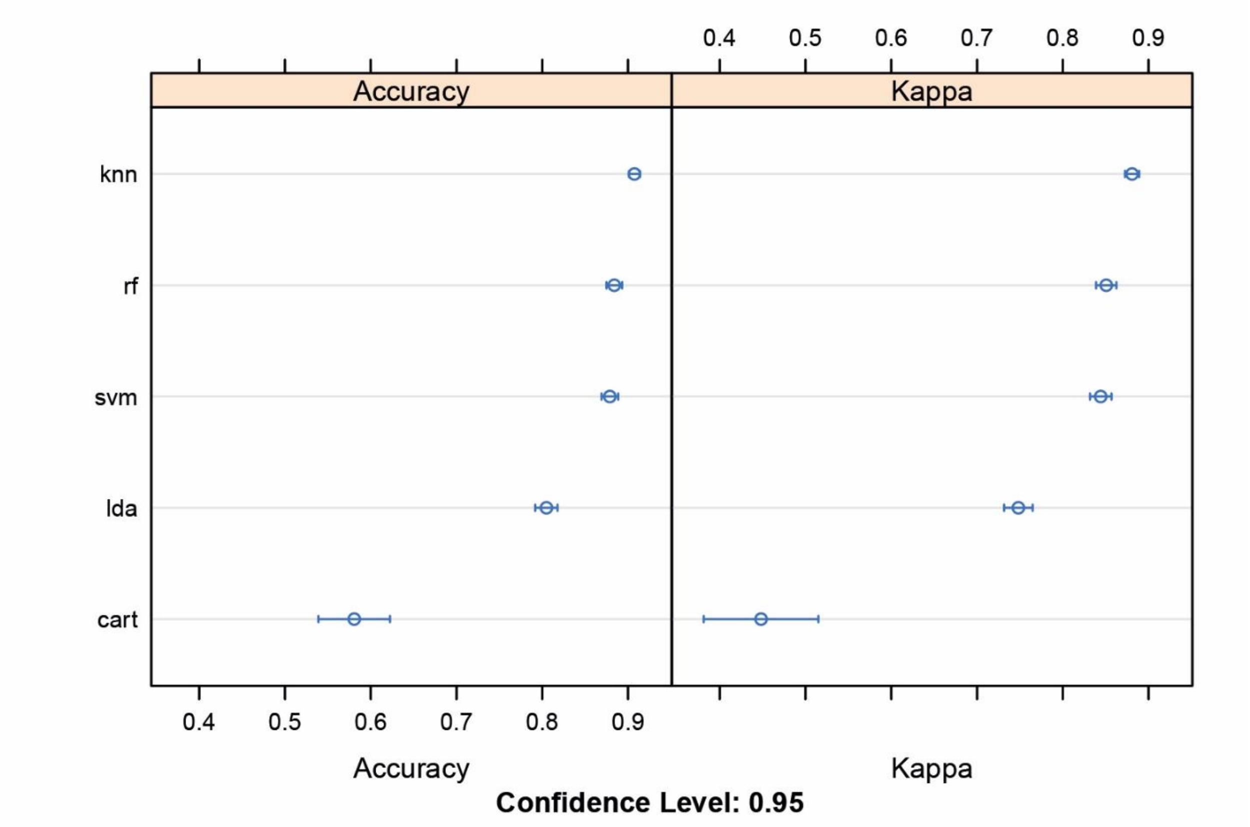

Fig. S11.

The results of several machine learning models applied to observed pyrite compositions *(19-23)* reduced into the first 7 principal components (PCs). The k-nearest neighbour (kNN) algorithm was selected on the basis of favourable accuracy and kappa. Other models tested: Random Forest (rf), Support Vector Machine (SVM), linear discriminant analysis (LDA) and Classification and Regression Trees (CART).

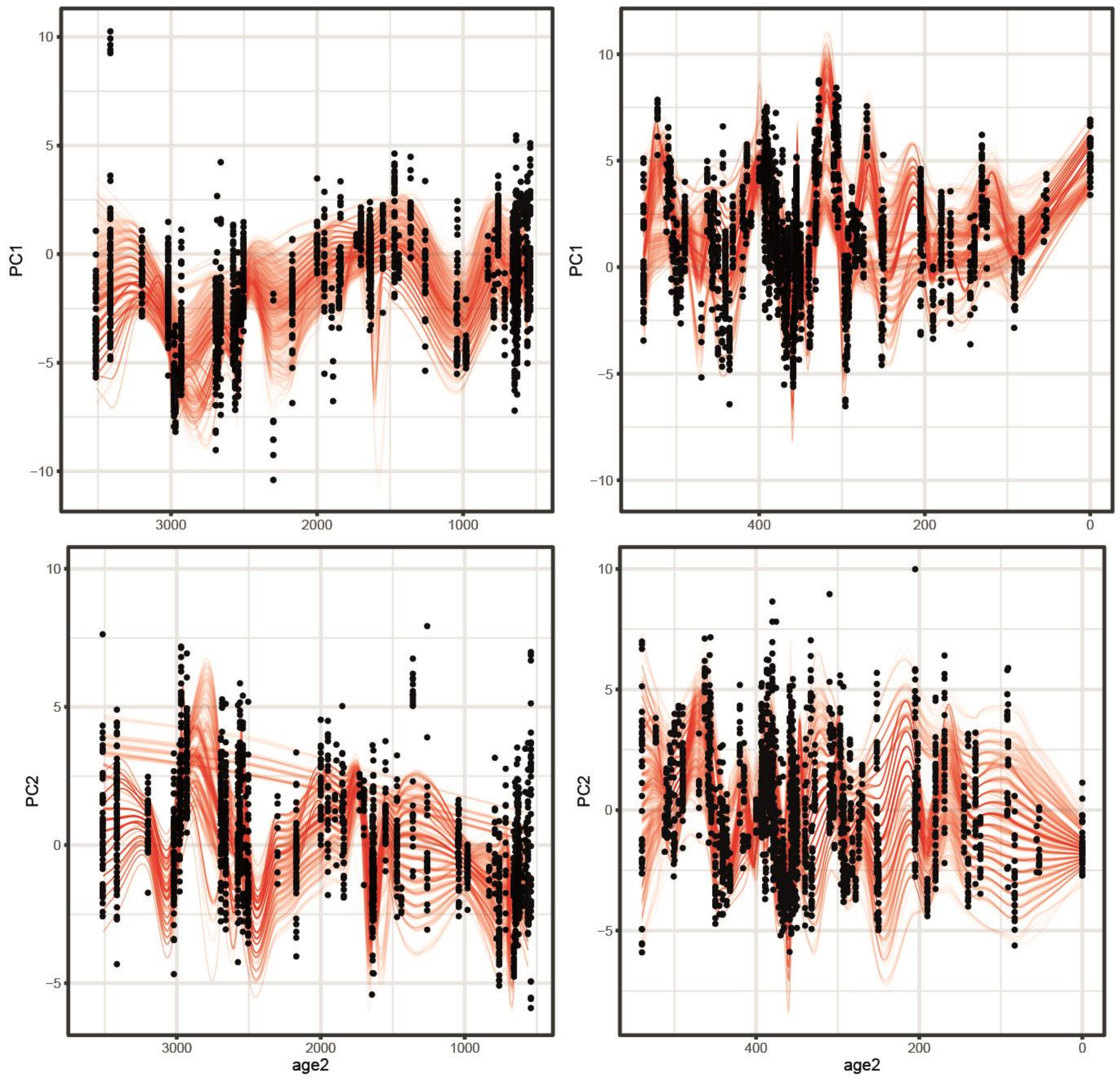

Fig. S12.

Local Polynomial Regression (loess) regressions (± 2σ) for *clr*-transformed pyrite *(19-23)* scores for principal components (PCs) 1-2, with moving cross-validated (CV) loess spans. This approach yields a more smoothed record and is used for the basis of Figure 5. The process was repeated for PCs 3-7 (not shown). We also computed loess resamples using a consistent span using the mean loess CV span (for the Precambrian and Phanerozoic; see Figures S21-S22). Overall, the interpretations do not significantly differ between the two approaches.

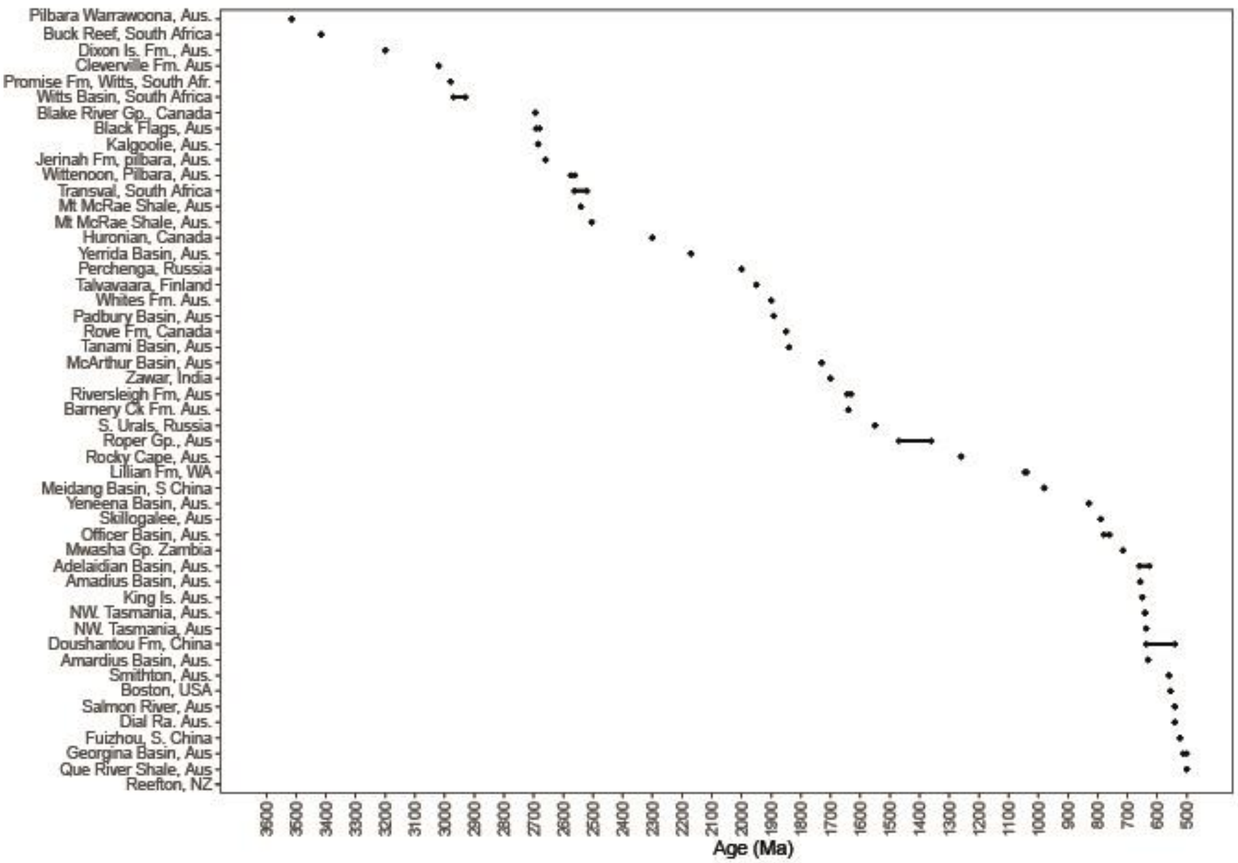

Fig. S13.

Precambrian pyrite sample age ranges by location, as reported in ref. *23*.

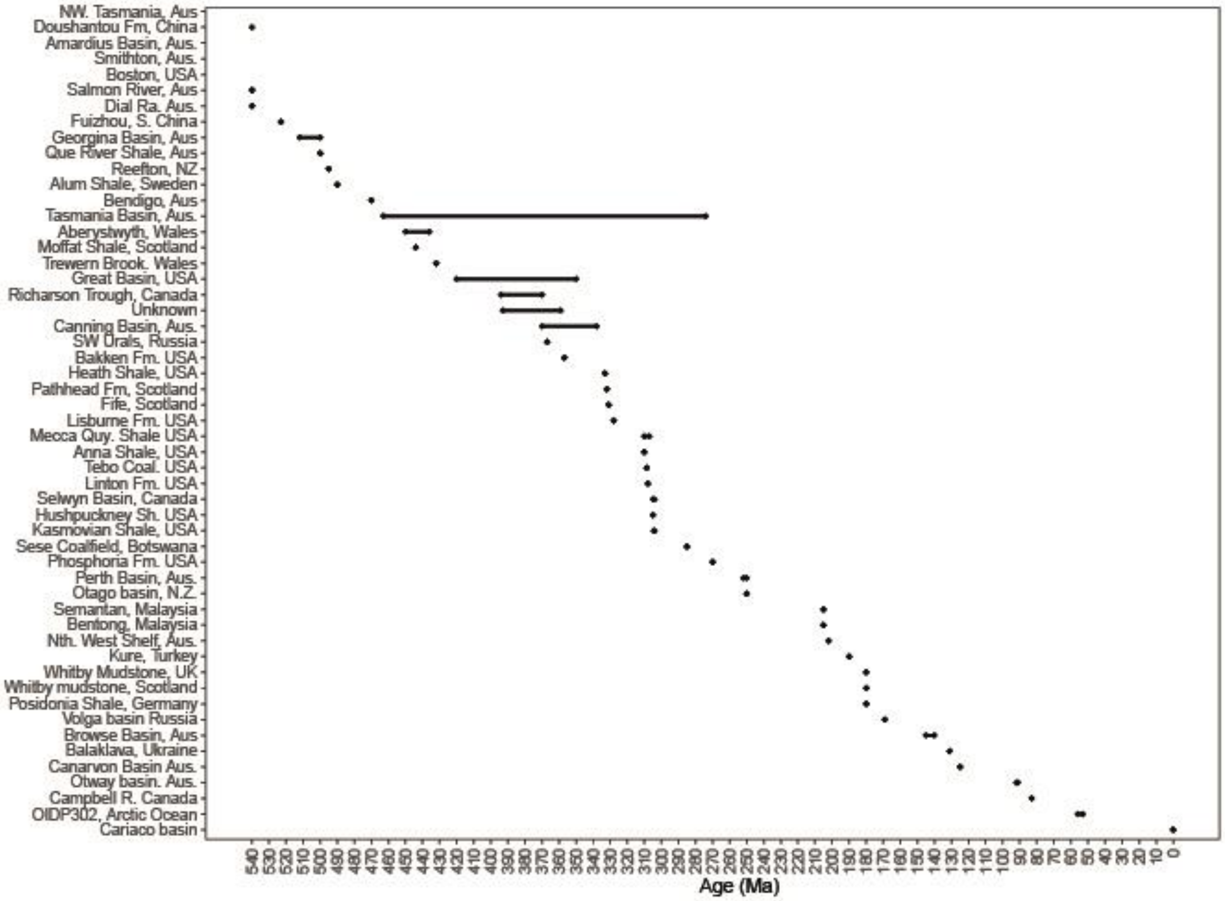

Fig. S14.

Phanerozoic pyrite sample age ranges by location, as reported in ref. *23*.

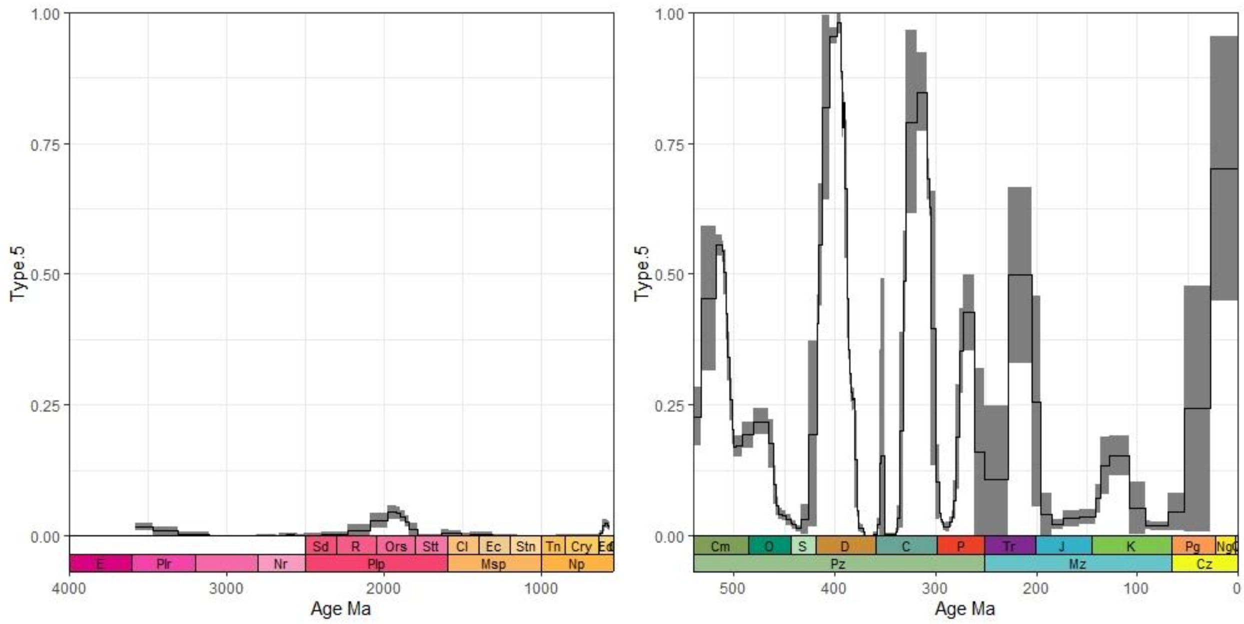

Fig. S15.

Predicted fraction of pyrite type 5 through time (Ma) (± 2σ)

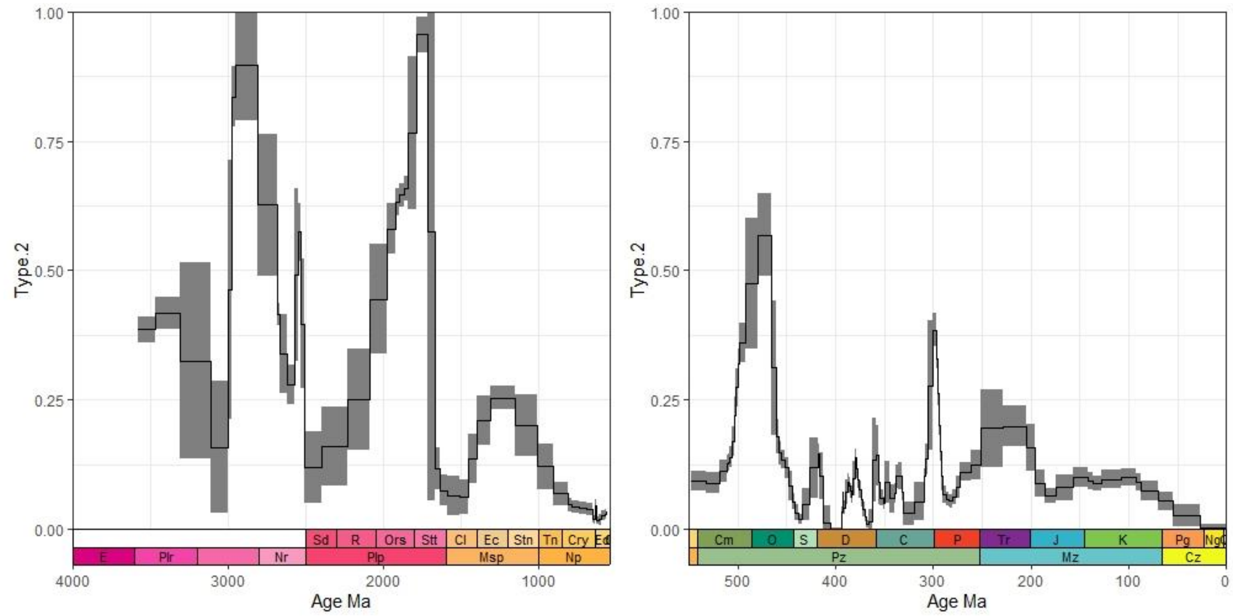

Fig. S16.

Predicted fraction of pyrite type 2 through time (Ma) (± 2σ)

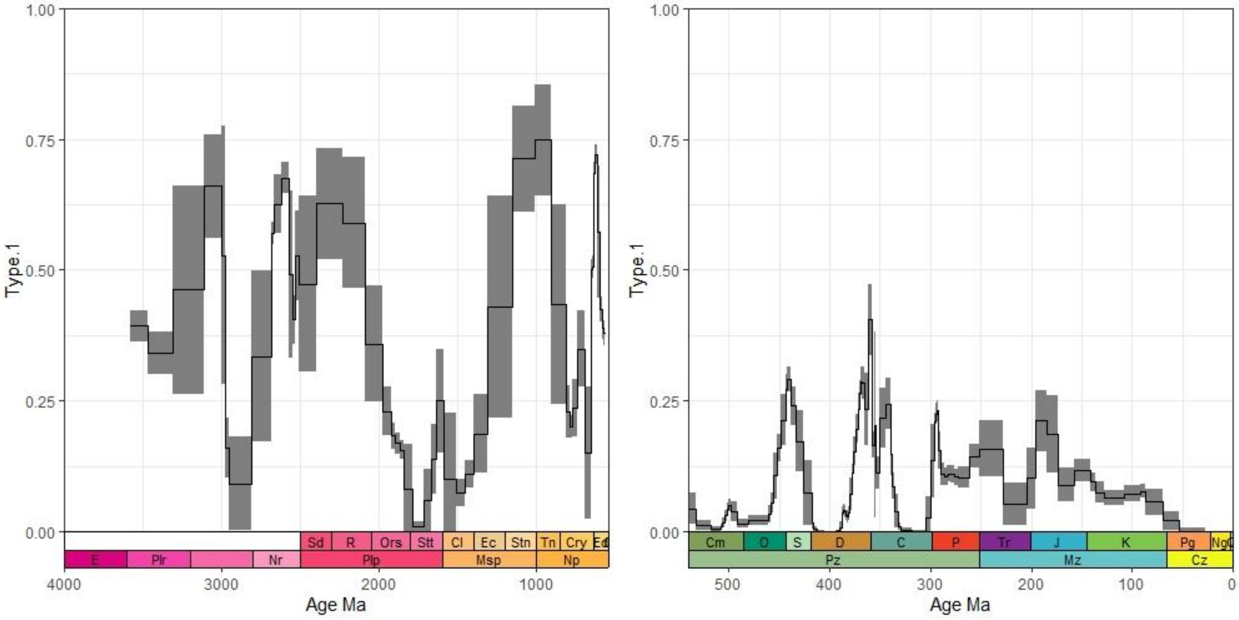

Fig. S17.

Predicted fraction of pyrite type 1 through time (Ma) (± 2σ)

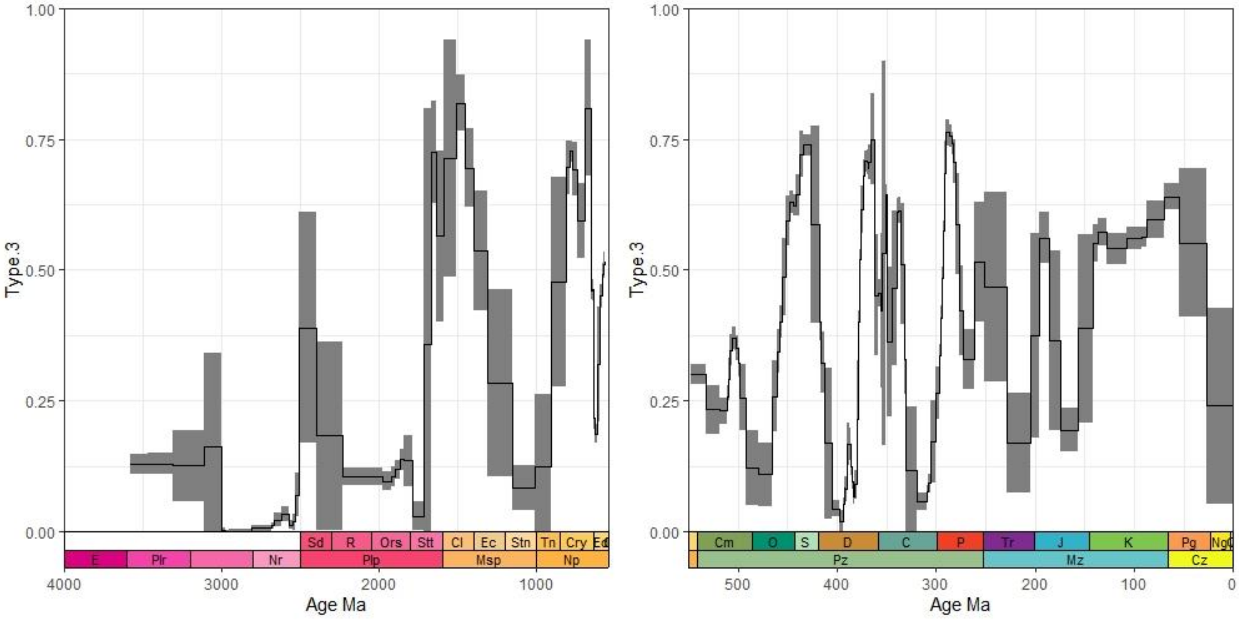

Fig. S18.

Predicted fraction of pyrite type 3 through time (Ma) (± 2σ)

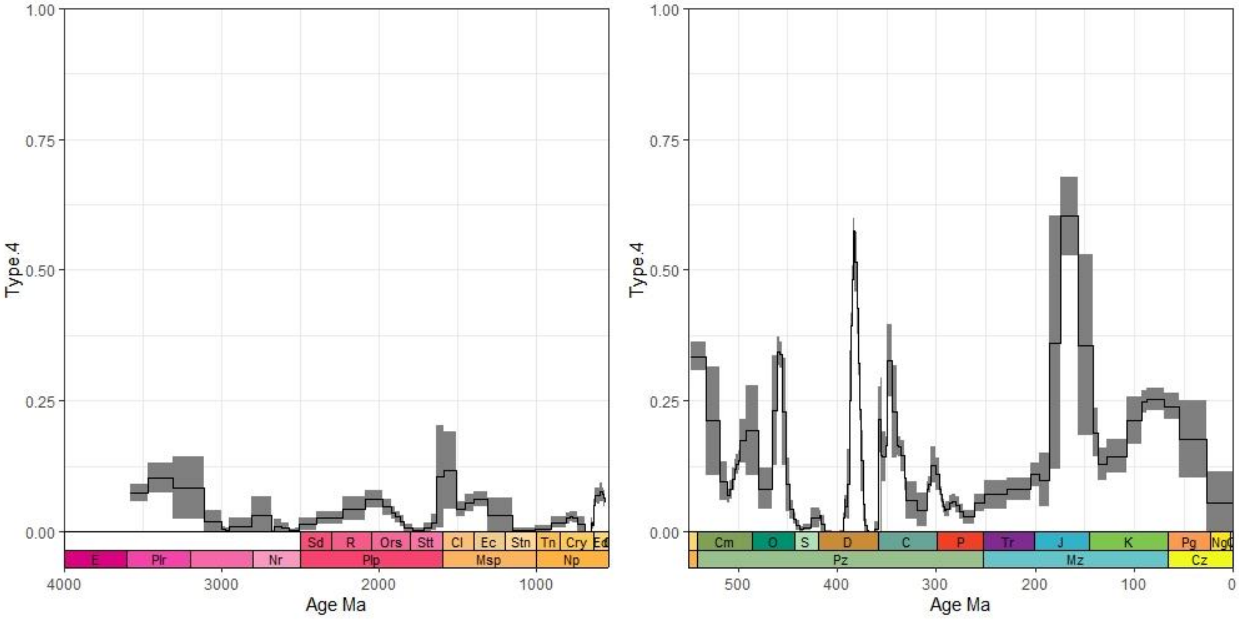

Fig. S19.

Predicted fraction of pyrite type 4 through time (Ma) (± 2σ).

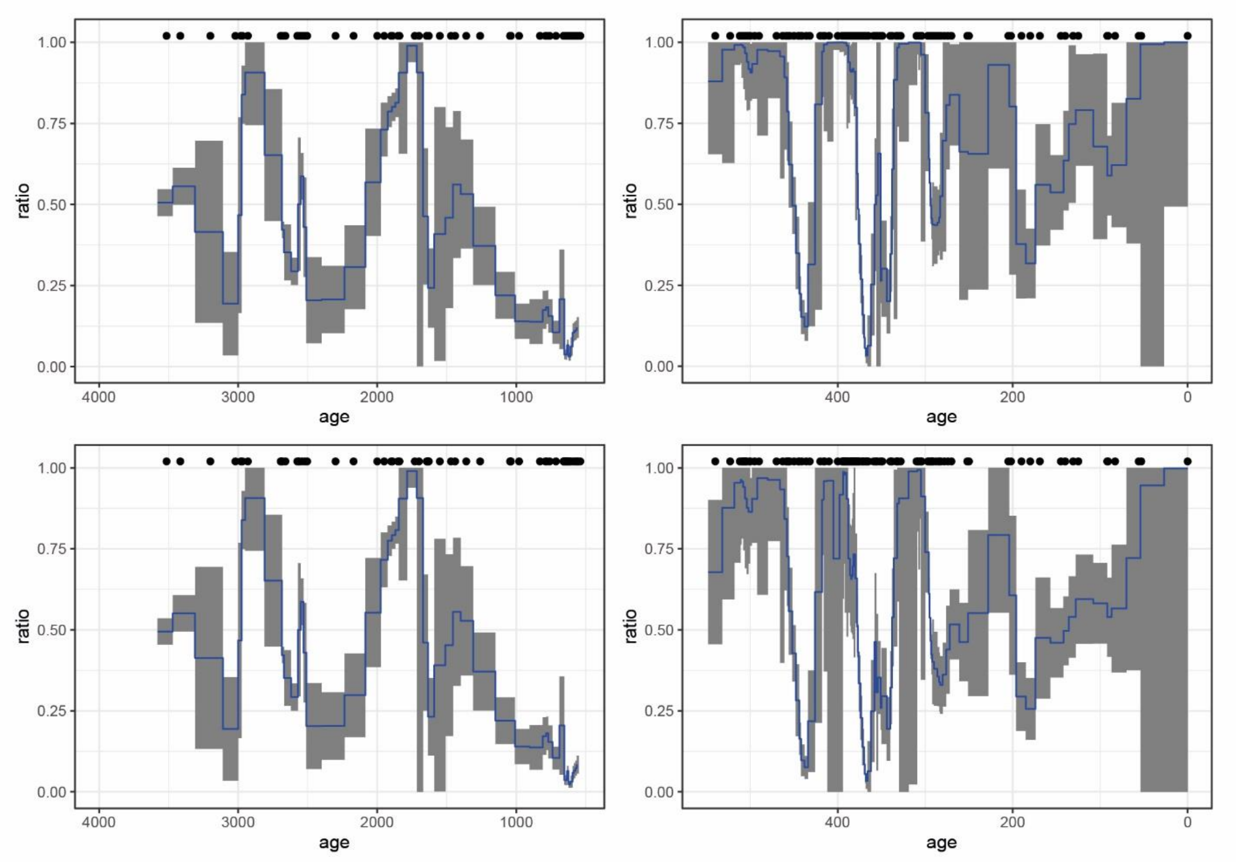

Fig. S20.

Top row: the ratio of type 5+type 2 pyrite versus type 1 pyrite, as presented on Figure 4E. Bottom row: the ratio of type 2 pyrite versus type 1 pyrite. Here the type 5 pyrite fraction is excluded due to a potentially uncertain contribution of Fe oxide versus dissolved Fe during pyrite precipitation (assuming Mn-Fe-OM dynamics). There are two key conclusions from this comparison. (1) the records are similar (away from type 5-dominated intervals), increasing confidence in the general interpretations. (2) the interpreted Fe oxide versus dissolved Fe trends based solely on type 2 versus type 1 pyrite (bottom row) consistently rise to the margins of type 5 POM intervals. In the scenario that Mn-Fe-OM dynamics control pyrite compositions, this signal favours Fe oxide reactants (rather than dissolved Fe) during precipitation of type 5 pyrite.

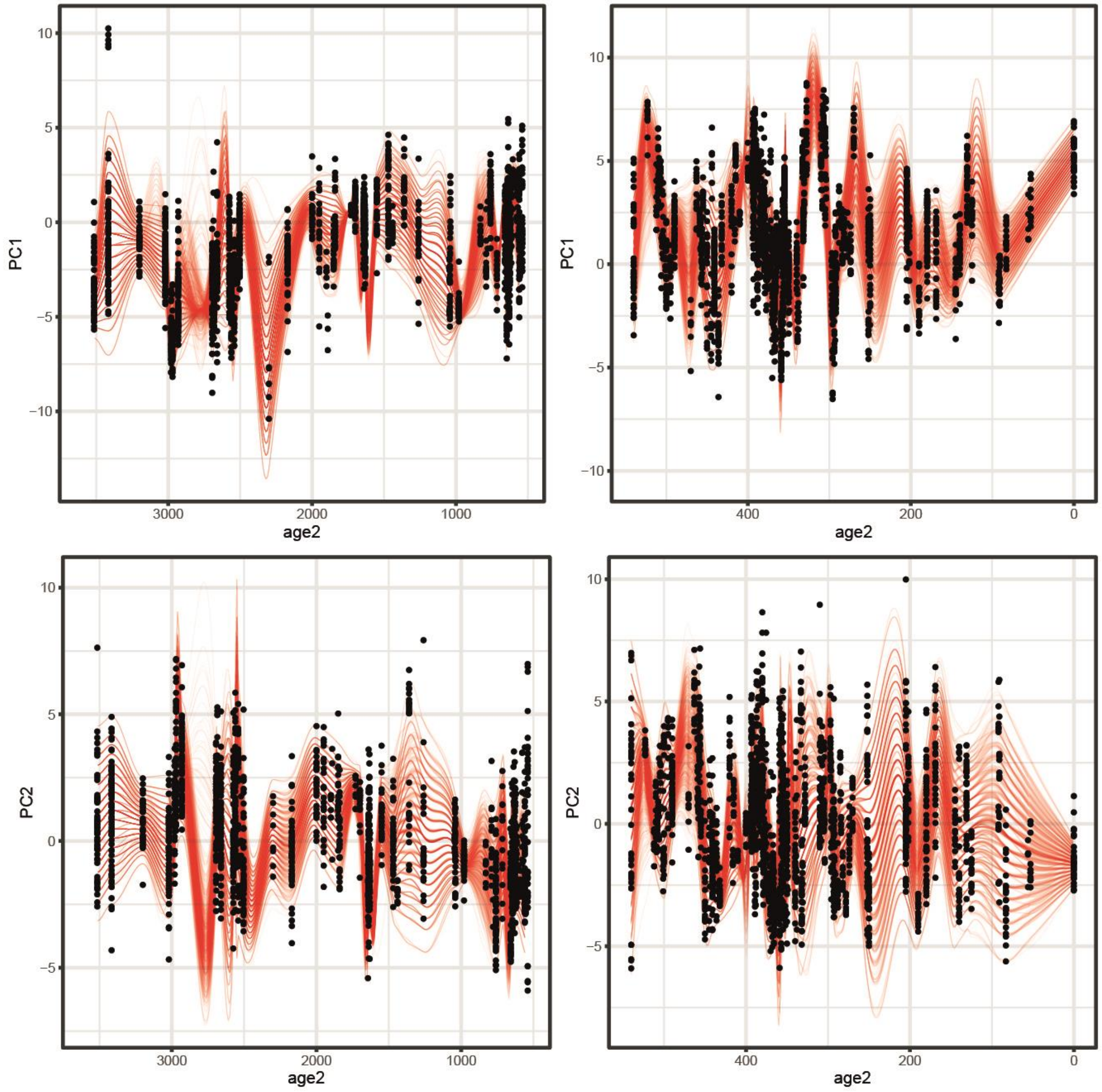

Fig. S21.

Local Polynomial Regression (loess) regressions (± 2σ) for *clr*-transformed pyrite *(19-23)* scores for principal components (PCs) 1-2, with a single cross-validated (CV) loess span for the Precambrian and Phanerozoic. This produces a more coherent output that is more sensitive to local variation. The process was repeated for PCs 3-7 (not shown).

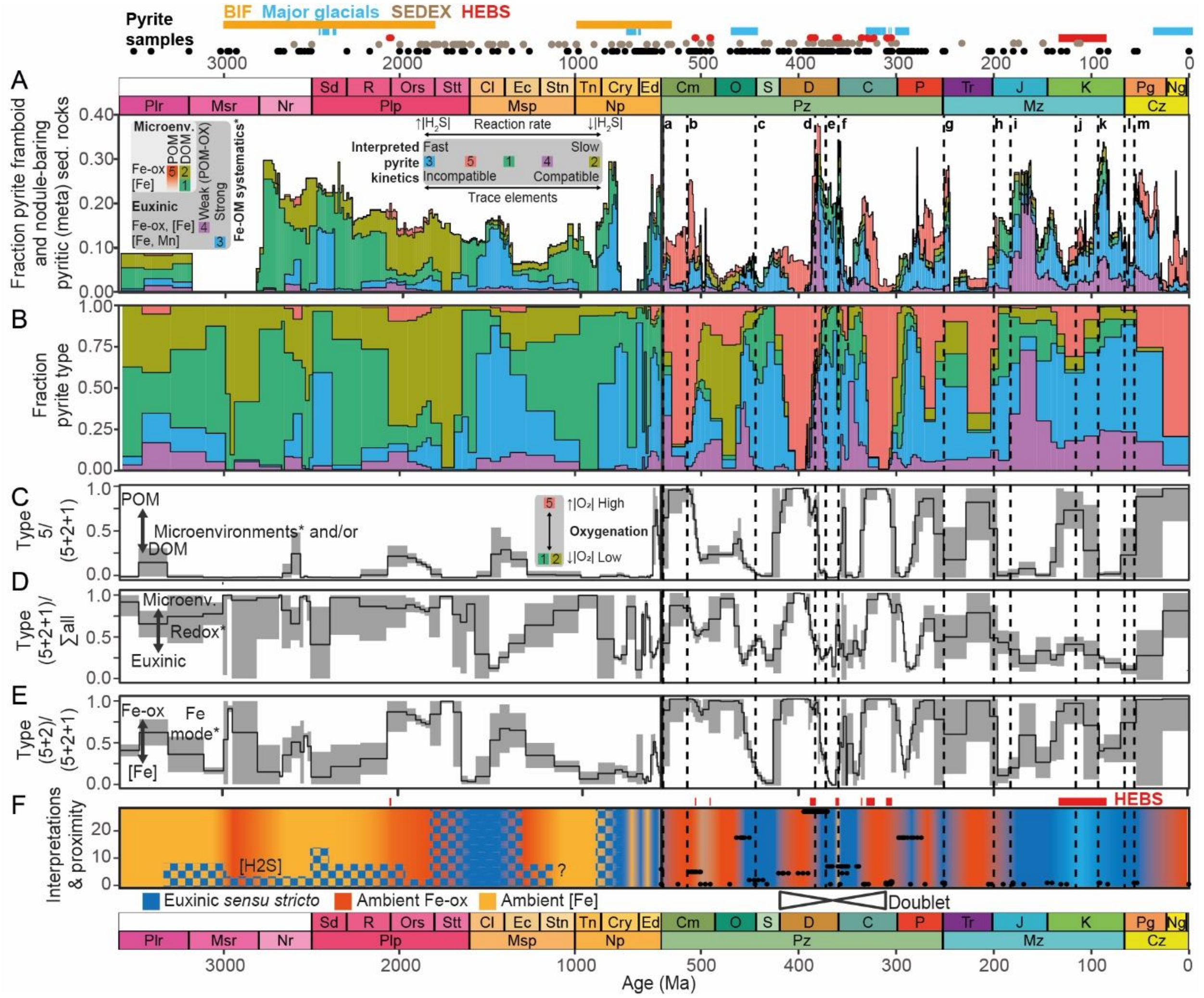

Fig. S22.

Pyrite predictions based on a single cross-validated (CV) loess span for the Precambrian and Phanerozoic (Fig. S21). This produces a more coherent output that is more sensitive to short wavelength variation. The process was repeated for PCs 3-7 (not shown). Both approaches (Figure 4, this figure) support similar interpretations (F).

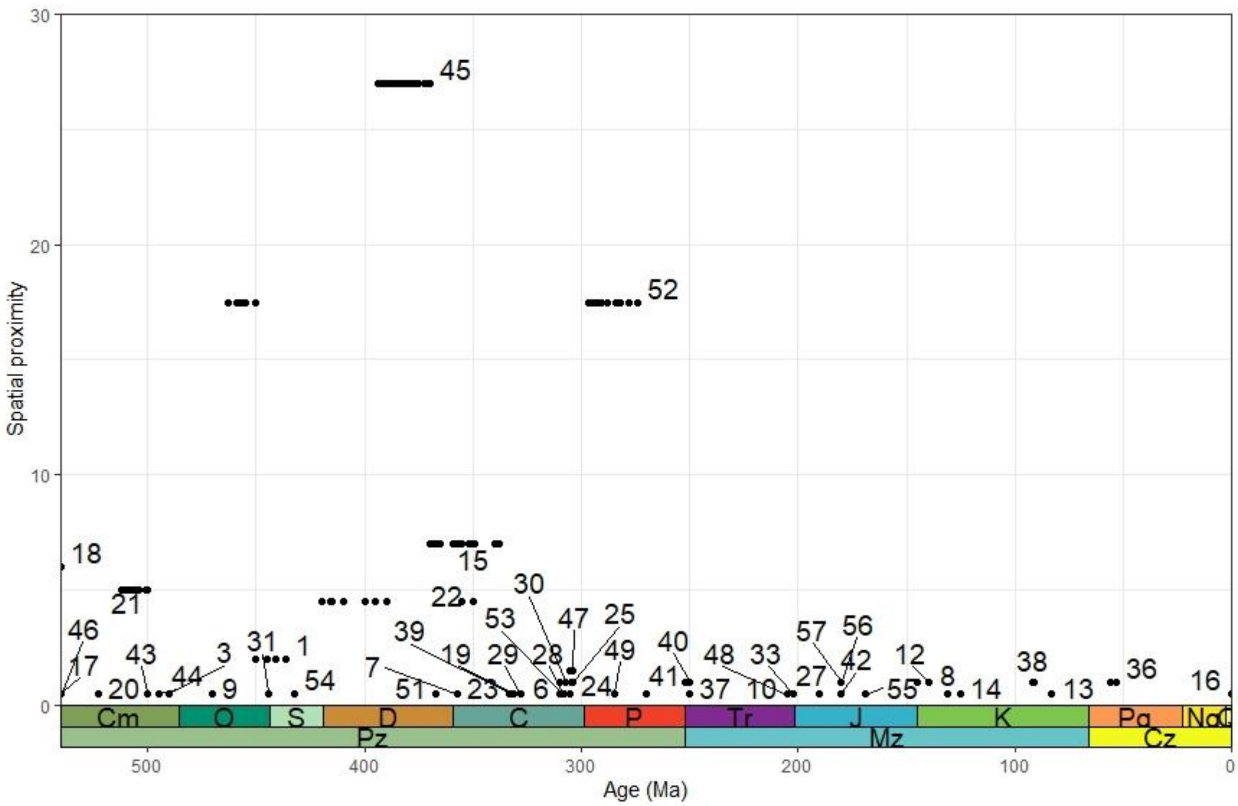

Fig. S23.

Spatial proximities for Phanerozoic pyrite trace element analyses reported by refs. (19-23). Labels: Aberystwyth, Wales (1), Adelaidian Basin, Aus. (2), Alum Shale, Sweden (3), Amadius Basin, Aus. (4), Amardius Basin, Aus. (5), Anna Shale, USA (6), Bakken Fm. USA (7), Balaklava, Ukraine (8), Bendigo, Aus (9), Bentong, Malaysia (10), Boston, USA (11), Browse Basin, Aus (12), Campbell R. Canada (13), Canarvon Basin Aus. (14), Canning Basin, Aus. (15), Cariaco basin (16), Dial Ra. Aus. (17), Doushantou Fm, China (18), Fife, Scotland (19), Fuizhou, S. China (20), Georgina Basin, Aus (21), Great Basin, USA (22), Heath Shale, USA (23), Hushpuckney Sh. USA (24), Kasmovian Shale, USA (25), King Is. Aus. (26), Kure, Turkey (27), Linton Fm. USA (28), Lisburne Fm. USA (29), Mecca Quy. Shale USA (30), Moffat Shale, Scotland (31), Mwasha Gp. Zambia (32), Nth. West Shelf, Aus. (33), NW. Tasmania, Aus (34), NW. Tasmania, Aus. (35), OIDP302, Arctic Ocean (36), Otago basin, N.Z. (37), Otway basin. Aus. (38), Pathhead Fm, Scotland (39), Perth Basin, Aus. (40), Phosphoria Fm. USA (41), Posidonia Shale, Germany (42), Que River Shale, Aus (43), Reefton, NZ (44), Richarson Trough, Canada (45), Salmon River, Aus (46), Selwyn Basin, Canada (47), Semantan, Malaysia (48), Sese Coalfield, Botswana (49), Smithton, Aus. (50), SW Urals, Russia (51), Tasmania Basin, Aus. (52), Tebo Coal. USA (53), Trewern Brook. Wales (54), Volga basin Russia (55), Whitby mudstone, Scotland (56), Whitby Mudstone, UK (57).

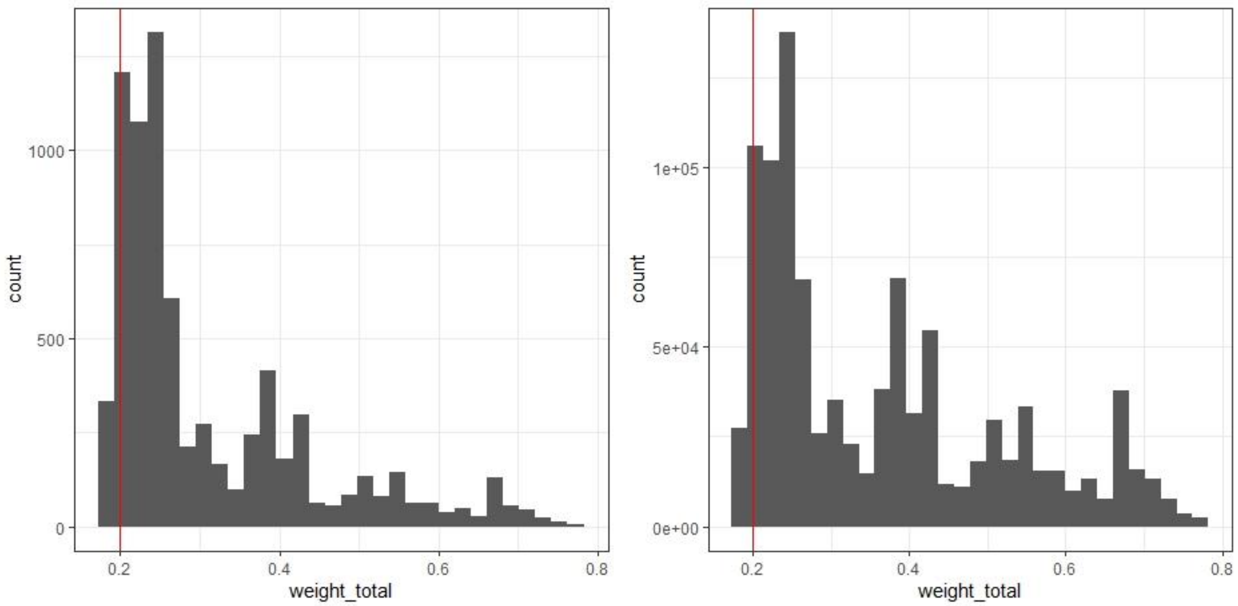

Fig. S24.

Sample weighting (probability of selection) for the composite xDD, pyrite trace elements and SGP redox dataset (as in Fig. 6B). The 1-in-5 probability is highlighted (red). Left: prior to resampling. Right: following resampling with replacement (n = 1,000,000). Note how the distribution becomes more balanced following resampling, because samples with larger weights are more likely to be sampled. The resampled version was used for spatial interpolation.
